# Periconceptional and prenatal exposure to metal mixtures in relation to behavioral development at 3 years of age

**DOI:** 10.1097/EE9.0000000000000106

**Published:** 2020-07-06

**Authors:** Brett T. Doherty, Megan E. Romano, Jiang Gui, Tracy Punshon, Brian P. Jackson, Margaret R. Karagas, Susan A. Korrick

**Affiliations:** a Department of Epidemiology, Geisel School of Medicine at Dartmouth, Hanover, New Hampshire; b Department of Biomedical Data Science, Geisel School of Medicine at Dartmouth, Hanover, New Hampshire; c Department of Biological Sciences, Dartmouth College, Hanover, New Hampshire; d Department of Earth Sciences, Dartmouth College, Hanover, New Hampshire; e Channing Division of Network Medicine, Brigham and Women’s Hospital, Boston, Massachusetts; f Department of Environmental Health, Harvard TH Chan School of Public Heath, Boston, Massachusetts

**Keywords:** Neurodevelopment, Behavior, Mixtures, Metals, Critical windows, Prenatal exposures

## Abstract

Behavioral effects of prenatal exposure to mixtures of essential and toxic metals are incompletely understood.

**Methods:**

We investigated neurodevelopmental effects of prenatal metal exposures in the New Hampshire Birth Cohort Study, a prospective birth cohort. We measured metals (As, Cu, Mn, Pb, Se, Zn) in maternal prenatal and postnatal toenails and infant toenails, reflecting exposures during periconception and early pregnancy, mid pregnancy, and late pregnancy and early neonatal life, respectively. Mothers completed the Social Responsiveness Scale, 2nd ed. (SRS-2) and the Behavior Assessment System for Children, 2nd ed. (BASC-2) to assess their child’s neurobehavior at 3 years. We used mean field variational Bayes for lagged kernel machine regression to investigate associations of toenail metal concentrations with SRS-2 Total Scores and BASC-2 composite scores (SRS-2: n = 371; BASC-2: n = 318).

**Results:**

Infant toenail Mn was associated with poorer performance on multiple BASC-2 composite scores. Maternal postnatal toenail As was associated with worse scores on the BASC-2 Internalizing Problems and Behavioral Symptoms Index. Associations with Mn were generally stronger in males, and associations with As were generally stronger in females. Other metals, including Pb, were weakly or inconsistently associated with BASC-2 outcomes, and there were no strong associations of individual metals and SRS-2 Total Scores. We observed suggestive evidence of interaction between As and Se for SRS-2 Total Scores and BASC-2 Adaptive Skills, though overall evidence of interactions between metals was weak.

**Conclusion:**

Our results support the hypothesis that exposure to Mn and As in mid to late pregnancy may be neurodevelopmentally harmful.

## Abstract 2

Supplemental Digital Content is available in the text.

**What this study adds**

In a prospective pregnancy cohort, we investigated prenatal exposures to time-varying mixtures of essential and toxic metals in relation to early childhood behavioral development. We observed that Mn and As may be developmental neurotoxicants and that exposures to these metals in mid to late pregnancy may be more impactful than exposures earlier in pregnancy. Our study contributes to evidence of adverse neurodevelopmental effects of prenatal metal exposures.

## Introduction

Maternal exposure to essential or toxic metals and metalloids (hereafter, “metals”) during pregnancy is of significant public health concern, as the prenatal period is a uniquely sensitive period of human development.^1–5^ Timing of exposure is critical, as fetal development is an ordered and cumulative process, and exposures at different time points may have varying impacts.^1,2,6^ Metals readily cross the placenta so that maternal exposures during pregnancy result in fetal exposure.^7–9^ Such exposures have been linked to a variety of important health endpoints, including alterations in neurodevelopment.^4,5,10,11^ The risks of adverse neurodevelopmental effects of prenatal exposure to Pb and Hg are well established,^5,12^ and accumulating evidence suggests adverse behavioral effects of prenatal Mn exposure and possibly As.^13–16^

To date, most evidence of neurodevelopmental effects of prenatal metal exposures has been generated by studies of one or two metals measured at a single time point. Interest in neurodevelopmental effects of multimetal mixtures, however, has recently increased with the collection of multimetal exposure data and the development of statistical methods to accommodate such data. Common objectives of these multimetal investigations are to estimate independent effects of metals (while controlling for co-metal confounding), to estimate joint effects of multiple metals, including potential interactions among metals, and often to investigate nonlinear metal-outcome relations.^17,18^ Only recently, however, have epidemiologic investigations begun to consider neurodevelopmental effects of time-varying multimetal mixtures.^19–22^ These investigations are supported by longitudinal data on multimetal exposures that occur during the prenatal period and recently developed statistical methods that extend time-fixed mixtures methods to accommodate longitudinal exposure data. These time-varying multimetal mixture investigations have similar objectives as their time-fixed counterparts, with the additional objective of estimating metal-outcome relations at multiple time points, enabling the identification of sensitive periods of exposure.

Epidemiologic assessment of neurodevelopmental effects of time-varying mixtures of essential and toxic metals is needed to characterize the effects of realistic exposure scenarios and support efforts to provide for optimal fetal development. Thus, there is a need to identify gestational periods during which the fetus is most vulnerable and to identify potentially harmful interactions between toxic metals, or between essential and toxic metals that might inform possible interventions to mitigate harmful effects of toxic metals. We therefore investigated prenatal exposures to metal mixtures during multiple periods of pregnancy in a cohort of mother–child pairs receiving prenatal care in central New Hampshire.

## Methods

### Study population

This study used data from the New Hampshire Birth Cohort Study (NHBCS), an ongoing prospective cohort of mother–child pairs receiving prenatal care in New Hampshire.^23–25^ Beginning in 2009, pregnant women were recruited from participating prenatal care clinics at approximately 24–28 gestational weeks if they met the following eligibility criteria: 18–45 years of age, carrying a singleton pregnancy, literate in English, having a private water system as the primary source of water at their residence, and having resided at the same address since their last menstrual period and intending to remain at that residence throughout the pregnancy. As of November 2019, approximately 2,000 women have been enrolled. Three years after birth (between 2014 and 2019), 1,405 mothers were mailed two behavioral assessments, the Social Responsiveness Scale, 2nd ed. (SRS-2) and the Behavior Assessment System for Children, 2nd ed. (BASC-2). Of these, 749 mothers returned a validly completed SRS-2 form and 650 mothers returned a validly completed BASC-2 form. Of these, a subset had complete metal exposure data (SRS-2: n = 477; BASC-2: n = 410). This analysis includes mother–child pairs with complete data on behavioral assessments, metal exposures, and covariates (SRS-2: n = 371; BASC-2: n = 318) (Supplemental Figure 1; http://links.lww.com/EE/A97). The behavioral assessments, exposure assessments, and covariates are described in further detail below. Study protocols for NHBCS were approved by the Committee for the Protection of Human Subjects at Dartmouth College, and participants provided written informed consent.

### Exposure to metals

We measured concentrations of metals in toenail samples collected at three time periods, including maternal toenails collected at a median of 27 (IQR: 25, 30) gestational weeks and a median of 4 (IQR: 2, 6) weeks postpartum and infant toenails collected at a median of 6 (IQR: 6, 7) weeks of life. In adults, toenail metal concentrations reflect exposures approximately 6–12 months before toenail collection;^24,26,27^ for infants and young children, toenail replacement rates are faster and measured metal concentrations reflect more recent exposures.^24,28^ Specifically, although data on infant toenail growth rates are sparse, available literature supports infant toenail lengths ranging between approximately 5 to 1 mm at birth^29^ and a growth rate of approximately 0.1 mm per day,^28,30^ which suggests that toenails collected at 6 weeks after birth likely represent exposures that occurred in late pregnancy and early neonatal life. Therefore, these three exposure assessments represent maternal and fetal exposures to metals that approximately occurred in periconception and early pregnancy, mid pregnancy, and late pregnancy and early neonatal life, respectively.

Toenail collection, handling, and measurement procedures used in NHBCS have been detailed elsewhere.^24,31–33^ At enrollment, women were provided materials and instructions to collect a toenail clipping from the great toe, after removing any nail polish and bathing. At approximately 2 weeks postpartum, mothers were mailed collection packets to collect another toenail clipping from themselves and also their infant within 8 weeks of birth; mothers were instructed to collect nails from all toes of their infants. Maternal toenails were collected in paper envelopes, whereas infant toenails were collected in prelabeled collection vials. Visible dirt was removed from maternal toenails and infant toenails, and maternal toenails were washed with acetone followed by deionized water and then 5 times in an ultrasonic bath using Triton X-100 (LabChem, Pittsburgh, PA). Toenails were weighed and digested in Optima nitric acid (Fisher Scientific, St. Louis, MO) by low pressure microwave digestion. After digestion, toenails were analyzed for metal concentrations using inductively coupled plasma mass spectrometry (ICP-MS) on an Agilent 7700x (Agilent Technologies, Santa Clara, CA). Quality control procedures included continuing calibration verification, digestion of fortified blanks, and inclusion of certified reference materials (National Institute for Environmental Studies Japan, #13 Hair). Analyses were performed by the Dartmouth Trace Elements Analysis Core (Hanover, NH).

This analysis includes six metals, selected for their measurement and detection in a high proportion of samples and performance quality of the laboratory measurements: As, Cu, Mn, Pb, Se, and Zn. For these six metals at the three time points, method limits of detection ranged from 2.9 × 10^−6^ µg/g to 5 µg/g (median: 0.04 µg/g), detection frequencies ranged from 71% to 100% (median: 99%), batch average recovery of standard reference materials ranged from 77% to 97% (median: 89%), and batch average recovery of spiked blanks postdigestion ranged from 96% to 104% (median: 100%). Concentrations below the method limit of detection were imputed using the limit of detection divided by 2.^34^ Further, toenail metal concentrations were log_2_-transformed.

### Behavioral outcomes

At approximately 3 years of age, caregivers were mailed two behavioral checklists for completion: the SRS-2 Preschool Form^35^ and the BASC-2 Parent Rating Scales for preschool aged children.^36^ Both instruments are appropriate for use in typically developing children as they assess dimensional traits, i.e., continuous behavioral measures that are present in the general population. Even among a nonclinical population, more extreme values for continuous BASC-2 or SRS-2 scores reflect increased disease risk.

The SRS-2 is a standardized, validated psychometric instrument designed to assess behaviors related to social impairment and autistic traits.^35^ The instrument describes 65 behaviors, which parents grade on a Likert scale (Not True, Sometimes True, Often True, Almost Always True). The SRS-2 yields a Total Score (a composite of five subscales: Social Awareness, Social Cognition, Social Communication, Social Motivation, and Restricted Interests and Repetitive Behavior), which indicates the overall severity of social deficits characteristic of the autism spectrum, with higher scores indicating more severe deficits. The SRS-2 Preschool Form is valid for children 2.5–4.5 years of age.

The BASC-2 is a standardized, validated behavioral assessment that measures a broad variety of maladaptive and adaptive behaviors.^36^ The instrument elicits information about the frequency of 134 behaviors (Never, Sometimes, Often, Almost Always). The BASC-2 yields four composite scores (Behavioral Symptoms Index [BSI], Externalizing Problems, Internalizing Problems, Adaptive Skills) derived from combinations of 12 subscales (Aggression, Anxiety, Attention Problems, Atypicality, Depression, Hyperactivity, Somatization, Withdrawal, Activities of Daily Living, Adaptability, Functional Communication, Social Skills). The BSI, Externalizing Problems, and Internalizing Problems composites reflect maladaptive behaviors and higher scores indicate more problematic behaviors; conversely, the Adaptive Skills composite reflects adaptive behaviors and higher scores indicate better behavioral outcomes. The BASC-2 includes three internal validity indices to identify assessments that were inappropriately completed, including the F-Index (implausibly negative assessments), the Response Pattern Index (assessments completed without attention to item content), and the Consistency Index (implausible inconsistencies).The BASC-2 Parent Rating Scales—Preschool Version is valid for children 2–5 years of age.

The outcomes of this analysis included the raw scores of the SRS-2 Total Score and the four BASC-2 composite scores. Raw scores were preferred to the scales’ standardized T-scores for their finer resolution and because T-scores may truncate raw scores at the tails of the distributions.^37^

### Supporting covariates

At enrollment, mothers completed questionnaires on anthropometrics, demographic and lifestyle factors, and medical history. Mothers also completed a food frequency questionnaire that was used to calculate a Healthy Eating Index score, which assesses dietary quality per the Dietary Guidelines for Americans.^38^ Data were also extracted from the prenatal medical record, and both the maternal and infant delivery records, including parity, date of birth, and sex of the child. Further, caregivers were periodically followed-up with telephone interviewers to gather information about the child’s early life, such as breastfeeding duration.

The 3-year mailing included the preschool version of the Parenting Relationship Questionnaire (PRQ-P),^39^ an assessment of parents’ perceptions of the parent–child relationship that includes 45 statements rated according to their perceived frequency (Never, Sometimes, Often, Almost Always). The PRQ-P contains five scales: Attachment, Discipline Practices, Involvement, Parenting Confidence, and Relational Frustration. Similar to the BASC-2, the PRQ includes internal validity indices to identify assessments that were inappropriately completed, including the F-Index, the Response Pattern Index, and the Consistency Index, and also the D-index (implausibly positive assessments). The PRQ-P is valid for children 2–5 years of age. In statistical analyses, principal components (PCs) of the PRQ-P scales were used to account for most variability of the scales while requiring a smaller number of variables.

Covariates were identified based on a priori evidence from the literature of their potential relationships with the exposures and/or outcomes, and included maternal age (quadratic), maternal BMI (quadratic), highest level of parental education (high school or less, any college, any graduate education), maternal self-reported lifetime smoking exposures (never first- nor second-hand, ever second-hand, ever first-hand), maternal marital status (married, other), parity (0, ≥1), child age at last breastfeeding (< 365, ≥ 365 days), maternal pregnancy 2010 Healthy Eating Index score (linear), the first three PCs of the five PRQ-P scales (linear), year of birth (2010–2011, 2012–2013, 2014–2016), sex of the child (male, female), and age of the child at testing (linear). These covariates were included in all analyses.

### Statistical analyses

Mean field variational Bayes for lagged kernel machine regression (MFVB-LKMR) was used to investigate relations between the time-varying mixtures of metals and behavioral assessment scores.^20^ MFVB-LKMR is an efficient approximation of lagged kernel machine regression (LKMR),^20,21^ a regularized kernel machine regression method for the investigation of low-resolution time-varying mixtures in relation to a time-fixed outcome.^21^ LKMR uses Markov chain Monte Carlo methods to update model parameters, which can be computationally burdensome; alternatively, MFVB-LKMR uses the mean field variational approximation method for Bayesian inference to efficiently approximate LKMR.^20^ MFVB-LKMR employs two Bayesian penalization schemes: a group lasso regularizes the exposure–response function within a time period, and a fused lasso regularizes the exposure–response function of neighboring time periods. MFVB-LKMR generates an exposure–response surface from which cross-sections may be obtained to investigate exposure–outcome relations, including nonlinear dose–response relations and interactions between metals.

A quadratic kernel was used to accommodate linear and quadratic exposure–response relations. MFVB-LKMR was performed over a maximum of 200 iterations with model parameters suggested by the method’s developers, which reportedly performed well in a variety of settings (stopping parameter = 1 × 10^−7^; hyperparameters of the group lasso: λ^2^_1_ = 60, δ_1_ = 10; hyperparameters of the fused lasso: λ^2^_2_ = 45, δ_2_ = 10).^21^ Additional detail on the model and its parameters may be found in the manuscripts that introduce the methods.^20,21^

MFVB-LKMR was used to estimate a main effect of each metal at each time point and 95% posterior credible intervals (CrI), a Bayesian analogue of a 95% confidence interval. Main effects were defined as the difference in the mean outcome predicted when an individual metal was fixed at the 75th percentile versus when that individual metal was fixed at the 25th percentile, with other metals fixed at their median values and adjusted for covariates. The bivariate dose–response relations for each metal were visually examined for nonlinearity. MFVB-LKMR was used to investigate two-way interactions (Metal_1_, Metal_2_) by estimating the difference in the mean predicted outcome at the 75th percentile of Metal_1_ versus the 25th percentile of Metal_1_ at the 75th percentile of Metal_2_, contrasted with the difference in mean outcome predicted at the 75th percentile of Metal_1_ versus the 25th percentile of Metal_1_ at the 25th percentile of Metal_2_ (other metals fixed at their medians). All metal concentrations, outcomes, and continuous covariates were standardized (µ = 0, σ^2^ = 1). These analyses were repeated stratified by sex of the child to investigate effect measure modification by sex, as sex may modify the physiological effects of metal exposure, resulting in sexually differential metal–neurodevelopment relations.^40–42^

In a sensitivity analysis to assess the impact of the omission of participants with missing and/or invalid covariate data, we imputed these covariate data using multivariate imputation by chained equations procedure via the *mice* R package,^43^ and repeated our primary analyses using a single completed data set.

In corroborating analyses, associations identified in the sex-combined MFVB-LKMR analyses were entered into parametric regression models to compare findings between the two approaches. Specifically, a parametric regression model was constructed for each outcome that included the following: any statistically significant main metal effect at α = 0.05, any statistically significant two-way metal interactions at α = 0.05 (and accompanying main effects), and covariates. In an effort to account for co-metal confounding, the first three PCs of all metals not included as main effects or interactions were additionally included; PCs were used to account for some of the variability of the excluded metals while requiring fewer variables. To improve the interpretability of these models, metal concentrations were interquartile range (IQR) transformed.

Statistical analyses were performed using R version 3.6.0 (R Foundation for Statistical Computing, Vienna, Austria). Code for MFVB-LKMR was accessed from https://github.com/shelleyhliu and adapted to the current study.

## Results

### Study population

The study sample included mother–child pairs with complete data on either behavioral assessment, the six toenail metal concentrations at the three time points, and covariate data. Additional participants were omitted from the analyses if they had invalid ages at assessment or BASC-2 or PRQ internal validity indices that were not “Acceptable.” The final analysis included 371 participants for SRS-2 assessments and 318 participants for BASC-2 assessments (Table 1; Supplemental Figure 1; http://links.lww.com/EE/A97). Of these, 306 participants were included in both the SRS-2 and BASC-2 analyses. Participants were included in the BASC-2 analysis and omitted from the SRS-2 analysis (n=12) owing to failure to complete the SRS-2 assessment (n = 9) or invalid age at SRS-2 assessment (n = 3). Similarly, some participants were included in the SRS-2 analysis and not the BASC-2 analysis owing to failure to complete the BASC-2 assessment (n = 56), partial completion of the BASC-2 assessment (n = 2), or BASC-2 internal validity indices that were not acceptable per the BASC-2 protocol (n = 7).

**Table 1.**
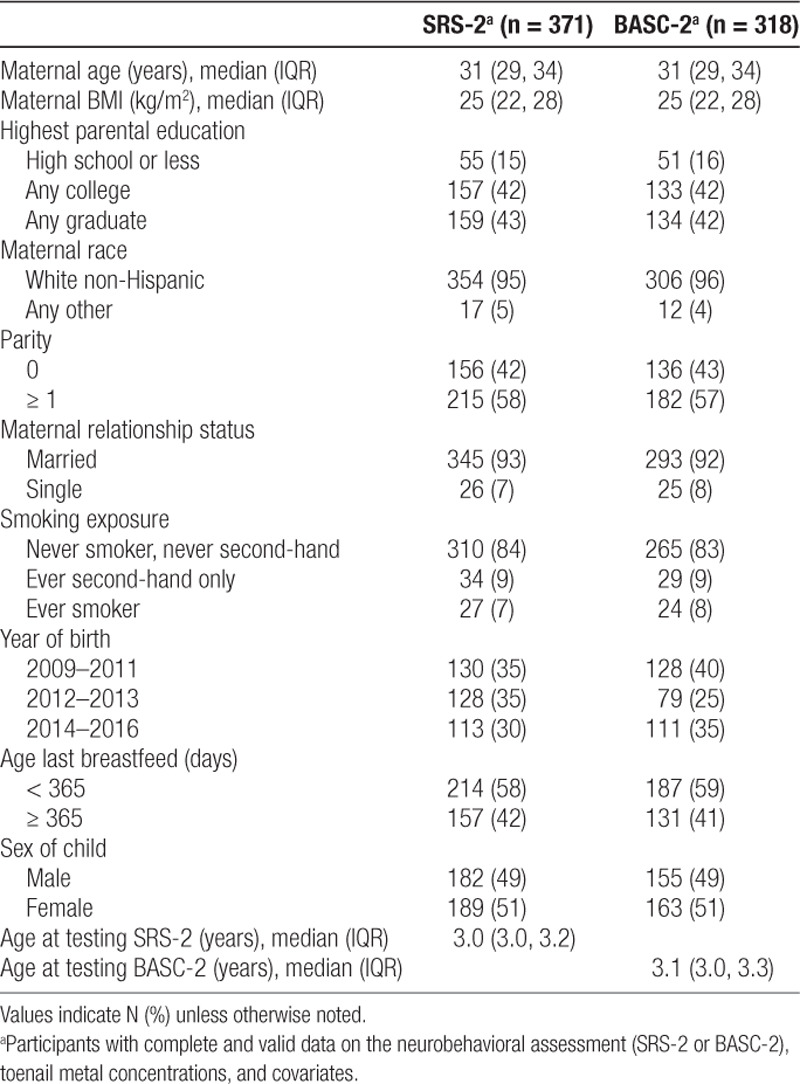
Population Characteristics

The majority of women in the study had some college education, were married, were of white non-Hispanic race/ethnicity, and reported no first- or second-hand smoking exposure. Relative to NHBCS participants who were mailed 3-year behavioral assessments but excluded from the study sample owing to missing or invalid data, mothers in the analysis sample were somewhat more likely to report a higher level of educational attainment, white non-Hispanic race/ethnicity, being married, no first- or second-hand smoking exposure, and a longer duration of breastfeeding (Supplemental Table S1; http://links.lww.com/EE/A97).

### Toenail metal concentrations

The six metals were detected in 71%–100% of samples (Table 2). In maternal toenails at both time points, median concentrations of Zn were highest, followed by Cu, Se, Mn, Pb, and As; in infant toenails, median concentrations of the metals were similarly ordered, though Mn and Se concentrations were more similar in infant toenails than in maternal toenails. Toenail metal concentrations in the analysis samples were similar to those among all eligible participants mailed neurobehavioral assessments at 3 years. Metal concentrations tended to be positively correlated (median Spearman’s ρ = 0.09), with stronger correlations occurring within time points (median Spearman’s ρ = 0.29) and among the same metal between time points (median Spearman’s ρ = 0.30) (Supplemental Table S2; http://links.lww.com/EE/A97).

**Table 2.**
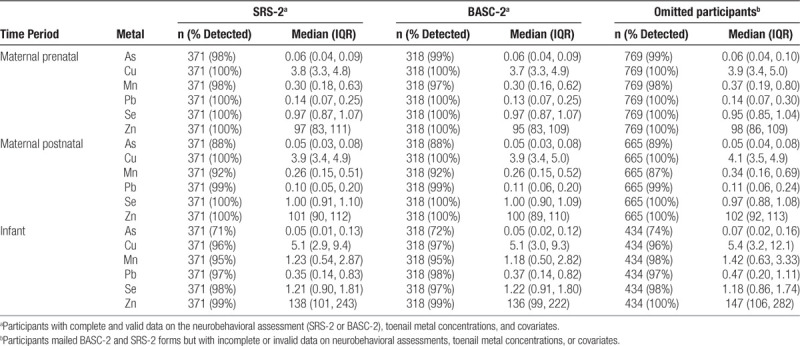
Toenail Metal Concentrations (μg/g) in SRS-2 and BASC-2 Analysis Samples and Omitted Participants

### Behavioral outcomes

Children in NHBCS had scores indicative of fewer adverse behaviors and more beneficial behaviors than the respective standardization populations of the behavioral assessments (Supplemental Table S3; http://links.lww.com/EE/A97). Children in the analysis sample performed comparably on the SRS-2 and BASC-2 to those who had 3-year assessments but were excluded owing to invalid neurobehavioral response patterns or who were missing toenail metal concentrations or covariate data (Supplemental Table S3; http://links.lww.com/EE/A97).

### Toenail metal concentrations and behavioral outcomes

In MFVB-LKMR analyses, infant toenail Mn concentrations were associated with poorer behavioral skills on the BASC-2 BSI, Externalizing Problems, and Adaptive Skills composite scores. For Mn levels at the 75th compared with the 25th percentiles, with other metals fixed at their medians, mean predicted standardized BSI scores were higher by 0.16 (95% CrI: 0.03, 0.28), Externalizing Problems scores were higher by 0.12 (95% CrI: 0.00, 0.24), and Adaptive Skills scores were reduced by 0.18 (95% CrI: −0.05 to −0.31) (Figure, Supplemental Table S4; http://links.lww.com/EE/A97). For the same comparison, maternal postnatal As toenail concentrations were associated with 0.09 (95% CrI: 0.00, 0.20) higher standardized BASC-2 BSI scores and 0.12 (95% CrI: 0.02, 0.23) higher standardized Internalizing Problems scores. Scores suggestive of better behavioral development were observed with higher toenail concentrations of Zn, including infant toenail Zn and BASC-2 BSI, Externalizing Problems, and Adaptive Skills, and with maternal postnatal toenail Zn and BASC-2 Adaptive Skills and SRS-2 Total Scores. Higher Pb in infant toenails was associated with fewer BASC-2 Externalizing Problems, and in maternal prenatal toenails with fewer BASC-2 Internalizing Problems. Weak and inconsistent associations were observed with Se and Cu. We found a tendency toward stronger associations for biomarkers of late pregnancy and early neonatal life (i.e., infant toenails), followed by biomarkers of middle pregnancy (i.e., maternal postnatal toenails) and then those of periconception and early pregnancy (i.e., maternal prenatal toenails). Associations mostly appeared linear (after log_2_ transformation), except for infant toenail Mn concentrations and BASC-2 BSI and Externalizing Problems scores, which had evidence of nonlinearity, with observations of more maladaptive behaviors largely at higher exposures (Supplemental Figure 2; http://links.lww.com/EE/A98).

**Figure.**
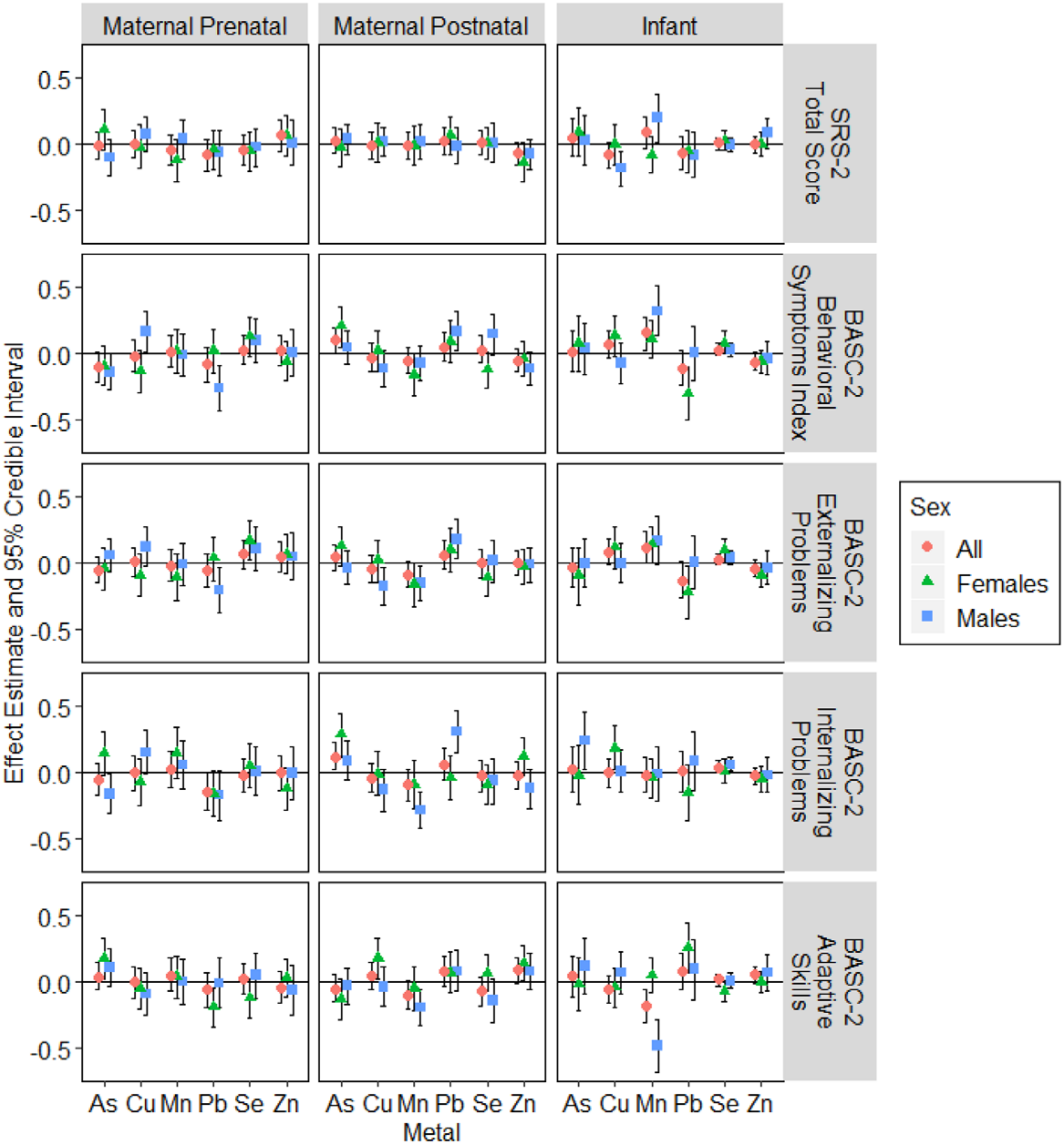
Main effect estimates and 95% credible intervals obtained from MFVB-LKMR, in sex-combined and sex-stratified analyses. Effect estimates are the difference in the mean predicted outcome (standardized) between the metal fixed at 75% vs. 25%, with all other metals fixed at their medians and adjusted for maternal age (quadratic), maternal BMI (quadratic), highest level of parental education (high school or less, any college, any graduate), sex (male, female), parity (0, ≥1), smoking status (no second- or first-hand, ever second-hand only, ever first-hand), age at last breastfeeding (< 365 days, ≥ 365 days), maternal marital status (married, other), birthyear (2010–2011, 2012–2013, 2014–2015), Healthy Eating Index (linear), Parenting Relationship Questionnaire (first three principal components), and age at assessment (linear).

MFVB-LKMR identified a two-way interaction with As and Se concentrations in infant toenails for SRS-2 Total Scores; infants with high concentrations of both metals had the highest SRS-2 Total Scores (greater social communication deficits), whereas children with high Se and low As had the lowest SRS-2 Total Scores (better social communication skills; Supplemental Figure 3; http://links.lww.com/EE/A99; Supplemental Table S5; http://links.lww.com/EE/A97). As and Se concentrations in infant toenails also appeared to interact in models of BASC-2 Adaptive Skills scores, where highest scores were observed among children with high Se and low As (Supplemental Figure 4; http://links.lww.com/EE/A99; Supplemental Table S5; http://links.lww.com/EE/A97). Zn in maternal prenatal toenails appeared to modify the relation between Pb and children’s BASC-2 Internalizing Problems scores, where at low levels of Zn, low Pb was associated with more internalizing problems scores and high Pb was associated with fewer internalizing problems scores (Supplemental Figure 5; http://links.lww.com/EE/A99; Supplemental Table S5; http://links.lww.com/EE/A97).

In sex-stratified analyses, we observed that our primary findings from the sex-combined analyses were generally consistent in males and females (Figure; Supplemental Tables S6–S9; http://links.lww.com/EE/A97). Notable exceptions were that adverse associations with infant toenail Mn tended to be stronger among males (e.g., infant toenail Mn and SRS-2 Total and BASC-2 Adaptive Skills Scores), whereas the adverse associations with maternal postnatal toenail As were often stronger among females (e.g., maternal postnatal toenail As and BASC-2 maladaptive composite scores). Interestingly, maternal postnatal toenail Mn was associated with fewer behavioral symptoms on the BASC-2 Externalizing and Internalizing symptoms among males, suggesting potential modification by exposure timing and/or levels. We observed inconsistent associations with toenail Pb when stratified by sex. However, sample sizes for these sex-stratified analyses limited our statistical precision.

In sensitivity analyses with imputed covariate data, the precision of the effect estimates increased modestly and the directionality of most associations was unchanged, though many were attenuated (Supplemental Table S10; http://links.lww.com/EE/A97). While many of the adverse associations with infant toenail Mn persisted, the adverse associations with maternal postnatal toenail As were notably attenuated. A possible explanation for these differences in associations may include differential loss-to-follow-up, where participants omitted owing to missing/invalid covariate data differed somewhat from the analysis sample, where mothers were more likely to be single and children were more likely to be older at neurobehavioral assessment, and also to have slightly higher infant toenail As concentrations.

Linear regression models were consistent with adverse associations between behavioral outcomes and As and Mn, including maternal postnatal toenail As and BASC-2 BSI scores (β = 4.25; 95% CI: 1.84, 6.66), infant toenail As and BASC-2 Adaptive Skills scores (β = −3.75; 95% CI: −6.90 to −0.61), and infant toenail Mn and BASC-2 BSI scores (β = 4.35; 95% CI: 0.04, 8.65) (Supplemental Table S11; http://links.lww.com/EE/A97). Other associations were often in the same direction as the MFVB-LKMR associations but did not achieve statistical significance.

## Discussion

In a prospective birth cohort of mother–child pairs recruited from prenatal clinics in New Hampshire, we investigated periconceptional, prenatal, and early neonatal exposures to mixtures of toxic and essential metals in relation to early childhood behavioral development. Concentrations of Mn in infant toenails and As in maternal postnatal toenails were associated with poorer behavioral development on multiple BASC-2 composite measures. Associations with Mn were typically stronger among males, while associations with As were typically stronger among females. We observed limited evidence of two-way interactions between metals. Dose–response relations appeared linear (after log_2_ transformation), with the potential exception of Mn, for which relations with certain behavioral assessments appeared nonlinear. Generally, associations between metals and behavioral outcomes were stronger with biomarkers reflecting exposures in mid pregnancy or late pregnancy and early neonatal life, relative to periconception and early pregnancy.

Our findings that Mn and As may be neurodevelopmental toxicants, particularly at higher levels of exposure, are in general concordance with available evidence.^44–48^ That these associations were typically stronger with biomarkers reflecting exposures in mid pregnancy or late pregnancy and early neonatal life relative to periconception or early pregnancy is plausible given the timing of important neurodevelopmental processes. Mid and late pregnancy are periods of rapid development of the brain.^1,49,50^ Our observation of potential modification of the metals–neurodevelopment associations by sex accords with available evidence indicating sexually differential susceptibility to metals.^40–42^ Metals may exert physiological impacts that are differential by sex through several mechanisms, including disruption of sexually differential properties of the endocrine system^40,41,51,52^ and disruption of neurotransmitters with sexually differential distributions.^42^ However, our findings regarding specific sex–metal interactions were limited and inconsistent,^42,47,53^ partially owing to decreased precision from modest sample sizes in sex-stratified analyses. In addition to these findings, we also observed associations that are not supported by the prior literature, including the lack of adverse associations of Pb. The explanation for these unexpected findings is unclear, though they may be partially attributable to residual confounding by, for example, unmeasured lifestyle factors. Additionally, such results may be owing to chance, as we did not correct for multiple testing owing to dependence among outcomes (and potentially exposures) and because this was an exploratory analysis.

To our knowledge, there is only one prior study of behavioral effects of time-varying early-life exposure to metal mixtures. Horton et al.^19^ used distributed lagged models and lagged weighted quantile sum regression to investigate relations between Mn, Zn, and Pb measured in deciduous teeth and BASC-2 scores at 8–11 of age years among children in Mexico City (n = 133). A strength of this study is that the combination of teeth biomarkers and the mixtures methods enabled investigation of prenatal and postnatal sensitive exposure periods with high resolution. The authors observed that prenatal Mn concentrations were associated with fewer adverse behavioral symptoms; conversely, postnatal Mn concentrations were associated with more anxiety symptoms, as were Zn and Pb. In our study, we observed that late pregnancy and early neonatal life Mn was associated with more adverse behaviors, and that that prenatal Zn exposures were suggestively associated with fewer adverse behaviors, and inconsistent associations with Pb. Possible explanations for the apparently discrepant findings between our study and Horton et al. include differences in the age of the children at behavioral assessment, exposure levels, study power, and control of confounding (including co-metal confounding). Nonetheless, this previous study and our own represent steps toward more holistic epidemiologic investigations of time-varying exposures to mixtures, which may provide novel insights into metal–neurodevelopment relations.

Our study used metal concentrations in toenails collected at multiple prenatal and perinatal timepoints, enabling investigation of time-varying exposures to mixtures of essential and toxic metals. Toenails are promising biomarkers of metal exposure for their noninvasive collection, easy storage and handling procedures, and capacity to reflect exposures from months prior.^24–27,54^ For As,^24,27,55^ Mn,^54–56^ Se,^54,57,58^ and Zn,^54^ toenail metal concentrations have demonstrated relations with known exposure sources and/or established biomarkers. For other metals in our study, including Cu^54^ and Pb,^59^ their validity is less well characterized. In addition to the biological relevance of metal concentrations in toenails, there is the matter of the timing of exposures reflected in toenails. Owing to natural variability in toenail growth rates and lengths, the specific time period reflected in each toenail biomarker is somewhat variable, but we do not expect this variability to bias results; more importantly, the toenail biomarkers used in our study reflect chronologically ordered exposure periods in pregnancy and early neonatal life. Additional research on the utility of toenails as biomarkers of metal exposures, including the timing of exposures reflected in toenails and particularly in pediatric populations, are needed.

To capitalize on these rich exposure data, we used MFVB-LKMR to achieve several objectives: control co-metal confounding, assess exposure–exposure interactions, assess sensitive periods of exposure, and assess nonlinear dose–response. One limitation of this method is that data were required to be complete as MFVB-LKMR does not readily accommodate missing or imputed data. This is a challenge when biomarkers are collected longitudinally, where a single missed assessment can exclude a participant from the analysis. Relatedly, we required high detection frequencies for all metal biomarkers to avoid imputing too many undetected measurements. Thus, it will be beneficial for future methods to accommodate missing and/or imputed data. Second, MFVB-LKMR may be sensitive to tuning parameters and hyperparameters, though we used parameter specifications suggested by the method’s developers that performed well in a variety of settings.^21^ Third, although an efficient approximation of LKMR, MFVB-LKMR remained computationally intensive, and thus, the development of less computationally expensive mixtures models will be important to accommodate increasingly complex exposure data.

Our study possesses other salient aspects that bear on our results. First, while NHBCS participants obtain drinking water from private wells, a potential source of elevated metals exposure, the average toenail metal concentrations in our population were comparable to other U.S. populations.^60,61^ Second, while we observed some demographic differences between our study sample and all eligible participants mailed neurobehavioral assessments at 3 years (Supplemental Table S1; http://links.lww.com/EE/A97), toenail metal concentrations (Table 2) and neurodevelopmental assessment scores (Supplemental Table S3; http://links.lww.com/EE/A97) were similar between these groups. Third, we used two neurodevelopmental instruments to assess children’s behavior: the SRS-2 and the BASC-2, which have been widely used in epidemiologic studies to characterize neurodevelopmental effects of environmental exposures. The assessments were completed at approximately 3 years, an age at which there is considerable normal behavioral variability; such large natural variability may potentially obscure small but meaningful effects of metal exposures. Understanding later life impacts of prenatal exposure will be of interest for future work. Lastly, we had rich supporting covariate data, including potential confounders and predictors of behavior. However, our study may have benefited from additional covariate data which were unavailable for this analysis. For example, income, may be related to both exposure levels and behavioral development, and parental psychological traits, can be strong predictors of children’s neurobehavioral development. Additionally, confounding by maternal seafood consumption is possible, as seafood contains both essential nutrients and certain metals (e.g., Hg) that may be neurodevelopmentally significant.^62,63^ We anticipate that the inclusion of additional covariates that control confounding and/or predict the behavioral outcomes would likely have increased the precision of our effect estimates; however, it is unclear whether the net uncontrolled bias owing to confounding for each effect estimate is likely to be toward or away from the null hypothesis of no effect.

In conclusion, our study contributes evidence regarding potential neurodevelopmental effects of prenatal exposures to essential and toxic metals and may help to inform intervention and policy efforts, as well as future research, regarding environmental exposure to metals among our most vulnerable populations.

## Conflict of interest statement

The authors declare that they have no conflicts of interest with regard to the content of this report.

The specific aims of the study were supported by P01ES022832 from National Institute of Environmental Health Sciences, RD83544201 from United States Environmental Protection Agency, and P20GM104416 from National Institute of General Medical Sciences. The Trace Element Analysis Facility is supported in part by P42ES007373 from National Institute of Environmental Health Sciences and by 5P30CA023108-37 from National Cancer Institute. B.T.D. was supported by R25CA134286 from National Cancer Institute.

Use of the data may be possible under certain conditions by contacting the New Hampshire Birth Cohort Study Principal Investigator: Margaret Karagas, PhD, (margaret.r.karagas@dartmouth.edu).

## Supplementary Material


