# Supplementary material for: Periconceptional and prenatal exposure to metal mixtures in relation to behavioral development at 3 years of age
Source: Environ Epidemiol. 2020 Jul 6;4(4):e0106. doi: 10.1097/EE9.0000000000000106 (PMC7595192; doi:10.1097/EE9.0000000000000106)
Supplement: Supplementary file 1 [file ee9-4-e0106-s001.docx]

**Supplemental Material**

**Periconceptional and prenatal exposure to metal mixtures in relation to behavioral development at three years of age**

Brett T. Doherty, Megan E. Romano, Jiang Gui, Tracy Punshon, Brian Jackson, Margaret R. Karagas, Susan Korrick

Table of Contents

Supplemental Figure 1. Flow chart contextualizing analysis sample…………………………………………………2

Supplemental Figure 3. Exposure-response surface of predicted SRS-2 Total Scores as a function of infant toenail concentrations of As and Se……………………………………………………………………………………..3

Supplemental Figure 4. Exposure-response surface of predicted BASC-2 Adaptive Skills Scores as a function of infant toenail concentrations of As and Se…………………………………………………………………………...4

Supplemental Figure 5. Exposure-response surface of predicted BASC-2 Internalizing Problems Scores as a function of maternal prenatal toenail concentrations of As and Se…………………………………………………...5

Supplemental Table S1. Population characteristics in analysis samples and omitted participants……………….6

Supplemental Table S2. Spearman correlation coefficients among the six metals measured at three time points in the analysis sample (BASC-2 and/or SRS-2, n = 383)……………………………………………………………...7

Supplemental Table S3. Behavioral assessment scores among the study samples and omitted participants….8

Supplemental Table S4. Main effects of each metal at each time point……………………………………………..9

Supplemental Table S5. Interactions between metals within time points………………………………………..…10

Supplemental Table S6. Main effects of each metal at each time point, among male children………………….21

Supplemental Table S7. Interactions between metals within time points, among male children………………..23

Supplemental Table S8. Main effects of each metal at each time point, among female children……………….33

Supplemental Table S9. Interactions between metals within time points, among female children…………..….36

Supplemental Table S10. Main effects of each metal at each time point, with imputed covariate data (SRS-2: n = 477; BASC-2: n = 410)…………………………………………………………………………………………………46

Supplemental Table S11. Linear models containing metal biomarkers and their interactions identified as statistically significant at α = 0.05 in MFVB-LKMR analyses………………………………………………………...49


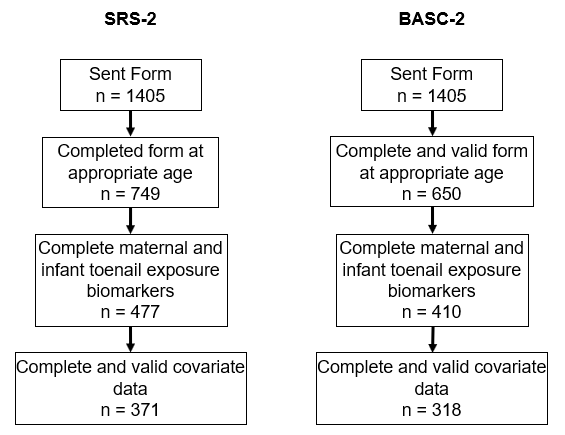


Supplemental Figure 1. Flow chart contextualizing analysis sample.


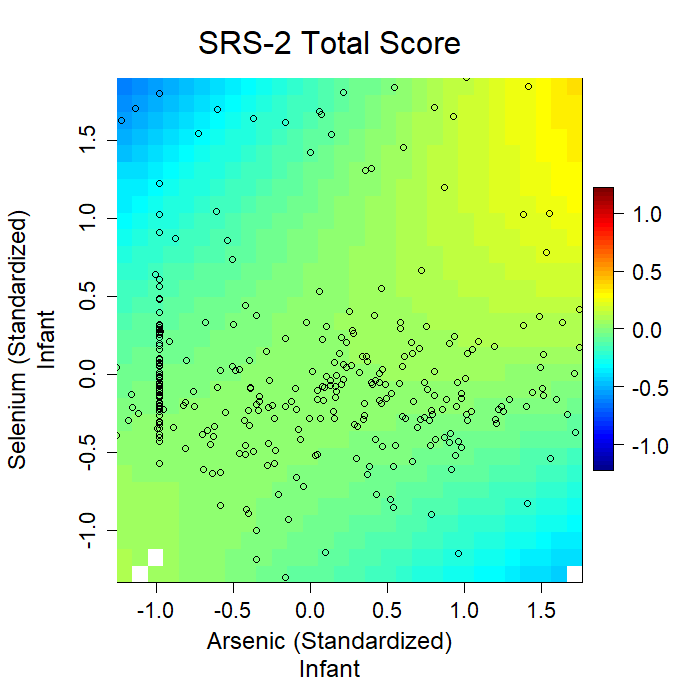


Supplemental Figure 3. Exposure-response surface of predicted SRS-2 Total Scores as a function of infant toenail concentrations of As and Se. Other metals fixed at their medians and adjusted for maternal age (quadratic), maternal BMI (quadratic), highest level of parental education (high school or less, any college, any graduate), sex (male, female), parity (0, ≥1), smoking status (no second- or first-hand, ever second-hand only, ever first-hand), age at last breastfeeding (<365 days, ≥365 days), maternal marital status (married, other), birth year (2010-2011, 2012-2013, 2014-2015), Healthy Eating Index (linear), Parenting Relationship Questionnaire (first three principal components), and age at assessment (linear).


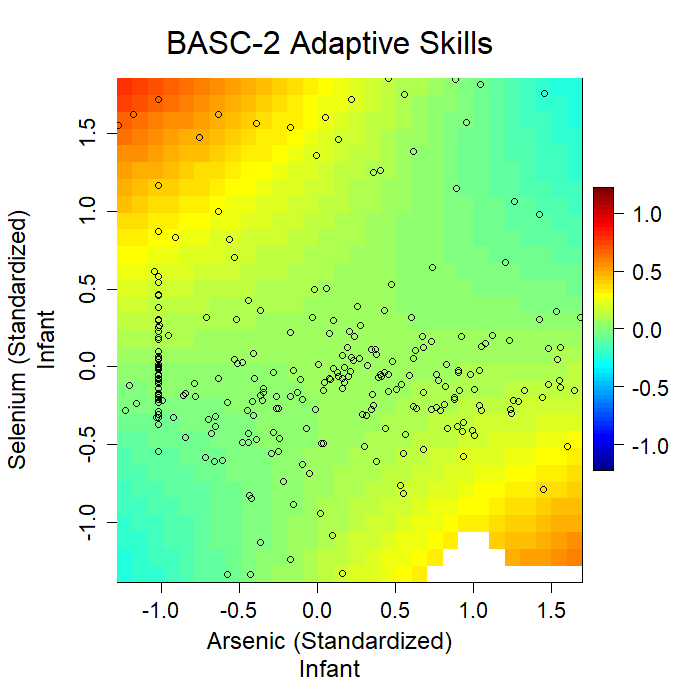


Supplemental Figure 4. Exposure-response surface of predicted BASC-2 Adaptive Skills Scores as a function of infant toenail concentrations of As and Se. Other metals fixed at their medians and adjusted for maternal age (quadratic), maternal BMI (quadratic), highest level of parental education (high school or less, any college, any graduate), sex (male, female), parity (0, ≥1), smoking status (no second- or first-hand, ever second-hand only, ever first-hand), age at last breastfeeding (<365 days, ≥365 days), maternal marital status (married, other), birth year (2010-2011, 2012-2013, 2014-2015), Healthy Eating Index (linear), Parenting Relationship Questionnaire (first three principal components), and age at assessment (linear).


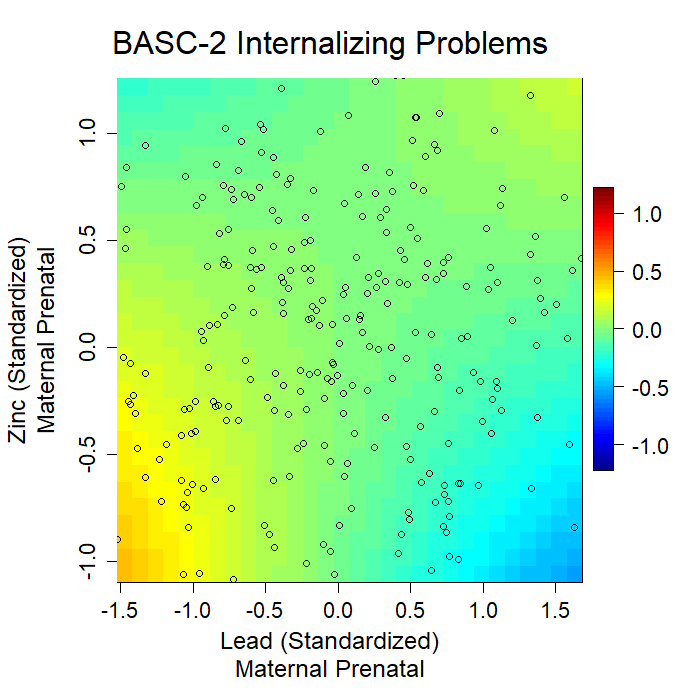


Supplemental Figure 5. Exposure-response surface of predicted BASC-2 Internalizing Problems Scores as a function of maternal prenatal toenail concentrations of As and Se. Other metals fixed at their medians and adjusted for maternal age (quadratic), maternal BMI (quadratic), highest level of parental education (high school or less, any college, any graduate), sex (male, female), parity (0, ≥1), smoking status (no second- or first-hand, ever second-hand only, ever first-hand), age at last breastfeeding (<365 days, ≥365 days), maternal marital status (married, other), birth year (2010-2011, 2012-2013, 2014-2015), Healthy Eating Index (linear), Parenting Relationship Questionnaire (first three principal components), and age at assessment (linear).

| Supplemental Table S1. Population characteristics in analysis samples and omitted participants. | | | | |
| --- | --- | --- | --- | --- |
|  |  | Omitted Participants^a^ (n=1022) | SRS-2^b^ (n=371) | BASC-2^b^ (n=318) |
| Maternal Age (years) | Median (IQR) | 31 (28, 35) | 31 (29, 34) | 31 (29, 34) |
|  | Missing | 0 | 0 | 0 |
| Maternal BMI (kg/m^2^) | Median (IQR) | 24 (22, 29) | 25 (22, 28) | 25 (22, 28) |
|  | Missing | 46 | 0 | 0 |
| Highest Parental Education | High School or Less | 237 (29) | 55 (15) | 51 (16) |
|  | Any College | 308 (38) | 157 (42) | 133 (42) |
|  | Any Graduate | 270 (33) | 159 (43) | 134 (42) |
|  | Missing | 207 | 0 | 0 |
| Maternal Race | White Non-Hispanic | 938 (92) | 354 (95) | 306 (96) |
|  | Any Other | 84 (8) | 17 (5) | 12 (4) |
|  | Missing | 0 | 0 | 0 |
| Parity | 0 | 408 (41) | 156 (42) | 136 (43) |
|  | ≥1 | 592 (59) | 215 (58) | 182 (57) |
|  | Missing | 22 | 0 | 0 |
| Maternal Relationship Status | Married | 721 (84) | 345 (93) | 293 (92) |
|  | Single | 135 (16) | 26 (7) | 25 (8) |
|  | Missing | 166 | 0 | 0 |
| Smoking Exposure | Never Smoker or Second Hand | 640 (75) | 310 (84) | 265 (83) |
|  | Ever Second Hand Only | 66 (8) | 34 (9) | 29 (9) |
|  | Ever Smoker | 144 (17) | 27 (7) | 24 (8) |
|  | Missing | 172 | 0 | 0 |
| Year of Birth | 2009 to 2011 | 312 (31) | 130 (35) | 128 (40) |
|  | 2012 to 2013 | 372 (36) | 128 (35) | 79 (25) |
|  | 2014 to 2016 | 338 (33) | 113 (30) | 111 (35) |
|  | Missing | 0 | 0 | 0 |
| Age Last Breastfeed (Days) | <365 | 508 (71) | 214 (58) | 187 (59) |
|  | ≥365 | 212 (29) | 157 (42) | 131 (41) |
|  | Missing | 302 | 0 | 0 |
| Sex of Child | Male | 497 (49) | 182 (49) | 155 (49) |
|  | Female | 525 (51) | 189 (51) | 163 (51) |
|  | Missing | 0 | 0 | 0 |
| Age at Testing SRS-2 (years) | Median (IQR) | 3.1 (3.0, 3.6) | 3.0 (3.0, 3.2) |  |
|  | Missing | 632 | 0 |  |
| Age at Testing BASC-2 (years) | Median (IQR) | 3.1 (3.0, 3.7) |  | 3.1 (3.0, 3.3) |
|  | Missing | 678 |  | 0 |
| Values indicate N (%) unless otherwise noted.  ^a^Participants mailed BASC-2 and SRS-2 forms but with incomplete or invalid data on neurobehavioral assessments, toenail metal concentrations, or covariates. ^b^Participants with complete and valid data on the neurobehavioral assessment (SRS-2 or BASC-2), toenail metal concentrations, and covariates.  Abbreviations: BASC-2, Behavioral Assessment System for Children, 2^nd^ Ed.; IQR, Interquartile Range; SRS-2, Social Responsiveness Scale, 2^nd^ Ed. | | | | |

| Supplemental Table S2. Spearman correlation coefficients among the six metals measured at three time points in the analysis sample (BASC-2 and/or SRS-2, n = 383). | | | | | | | | | | | | | | | | | | | |
| --- | --- | --- | --- | --- | --- | --- | --- | --- | --- | --- | --- | --- | --- | --- | --- | --- | --- | --- | --- |
|  |  | Maternal Prenatal | | | | | | Maternal Postnatal | | | | | | Infant | | | | | |
|  |  | As | Cu | Mn | Pb | Se | Zn | As | Cu | Mn | Pb | Se | Zn | As | Cu | Mn | Pb | Se | Zn |
| Maternal Prenatal | As | 1.00 | 0.09 | 0.31 | 0.26 | 0.01 | -0.02 | 0.63 | 0.01 | 0.21 | 0.16 | -0.06 | -0.01 | 0.21 | -0.01 | -0.03 | 0.07 | -0.04 | 0.06 |
|  | Cu | 0.09 | 1.00 | 0.14 | 0.32 | 0.24 | 0.20 | -0.01 | 0.63 | 0.01 | 0.16 | 0.09 | 0.07 | -0.02 | 0.34 | 0.05 | 0.14 | 0.08 | 0.12 |
|  | Mn | 0.31 | 0.14 | 1.00 | 0.46 | 0.08 | 0.24 | 0.16 | 0.01 | 0.55 | 0.30 | -0.08 | 0.07 | 0.02 | 0.10 | 0.23 | 0.20 | -0.04 | 0.06 |
|  | Pb | 0.26 | 0.32 | 0.46 | 1.00 | 0.10 | 0.15 | 0.14 | 0.20 | 0.26 | 0.58 | -0.02 | 0.05 | 0.04 | 0.22 | 0.20 | 0.32 | -0.01 | 0.05 |
|  | Se | 0.01 | 0.24 | 0.08 | 0.10 | 1.00 | 0.37 | -0.01 | 0.05 | -0.05 | 0.00 | 0.55 | 0.16 | -0.04 | 0.11 | 0.05 | 0.04 | 0.09 | 0.09 |
|  | Zn | -0.02 | 0.20 | 0.24 | 0.15 | 0.37 | 1.00 | -0.07 | -0.04 | 0.00 | 0.06 | 0.08 | 0.49 | -0.02 | 0.03 | 0.15 | 0.12 | 0.06 | 0.18 |
| Maternal Postnatal | As | 0.63 | -0.01 | 0.16 | 0.14 | -0.01 | -0.07 | 1.00 | 0.12 | 0.33 | 0.19 | -0.08 | -0.04 | 0.29 | -0.02 | 0.05 | 0.02 | -0.05 | 0.01 |
|  | Cu | 0.01 | 0.63 | 0.01 | 0.20 | 0.05 | -0.04 | 0.12 | 1.00 | 0.11 | 0.27 | 0.18 | 0.16 | -0.08 | 0.22 | -0.03 | 0.05 | 0.04 | 0.01 |
|  | Mn | 0.21 | 0.01 | 0.55 | 0.26 | -0.05 | 0.00 | 0.33 | 0.11 | 1.00 | 0.39 | -0.09 | 0.07 | 0.08 | 0.01 | 0.26 | 0.13 | -0.06 | -0.02 |
|  | Pb | 0.16 | 0.16 | 0.30 | 0.58 | 0.00 | 0.06 | 0.19 | 0.27 | 0.39 | 1.00 | -0.03 | 0.06 | 0.03 | 0.13 | 0.13 | 0.36 | -0.21 | 0.00 |
|  | Se | -0.06 | 0.09 | -0.08 | -0.02 | 0.55 | 0.08 | -0.08 | 0.18 | -0.09 | -0.03 | 1.00 | 0.38 | -0.16 | -0.01 | -0.11 | -0.09 | 0.09 | -0.12 |
|  | Zn | -0.01 | 0.07 | 0.07 | 0.05 | 0.16 | 0.49 | -0.04 | 0.16 | 0.07 | 0.06 | 0.38 | 1.00 | -0.12 | -0.12 | -0.01 | -0.10 | 0.01 | -0.02 |
| Infant | As | 0.21 | -0.02 | 0.02 | 0.04 | -0.04 | -0.02 | 0.29 | -0.08 | 0.08 | 0.03 | -0.16 | -0.12 | 1.00 | 0.34 | 0.45 | 0.48 | 0.13 | 0.21 |
|  | Cu | -0.01 | 0.34 | 0.10 | 0.22 | 0.11 | 0.03 | -0.02 | 0.22 | 0.01 | 0.13 | -0.01 | -0.12 | 0.34 | 1.00 | 0.42 | 0.60 | 0.36 | 0.38 |
|  | Mn | -0.03 | 0.05 | 0.23 | 0.20 | 0.05 | 0.15 | 0.05 | -0.03 | 0.26 | 0.13 | -0.11 | -0.01 | 0.45 | 0.42 | 1.00 | 0.64 | 0.20 | 0.41 |
|  | Pb | 0.07 | 0.14 | 0.20 | 0.32 | 0.04 | 0.12 | 0.02 | 0.05 | 0.13 | 0.36 | -0.09 | -0.10 | 0.48 | 0.60 | 0.64 | 1.00 | 0.19 | 0.37 |
|  | Se | -0.04 | 0.08 | -0.04 | -0.01 | 0.09 | 0.06 | -0.05 | 0.04 | -0.06 | -0.21 | 0.09 | 0.01 | 0.13 | 0.36 | 0.20 | 0.19 | 1.00 | 0.34 |
|  | Zn | 0.06 | 0.12 | 0.06 | 0.05 | 0.09 | 0.18 | 0.01 | 0.01 | -0.02 | 0.00 | -0.12 | -0.02 | 0.21 | 0.38 | 0.41 | 0.37 | 0.34 | 1.00 |
| Abbreviations: BASC-2, Behavioral Assessment System for Children, 2^nd^ Ed.; SRS-2, Social Responsiveness Scale, 2^nd^ Ed. | | | | | | | | | | | | | | | | | | | |

| Supplemental Table S3. Behavioral assessment scores among the study samples and omitted participants. | | | | |
| --- | --- | --- | --- | --- |
|  |  | Omitted Participants^a^ | SRS-2 Analysis Sample^b^ | BASC-2 Analysis Sample^b^ |
| SRS-2 Total Score | n | 388 | 371 |  |
|  | Raw Score | 25 (16, 35) | 24 (18, 33) |  |
|  | T-Score | 44 (40, 47) | 43 (41, 47) |  |
| BASC-2 Behavioral Symptoms Index | n | 345 |  | 318 |
|  | Raw Score | 280 (260, 304) |  | 286 (261, 303) |
|  | T-Score | 45 (41, 51) |  | 47 (41, 51) |
| BASC-2 Externalizing Problems | n | 346 |  | 318 |
|  | Raw Score | 93 (83, 102) |  | 94 (86, 103) |
|  | T-Score | 46 (41, 51) |  | 47 (42, 52) |
| BASC-2 Internalizing Problems | n | 349 |  | 318 |
|  | Raw Score | 144 (130, 158) |  | 146 (133, 159) |
|  | T-Score | 47 (41, 53) |  | 48 (43, 54) |
| BASC-2 Adaptive Skills | n | 350 |  | 318 |
|  | Raw Score | 217 (201, 233) |  | 216 (202, 230) |
|  | T-Score | 55 (50, 61) |  | 55 (51, 60) |
| Note: All values are median (IQR). SRS-2 and BASC-2 T-scores in their respective standardization populations are distributed with a mean of 50 and standard deviation of 10.  ^a^Participants who completed BASC-2 or SRS-2 forms but had invalid neurobehavioral response patterns or who were missing toenail metal concentrations or covariate data. ^b^Participants with complete and valid data on the neurobehavioral assessment (SRS-2 or BASC-2), toenail metal concentrations, and covariates.  Abbreviations: BASC-2, Behavioral Assessment System for Children, 2nd Ed.; IQR, Interquartile Range; SRS-2, Social Responsiveness Scale, 2nd Ed. | | | | |

| Supplemental Table S4. Main effects of each metal at each time point. | | | | | | |
| --- | --- | --- | --- | --- | --- | --- |
| Outcome | Time | Metal | Estimate | SD | 95% CrI Low | 95% CrI High |
| SRS-2 Total | Maternal Prenatal | As | -0.01 | 0.05 | -0.11 | 0.10 |
| SRS-2 Total | Maternal Prenatal | Cu | 0.00 | 0.05 | -0.10 | 0.11 |
| SRS-2 Total | Maternal Prenatal | Mn | -0.04 | 0.06 | -0.16 | 0.07 |
| SRS-2 Total | Maternal Prenatal | Pb | -0.08 | 0.06 | -0.20 | 0.04 |
| SRS-2 Total | Maternal Prenatal | Se | -0.04 | 0.06 | -0.15 | 0.07 |
| SRS-2 Total | Maternal Prenatal | Zn | 0.07 | 0.06 | -0.05 | 0.19 |
| SRS-2 Total | Maternal Postnatal | As | 0.03 | 0.05 | -0.07 | 0.13 |
| SRS-2 Total | Maternal Postnatal | Cu | -0.01 | 0.05 | -0.11 | 0.09 |
| SRS-2 Total | Maternal Postnatal | Mn | -0.01 | 0.05 | -0.11 | 0.09 |
| SRS-2 Total | Maternal Postnatal | Pb | 0.03 | 0.05 | -0.08 | 0.13 |
| SRS-2 Total | Maternal Postnatal | Se | 0.01 | 0.05 | -0.08 | 0.11 |
| SRS-2 Total | Maternal Postnatal | Zn | -0.07 | 0.05 | -0.16 | 0.02 |
| SRS-2 Total | Infant | As | 0.05 | 0.07 | -0.09 | 0.20 |
| SRS-2 Total | Infant | Cu | -0.08 | 0.05 | -0.18 | 0.01 |
| SRS-2 Total | Infant | Mn | 0.09 | 0.06 | -0.03 | 0.21 |
| SRS-2 Total | Infant | Pb | -0.06 | 0.06 | -0.19 | 0.06 |
| SRS-2 Total | Infant | Se | 0.01 | 0.02 | -0.04 | 0.05 |
| SRS-2 Total | Infant | Zn | 0.00 | 0.03 | -0.07 | 0.06 |
| BASC-2 BSI | Maternal Prenatal | As | -0.10 | 0.05 | -0.21 | 0.01 |
| BASC-2 BSI | Maternal Prenatal | Cu | -0.02 | 0.06 | -0.13 | 0.10 |
| BASC-2 BSI | Maternal Prenatal | Mn | 0.02 | 0.06 | -0.10 | 0.14 |
| BASC-2 BSI | Maternal Prenatal | Pb | -0.08 | 0.06 | -0.21 | 0.05 |
| BASC-2 BSI | Maternal Prenatal | Se | 0.03 | 0.06 | -0.08 | 0.14 |
| BASC-2 BSI | Maternal Prenatal | Zn | 0.03 | 0.06 | -0.09 | 0.14 |
| BASC-2 BSI | Maternal Postnatal | As | 0.10 | 0.05 | 0.00 | 0.20 |
| BASC-2 BSI | Maternal Postnatal | Cu | -0.03 | 0.05 | -0.13 | 0.08 |
| BASC-2 BSI | Maternal Postnatal | Mn | -0.05 | 0.05 | -0.15 | 0.05 |
| BASC-2 BSI | Maternal Postnatal | Pb | 0.05 | 0.05 | -0.05 | 0.16 |
| BASC-2 BSI | Maternal Postnatal | Se | 0.03 | 0.05 | -0.07 | 0.14 |
| BASC-2 BSI | Maternal Postnatal | Zn | -0.05 | 0.04 | -0.13 | 0.04 |
| BASC-2 BSI | Infant | As | 0.02 | 0.08 | -0.13 | 0.17 |
| BASC-2 BSI | Infant | Cu | 0.07 | 0.05 | -0.03 | 0.17 |
| BASC-2 BSI | Infant | Mn | 0.16 | 0.06 | 0.03 | 0.28 |
| BASC-2 BSI | Infant | Pb | -0.11 | 0.07 | -0.24 | 0.03 |
| BASC-2 BSI | Infant | Se | 0.03 | 0.02 | -0.01 | 0.08 |
| BASC-2 BSI | Infant | Zn | -0.06 | 0.03 | -0.12 | 0.01 |
| BASC-2 EXT | Maternal Prenatal | As | -0.05 | 0.05 | -0.15 | 0.05 |
| BASC-2 EXT | Maternal Prenatal | Cu | 0.01 | 0.06 | -0.10 | 0.12 |
| BASC-2 EXT | Maternal Prenatal | Mn | -0.02 | 0.06 | -0.13 | 0.10 |
| BASC-2 EXT | Maternal Prenatal | Pb | -0.05 | 0.06 | -0.18 | 0.07 |
| BASC-2 EXT | Maternal Prenatal | Se | 0.07 | 0.05 | -0.04 | 0.17 |
| BASC-2 EXT | Maternal Prenatal | Zn | 0.05 | 0.06 | -0.06 | 0.16 |
| BASC-2 EXT | Maternal Postnatal | As | 0.05 | 0.05 | -0.05 | 0.14 |
| BASC-2 EXT | Maternal Postnatal | Cu | -0.04 | 0.05 | -0.14 | 0.06 |
| BASC-2 EXT | Maternal Postnatal | Mn | -0.09 | 0.05 | -0.18 | 0.01 |
| BASC-2 EXT | Maternal Postnatal | Pb | 0.06 | 0.05 | -0.04 | 0.17 |
| BASC-2 EXT | Maternal Postnatal | Se | 0.00 | 0.05 | -0.11 | 0.10 |
| BASC-2 EXT | Maternal Postnatal | Zn | 0.00 | 0.05 | -0.09 | 0.09 |
| BASC-2 EXT | Infant | As | -0.03 | 0.07 | -0.18 | 0.12 |
| BASC-2 EXT | Infant | Cu | 0.08 | 0.05 | -0.01 | 0.18 |
| BASC-2 EXT | Infant | Mn | 0.12 | 0.06 | 0.00 | 0.24 |
| BASC-2 EXT | Infant | Pb | -0.13 | 0.07 | -0.26 | 0.01 |
| BASC-2 EXT | Infant | Se | 0.03 | 0.02 | -0.01 | 0.07 |
| BASC-2 EXT | Infant | Zn | -0.04 | 0.03 | -0.10 | 0.02 |
| BASC-2 INZ | Maternal Prenatal | As | -0.05 | 0.06 | -0.17 | 0.07 |
| BASC-2 INZ | Maternal Prenatal | Cu | 0.00 | 0.07 | -0.13 | 0.13 |
| BASC-2 INZ | Maternal Prenatal | Mn | 0.02 | 0.07 | -0.11 | 0.16 |
| BASC-2 INZ | Maternal Prenatal | Pb | -0.14 | 0.07 | -0.28 | 0.00 |
| BASC-2 INZ | Maternal Prenatal | Se | -0.02 | 0.06 | -0.14 | 0.10 |
| BASC-2 INZ | Maternal Prenatal | Zn | 0.00 | 0.07 | -0.13 | 0.13 |
| BASC-2 INZ | Maternal Postnatal | As | 0.12 | 0.05 | 0.02 | 0.23 |
| BASC-2 INZ | Maternal Postnatal | Cu | -0.04 | 0.06 | -0.15 | 0.07 |
| BASC-2 INZ | Maternal Postnatal | Mn | -0.09 | 0.06 | -0.21 | 0.02 |
| BASC-2 INZ | Maternal Postnatal | Pb | 0.06 | 0.06 | -0.05 | 0.18 |
| BASC-2 INZ | Maternal Postnatal | Se | -0.02 | 0.06 | -0.14 | 0.09 |
| BASC-2 INZ | Maternal Postnatal | Zn | -0.02 | 0.05 | -0.12 | 0.08 |
| BASC-2 INZ | Infant | As | 0.03 | 0.08 | -0.14 | 0.19 |
| BASC-2 INZ | Infant | Cu | 0.00 | 0.06 | -0.11 | 0.11 |
| BASC-2 INZ | Infant | Mn | -0.02 | 0.07 | -0.15 | 0.12 |
| BASC-2 INZ | Infant | Pb | 0.01 | 0.08 | -0.14 | 0.16 |
| BASC-2 INZ | Infant | Se | 0.04 | 0.02 | 0.00 | 0.09 |
| BASC-2 INZ | Infant | Zn | -0.02 | 0.04 | -0.09 | 0.04 |
| BASC-2 AKL | Maternal Prenatal | As | 0.04 | 0.06 | -0.06 | 0.15 |
| BASC-2 AKL | Maternal Prenatal | Cu | 0.00 | 0.06 | -0.12 | 0.11 |
| BASC-2 AKL | Maternal Prenatal | Mn | 0.05 | 0.06 | -0.07 | 0.18 |
| BASC-2 AKL | Maternal Prenatal | Pb | -0.06 | 0.07 | -0.19 | 0.07 |
| BASC-2 AKL | Maternal Prenatal | Se | 0.02 | 0.06 | -0.09 | 0.14 |
| BASC-2 AKL | Maternal Prenatal | Zn | -0.04 | 0.06 | -0.16 | 0.08 |
| BASC-2 AKL | Maternal Postnatal | As | -0.05 | 0.05 | -0.15 | 0.06 |
| BASC-2 AKL | Maternal Postnatal | Cu | 0.05 | 0.05 | -0.06 | 0.15 |
| BASC-2 AKL | Maternal Postnatal | Mn | -0.10 | 0.05 | -0.20 | 0.01 |
| BASC-2 AKL | Maternal Postnatal | Pb | 0.08 | 0.06 | -0.03 | 0.19 |
| BASC-2 AKL | Maternal Postnatal | Se | -0.07 | 0.06 | -0.18 | 0.04 |
| BASC-2 AKL | Maternal Postnatal | Zn | 0.09 | 0.05 | -0.01 | 0.18 |
| BASC-2 AKL | Infant | As | 0.05 | 0.08 | -0.11 | 0.20 |
| BASC-2 AKL | Infant | Cu | -0.05 | 0.05 | -0.16 | 0.05 |
| BASC-2 AKL | Infant | Mn | -0.18 | 0.07 | -0.31 | -0.05 |
| BASC-2 AKL | Infant | Pb | 0.08 | 0.07 | -0.06 | 0.22 |
| BASC-2 AKL | Infant | Se | 0.02 | 0.02 | -0.03 | 0.06 |
| BASC-2 AKL | Infant | Zn | 0.06 | 0.03 | -0.01 | 0.12 |
| Estimate is the difference in the mean predicted outcome (standardized) between the metal fixed at 75% versus 25%, with all other metals fixed at their medians.  Models adjusted for maternal age (quadratic), maternal BMI (quadratic), highest level of parental education (high school or less, any college, any graduate), sex (male, female), parity (0, ≥1), smoking status (no second- or first-hand, ever second-hand only, ever first-hand), age at last breastfeeding (<365 days, ≥365 days), maternal marital status (married, other), birthyear (2010-2011, 2012-2013, 2014-2015), Healthy Eating Index (linear), Parenting Relationship Questionnaire (first three principal components), and age at assessment (linear).  Abbreviations: AKL, Adaptive Skills; BASC-2, Behavioral Assessment System for Children, 2^nd^ Ed.; BSI, Behavioral Symptoms Index; CrI, Credible Interval; EXT, Externalizing Problems; INZ, Internalizing Problems; SRS-2, Social Responsiveness Scale, 2^nd^ Ed. | | | | | | |

| Supplemental Table S5. Interactions between metals within time points. | | | | | | | |  |
| --- | --- | --- | --- | --- | --- | --- | --- | --- |
| Outcome | Time | Metal 1 | Metal 2 | Estimate | SD | 95% CrI Low | 95% CrI High | |
| SRS-2 Total | Maternal Prenatal | As | Cu | 0.00 | 0.08 | -0.16 | 0.16 | |
| SRS-2 Total | Maternal Postnatal | As | Cu | -0.02 | 0.07 | -0.16 | 0.12 | |
| SRS-2 Total | Infant | As | Cu | 0.02 | 0.12 | -0.21 | 0.25 | |
| SRS-2 Total | Maternal Prenatal | As | Mn | 0.07 | 0.09 | -0.10 | 0.24 | |
| SRS-2 Total | Maternal Postnatal | As | Mn | 0.08 | 0.08 | -0.07 | 0.24 | |
| SRS-2 Total | Infant | As | Mn | -0.01 | 0.12 | -0.25 | 0.22 | |
| SRS-2 Total | Maternal Prenatal | As | Pb | 0.12 | 0.09 | -0.06 | 0.29 | |
| SRS-2 Total | Maternal Postnatal | As | Pb | -0.04 | 0.08 | -0.19 | 0.12 | |
| SRS-2 Total | Infant | As | Pb | -0.04 | 0.13 | -0.30 | 0.22 | |
| SRS-2 Total | Maternal Prenatal | As | Se | -0.02 | 0.09 | -0.19 | 0.15 | |
| SRS-2 Total | Maternal Postnatal | As | Se | 0.00 | 0.07 | -0.14 | 0.15 | |
| SRS-2 Total | Infant | As | Se | 0.15 | 0.11 | -0.07 | 0.37 | |
| SRS-2 Total | Maternal Prenatal | As | Zn | 0.01 | 0.09 | -0.16 | 0.18 | |
| SRS-2 Total | Maternal Postnatal | As | Zn | -0.05 | 0.08 | -0.19 | 0.10 | |
| SRS-2 Total | Infant | As | Zn | -0.12 | 0.12 | -0.35 | 0.11 | |
| SRS-2 Total | Maternal Prenatal | Cu | As | 0.00 | 0.08 | -0.16 | 0.16 | |
| SRS-2 Total | Maternal Postnatal | Cu | As | -0.02 | 0.07 | -0.17 | 0.12 | |
| SRS-2 Total | Infant | Cu | As | 0.02 | 0.09 | -0.14 | 0.19 | |
| SRS-2 Total | Maternal Prenatal | Cu | Mn | -0.05 | 0.08 | -0.21 | 0.11 | |
| SRS-2 Total | Maternal Postnatal | Cu | Mn | -0.02 | 0.08 | -0.18 | 0.13 | |
| SRS-2 Total | Infant | Cu | Mn | 0.09 | 0.08 | -0.07 | 0.25 | |
| SRS-2 Total | Maternal Prenatal | Cu | Pb | 0.08 | 0.09 | -0.09 | 0.25 | |
| SRS-2 Total | Maternal Postnatal | Cu | Pb | 0.04 | 0.08 | -0.11 | 0.19 | |
| SRS-2 Total | Infant | Cu | Pb | -0.16 | 0.08 | -0.32 | 0.00 | |
| SRS-2 Total | Maternal Prenatal | Cu | Se | 0.02 | 0.08 | -0.15 | 0.18 | |
| SRS-2 Total | Maternal Postnatal | Cu | Se | -0.04 | 0.08 | -0.19 | 0.11 | |
| SRS-2 Total | Infant | Cu | Se | 0.00 | 0.07 | -0.14 | 0.14 | |
| SRS-2 Total | Maternal Prenatal | Cu | Zn | 0.04 | 0.08 | -0.12 | 0.20 | |
| SRS-2 Total | Maternal Postnatal | Cu | Zn | 0.00 | 0.07 | -0.15 | 0.14 | |
| SRS-2 Total | Infant | Cu | Zn | 0.07 | 0.08 | -0.08 | 0.22 | |
| SRS-2 Total | Maternal Prenatal | Mn | As | 0.07 | 0.10 | -0.11 | 0.26 | |
| SRS-2 Total | Maternal Postnatal | Mn | As | 0.08 | 0.08 | -0.08 | 0.25 | |
| SRS-2 Total | Infant | Mn | As | -0.01 | 0.10 | -0.22 | 0.19 | |
| SRS-2 Total | Maternal Prenatal | Mn | Cu | -0.05 | 0.09 | -0.23 | 0.12 | |
| SRS-2 Total | Maternal Postnatal | Mn | Cu | -0.02 | 0.08 | -0.18 | 0.13 | |
| SRS-2 Total | Infant | Mn | Cu | 0.09 | 0.10 | -0.10 | 0.28 | |
| SRS-2 Total | Maternal Prenatal | Mn | Pb | 0.03 | 0.10 | -0.16 | 0.23 | |
| SRS-2 Total | Maternal Postnatal | Mn | Pb | 0.15 | 0.09 | -0.02 | 0.32 | |
| SRS-2 Total | Infant | Mn | Pb | -0.04 | 0.10 | -0.24 | 0.15 | |
| SRS-2 Total | Maternal Prenatal | Mn | Se | 0.17 | 0.10 | -0.03 | 0.38 | |
| SRS-2 Total | Maternal Postnatal | Mn | Se | -0.01 | 0.08 | -0.17 | 0.15 | |
| SRS-2 Total | Infant | Mn | Se | 0.03 | 0.09 | -0.16 | 0.21 | |
| SRS-2 Total | Maternal Prenatal | Mn | Zn | -0.12 | 0.10 | -0.32 | 0.08 | |
| SRS-2 Total | Maternal Postnatal | Mn | Zn | 0.03 | 0.08 | -0.13 | 0.19 | |
| SRS-2 Total | Infant | Mn | Zn | -0.04 | 0.10 | -0.22 | 0.15 | |
| SRS-2 Total | Maternal Prenatal | Pb | As | 0.12 | 0.10 | -0.08 | 0.31 | |
| SRS-2 Total | Maternal Postnatal | Pb | As | -0.04 | 0.08 | -0.20 | 0.13 | |
| SRS-2 Total | Infant | Pb | As | -0.04 | 0.12 | -0.28 | 0.20 | |
| SRS-2 Total | Maternal Prenatal | Pb | Cu | 0.08 | 0.10 | -0.11 | 0.27 | |
| SRS-2 Total | Maternal Postnatal | Pb | Cu | 0.04 | 0.08 | -0.11 | 0.20 | |
| SRS-2 Total | Infant | Pb | Cu | -0.16 | 0.10 | -0.36 | 0.04 | |
| SRS-2 Total | Maternal Prenatal | Pb | Mn | 0.03 | 0.10 | -0.17 | 0.24 | |
| SRS-2 Total | Maternal Postnatal | Pb | Mn | 0.15 | 0.09 | -0.02 | 0.32 | |
| SRS-2 Total | Infant | Pb | Mn | -0.04 | 0.10 | -0.25 | 0.16 | |
| SRS-2 Total | Maternal Prenatal | Pb | Se | -0.18 | 0.11 | -0.39 | 0.03 | |
| SRS-2 Total | Maternal Postnatal | Pb | Se | 0.00 | 0.08 | -0.16 | 0.15 | |
| SRS-2 Total | Infant | Pb | Se | -0.01 | 0.10 | -0.20 | 0.18 | |
| SRS-2 Total | Maternal Prenatal | Pb | Zn | -0.09 | 0.11 | -0.30 | 0.13 | |
| SRS-2 Total | Maternal Postnatal | Pb | Zn | -0.01 | 0.08 | -0.17 | 0.15 | |
| SRS-2 Total | Infant | Pb | Zn | 0.06 | 0.10 | -0.13 | 0.25 | |
| SRS-2 Total | Maternal Prenatal | Se | As | -0.02 | 0.09 | -0.19 | 0.15 | |
| SRS-2 Total | Maternal Postnatal | Se | As | 0.00 | 0.07 | -0.14 | 0.14 | |
| SRS-2 Total | Infant | Se | As | 0.15 | 0.05 | 0.05 | 0.26 | |
| SRS-2 Total | Maternal Prenatal | Se | Cu | 0.02 | 0.09 | -0.15 | 0.19 | |
| SRS-2 Total | Maternal Postnatal | Se | Cu | -0.04 | 0.07 | -0.18 | 0.10 | |
| SRS-2 Total | Infant | Se | Cu | 0.00 | 0.04 | -0.07 | 0.07 | |
| SRS-2 Total | Maternal Prenatal | Se | Mn | 0.17 | 0.10 | -0.02 | 0.37 | |
| SRS-2 Total | Maternal Postnatal | Se | Mn | -0.01 | 0.08 | -0.16 | 0.14 | |
| SRS-2 Total | Infant | Se | Mn | 0.03 | 0.05 | -0.06 | 0.11 | |
| SRS-2 Total | Maternal Prenatal | Se | Pb | -0.18 | 0.10 | -0.37 | 0.01 | |
| SRS-2 Total | Maternal Postnatal | Se | Pb | 0.00 | 0.08 | -0.15 | 0.14 | |
| SRS-2 Total | Infant | Se | Pb | -0.01 | 0.04 | -0.09 | 0.08 | |
| SRS-2 Total | Maternal Prenatal | Se | Zn | 0.02 | 0.09 | -0.15 | 0.20 | |
| SRS-2 Total | Maternal Postnatal | Se | Zn | -0.04 | 0.07 | -0.18 | 0.10 | |
| SRS-2 Total | Infant | Se | Zn | -0.04 | 0.03 | -0.11 | 0.03 | |
| SRS-2 Total | Maternal Prenatal | Zn | As | 0.01 | 0.10 | -0.18 | 0.20 | |
| SRS-2 Total | Maternal Postnatal | Zn | As | -0.05 | 0.07 | -0.18 | 0.09 | |
| SRS-2 Total | Infant | Zn | As | -0.12 | 0.07 | -0.26 | 0.02 | |
| SRS-2 Total | Maternal Prenatal | Zn | Cu | 0.04 | 0.09 | -0.14 | 0.21 | |
| SRS-2 Total | Maternal Postnatal | Zn | Cu | 0.00 | 0.07 | -0.13 | 0.13 | |
| SRS-2 Total | Infant | Zn | Cu | 0.07 | 0.05 | -0.03 | 0.18 | |
| SRS-2 Total | Maternal Prenatal | Zn | Mn | -0.12 | 0.10 | -0.32 | 0.08 | |
| SRS-2 Total | Maternal Postnatal | Zn | Mn | 0.03 | 0.07 | -0.11 | 0.18 | |
| SRS-2 Total | Infant | Zn | Mn | -0.04 | 0.06 | -0.16 | 0.09 | |
| SRS-2 Total | Maternal Prenatal | Zn | Pb | -0.09 | 0.11 | -0.30 | 0.12 | |
| SRS-2 Total | Maternal Postnatal | Zn | Pb | -0.01 | 0.07 | -0.15 | 0.13 | |
| SRS-2 Total | Infant | Zn | Pb | 0.06 | 0.06 | -0.06 | 0.18 | |
| SRS-2 Total | Maternal Prenatal | Zn | Se | 0.02 | 0.10 | -0.16 | 0.21 | |
| SRS-2 Total | Maternal Postnatal | Zn | Se | -0.04 | 0.07 | -0.17 | 0.09 | |
| SRS-2 Total | Infant | Zn | Se | -0.04 | 0.05 | -0.13 | 0.05 | |
| BASC-2 BSI | Maternal Prenatal | As | Cu | -0.01 | 0.08 | -0.17 | 0.15 | |
| BASC-2 BSI | Maternal Postnatal | As | Cu | -0.02 | 0.07 | -0.17 | 0.12 | |
| BASC-2 BSI | Infant | As | Cu | -0.05 | 0.13 | -0.31 | 0.20 | |
| BASC-2 BSI | Maternal Prenatal | As | Mn | -0.02 | 0.09 | -0.19 | 0.16 | |
| BASC-2 BSI | Maternal Postnatal | As | Mn | -0.03 | 0.08 | -0.18 | 0.13 | |
| BASC-2 BSI | Infant | As | Mn | -0.06 | 0.12 | -0.30 | 0.18 | |
| BASC-2 BSI | Maternal Prenatal | As | Pb | 0.05 | 0.09 | -0.13 | 0.23 | |
| BASC-2 BSI | Maternal Postnatal | As | Pb | -0.01 | 0.08 | -0.16 | 0.15 | |
| BASC-2 BSI | Infant | As | Pb | 0.11 | 0.14 | -0.17 | 0.38 | |
| BASC-2 BSI | Maternal Prenatal | As | Se | 0.16 | 0.09 | -0.01 | 0.33 | |
| BASC-2 BSI | Maternal Postnatal | As | Se | -0.01 | 0.07 | -0.15 | 0.14 | |
| BASC-2 BSI | Infant | As | Se | 0.10 | 0.12 | -0.13 | 0.32 | |
| BASC-2 BSI | Maternal Prenatal | As | Zn | -0.10 | 0.09 | -0.28 | 0.07 | |
| BASC-2 BSI | Maternal Postnatal | As | Zn | 0.00 | 0.07 | -0.15 | 0.14 | |
| BASC-2 BSI | Infant | As | Zn | -0.05 | 0.12 | -0.28 | 0.18 | |
| BASC-2 BSI | Maternal Prenatal | Cu | As | -0.01 | 0.09 | -0.18 | 0.17 | |
| BASC-2 BSI | Maternal Postnatal | Cu | As | -0.02 | 0.08 | -0.18 | 0.13 | |
| BASC-2 BSI | Infant | Cu | As | -0.05 | 0.10 | -0.25 | 0.15 | |
| BASC-2 BSI | Maternal Prenatal | Cu | Mn | -0.09 | 0.09 | -0.26 | 0.09 | |
| BASC-2 BSI | Maternal Postnatal | Cu | Mn | 0.10 | 0.08 | -0.06 | 0.26 | |
| BASC-2 BSI | Infant | Cu | Mn | 0.02 | 0.09 | -0.15 | 0.19 | |
| BASC-2 BSI | Maternal Prenatal | Cu | Pb | 0.05 | 0.09 | -0.13 | 0.23 | |
| BASC-2 BSI | Maternal Postnatal | Cu | Pb | 0.01 | 0.08 | -0.15 | 0.16 | |
| BASC-2 BSI | Infant | Cu | Pb | -0.04 | 0.09 | -0.21 | 0.13 | |
| BASC-2 BSI | Maternal Prenatal | Cu | Se | 0.00 | 0.09 | -0.18 | 0.17 | |
| BASC-2 BSI | Maternal Postnatal | Cu | Se | -0.03 | 0.08 | -0.18 | 0.12 | |
| BASC-2 BSI | Infant | Cu | Se | -0.01 | 0.07 | -0.15 | 0.14 | |
| BASC-2 BSI | Maternal Prenatal | Cu | Zn | 0.01 | 0.09 | -0.16 | 0.18 | |
| BASC-2 BSI | Maternal Postnatal | Cu | Zn | 0.02 | 0.08 | -0.13 | 0.17 | |
| BASC-2 BSI | Infant | Cu | Zn | 0.06 | 0.08 | -0.10 | 0.21 | |
| BASC-2 BSI | Maternal Prenatal | Mn | As | -0.02 | 0.10 | -0.21 | 0.18 | |
| BASC-2 BSI | Maternal Postnatal | Mn | As | -0.03 | 0.08 | -0.19 | 0.13 | |
| BASC-2 BSI | Infant | Mn | As | -0.06 | 0.11 | -0.27 | 0.15 | |
| BASC-2 BSI | Maternal Prenatal | Mn | Cu | -0.09 | 0.09 | -0.27 | 0.09 | |
| BASC-2 BSI | Maternal Postnatal | Mn | Cu | 0.10 | 0.08 | -0.05 | 0.26 | |
| BASC-2 BSI | Infant | Mn | Cu | 0.02 | 0.10 | -0.18 | 0.22 | |
| BASC-2 BSI | Maternal Prenatal | Mn | Pb | 0.10 | 0.11 | -0.11 | 0.31 | |
| BASC-2 BSI | Maternal Postnatal | Mn | Pb | 0.01 | 0.08 | -0.16 | 0.17 | |
| BASC-2 BSI | Infant | Mn | Pb | -0.03 | 0.11 | -0.25 | 0.19 | |
| BASC-2 BSI | Maternal Prenatal | Mn | Se | -0.05 | 0.11 | -0.26 | 0.16 | |
| BASC-2 BSI | Maternal Postnatal | Mn | Se | 0.02 | 0.08 | -0.14 | 0.18 | |
| BASC-2 BSI | Infant | Mn | Se | 0.02 | 0.10 | -0.17 | 0.21 | |
| BASC-2 BSI | Maternal Prenatal | Mn | Zn | -0.06 | 0.10 | -0.26 | 0.15 | |
| BASC-2 BSI | Maternal Postnatal | Mn | Zn | -0.01 | 0.08 | -0.16 | 0.15 | |
| BASC-2 BSI | Infant | Mn | Zn | 0.02 | 0.10 | -0.17 | 0.21 | |
| BASC-2 BSI | Maternal Prenatal | Pb | As | 0.05 | 0.10 | -0.16 | 0.25 | |
| BASC-2 BSI | Maternal Postnatal | Pb | As | -0.01 | 0.09 | -0.18 | 0.16 | |
| BASC-2 BSI | Infant | Pb | As | 0.11 | 0.13 | -0.15 | 0.37 | |
| BASC-2 BSI | Maternal Prenatal | Pb | Cu | 0.05 | 0.10 | -0.15 | 0.25 | |
| BASC-2 BSI | Maternal Postnatal | Pb | Cu | 0.01 | 0.08 | -0.15 | 0.17 | |
| BASC-2 BSI | Infant | Pb | Cu | -0.04 | 0.11 | -0.26 | 0.18 | |
| BASC-2 BSI | Maternal Prenatal | Pb | Mn | 0.10 | 0.11 | -0.11 | 0.31 | |
| BASC-2 BSI | Maternal Postnatal | Pb | Mn | 0.01 | 0.09 | -0.16 | 0.18 | |
| BASC-2 BSI | Infant | Pb | Mn | -0.03 | 0.12 | -0.26 | 0.21 | |
| BASC-2 BSI | Maternal Prenatal | Pb | Se | -0.03 | 0.11 | -0.24 | 0.18 | |
| BASC-2 BSI | Maternal Postnatal | Pb | Se | 0.02 | 0.08 | -0.15 | 0.18 | |
| BASC-2 BSI | Infant | Pb | Se | 0.02 | 0.10 | -0.18 | 0.22 | |
| BASC-2 BSI | Maternal Prenatal | Pb | Zn | 0.01 | 0.11 | -0.21 | 0.23 | |
| BASC-2 BSI | Maternal Postnatal | Pb | Zn | 0.00 | 0.08 | -0.16 | 0.16 | |
| BASC-2 BSI | Infant | Pb | Zn | -0.03 | 0.11 | -0.24 | 0.18 | |
| BASC-2 BSI | Maternal Prenatal | Se | As | 0.16 | 0.09 | -0.01 | 0.34 | |
| BASC-2 BSI | Maternal Postnatal | Se | As | -0.01 | 0.08 | -0.16 | 0.15 | |
| BASC-2 BSI | Infant | Se | As | 0.10 | 0.05 | 0.00 | 0.20 | |
| BASC-2 BSI | Maternal Prenatal | Se | Cu | 0.00 | 0.09 | -0.17 | 0.16 | |
| BASC-2 BSI | Maternal Postnatal | Se | Cu | -0.03 | 0.08 | -0.18 | 0.12 | |
| BASC-2 BSI | Infant | Se | Cu | -0.01 | 0.04 | -0.08 | 0.06 | |
| BASC-2 BSI | Maternal Prenatal | Se | Mn | -0.05 | 0.10 | -0.25 | 0.15 | |
| BASC-2 BSI | Maternal Postnatal | Se | Mn | 0.02 | 0.08 | -0.15 | 0.18 | |
| BASC-2 BSI | Infant | Se | Mn | 0.02 | 0.05 | -0.07 | 0.11 | |
| BASC-2 BSI | Maternal Prenatal | Se | Pb | -0.03 | 0.10 | -0.22 | 0.16 | |
| BASC-2 BSI | Maternal Postnatal | Se | Pb | 0.02 | 0.08 | -0.15 | 0.18 | |
| BASC-2 BSI | Infant | Se | Pb | 0.02 | 0.04 | -0.06 | 0.10 | |
| BASC-2 BSI | Maternal Prenatal | Se | Zn | 0.14 | 0.09 | -0.03 | 0.32 | |
| BASC-2 BSI | Maternal Postnatal | Se | Zn | -0.01 | 0.08 | -0.16 | 0.15 | |
| BASC-2 BSI | Infant | Se | Zn | -0.04 | 0.03 | -0.10 | 0.02 | |
| BASC-2 BSI | Maternal Prenatal | Zn | As | -0.10 | 0.09 | -0.29 | 0.08 | |
| BASC-2 BSI | Maternal Postnatal | Zn | As | 0.00 | 0.07 | -0.14 | 0.13 | |
| BASC-2 BSI | Infant | Zn | As | -0.05 | 0.07 | -0.18 | 0.08 | |
| BASC-2 BSI | Maternal Prenatal | Zn | Cu | 0.01 | 0.09 | -0.16 | 0.18 | |
| BASC-2 BSI | Maternal Postnatal | Zn | Cu | 0.02 | 0.07 | -0.10 | 0.15 | |
| BASC-2 BSI | Infant | Zn | Cu | 0.06 | 0.05 | -0.05 | 0.16 | |
| BASC-2 BSI | Maternal Prenatal | Zn | Mn | -0.06 | 0.10 | -0.25 | 0.14 | |
| BASC-2 BSI | Maternal Postnatal | Zn | Mn | -0.01 | 0.07 | -0.14 | 0.13 | |
| BASC-2 BSI | Infant | Zn | Mn | 0.02 | 0.06 | -0.11 | 0.14 | |
| BASC-2 BSI | Maternal Prenatal | Zn | Pb | 0.01 | 0.11 | -0.19 | 0.22 | |
| BASC-2 BSI | Maternal Postnatal | Zn | Pb | 0.00 | 0.07 | -0.14 | 0.14 | |
| BASC-2 BSI | Infant | Zn | Pb | -0.03 | 0.06 | -0.15 | 0.09 | |
| BASC-2 BSI | Maternal Prenatal | Zn | Se | 0.14 | 0.09 | -0.04 | 0.32 | |
| BASC-2 BSI | Maternal Postnatal | Zn | Se | -0.01 | 0.06 | -0.13 | 0.12 | |
| BASC-2 BSI | Infant | Zn | Se | -0.04 | 0.05 | -0.13 | 0.05 | |
| BASC-2 EXT | Maternal Prenatal | As | Cu | -0.01 | 0.08 | -0.17 | 0.15 | |
| BASC-2 EXT | Maternal Postnatal | As | Cu | -0.03 | 0.07 | -0.17 | 0.11 | |
| BASC-2 EXT | Infant | As | Cu | -0.01 | 0.13 | -0.26 | 0.24 | |
| BASC-2 EXT | Maternal Prenatal | As | Mn | -0.05 | 0.09 | -0.22 | 0.12 | |
| BASC-2 EXT | Maternal Postnatal | As | Mn | 0.00 | 0.08 | -0.16 | 0.15 | |
| BASC-2 EXT | Infant | As | Mn | -0.10 | 0.12 | -0.34 | 0.14 | |
| BASC-2 EXT | Maternal Prenatal | As | Pb | 0.03 | 0.09 | -0.15 | 0.20 | |
| BASC-2 EXT | Maternal Postnatal | As | Pb | -0.04 | 0.08 | -0.20 | 0.11 | |
| BASC-2 EXT | Infant | As | Pb | 0.09 | 0.14 | -0.18 | 0.36 | |
| BASC-2 EXT | Maternal Prenatal | As | Se | 0.14 | 0.08 | -0.02 | 0.31 | |
| BASC-2 EXT | Maternal Postnatal | As | Se | 0.05 | 0.07 | -0.09 | 0.20 | |
| BASC-2 EXT | Infant | As | Se | 0.04 | 0.11 | -0.18 | 0.26 | |
| BASC-2 EXT | Maternal Prenatal | As | Zn | -0.02 | 0.09 | -0.19 | 0.15 | |
| BASC-2 EXT | Maternal Postnatal | As | Zn | -0.02 | 0.07 | -0.17 | 0.12 | |
| BASC-2 EXT | Infant | As | Zn | 0.04 | 0.12 | -0.18 | 0.27 | |
| BASC-2 EXT | Maternal Prenatal | Cu | As | -0.01 | 0.09 | -0.18 | 0.16 | |
| BASC-2 EXT | Maternal Postnatal | Cu | As | -0.03 | 0.08 | -0.18 | 0.12 | |
| BASC-2 EXT | Infant | Cu | As | -0.01 | 0.10 | -0.20 | 0.19 | |
| BASC-2 EXT | Maternal Prenatal | Cu | Mn | -0.06 | 0.09 | -0.23 | 0.10 | |
| BASC-2 EXT | Maternal Postnatal | Cu | Mn | 0.09 | 0.08 | -0.07 | 0.25 | |
| BASC-2 EXT | Infant | Cu | Mn | -0.01 | 0.09 | -0.17 | 0.16 | |
| BASC-2 EXT | Maternal Prenatal | Cu | Pb | 0.03 | 0.09 | -0.15 | 0.21 | |
| BASC-2 EXT | Maternal Postnatal | Cu | Pb | -0.01 | 0.08 | -0.16 | 0.15 | |
| BASC-2 EXT | Infant | Cu | Pb | -0.03 | 0.09 | -0.20 | 0.14 | |
| BASC-2 EXT | Maternal Prenatal | Cu | Se | -0.03 | 0.09 | -0.20 | 0.15 | |
| BASC-2 EXT | Maternal Postnatal | Cu | Se | -0.06 | 0.08 | -0.21 | 0.10 | |
| BASC-2 EXT | Infant | Cu | Se | -0.02 | 0.07 | -0.17 | 0.12 | |
| BASC-2 EXT | Maternal Prenatal | Cu | Zn | -0.02 | 0.08 | -0.19 | 0.14 | |
| BASC-2 EXT | Maternal Postnatal | Cu | Zn | 0.03 | 0.08 | -0.12 | 0.18 | |
| BASC-2 EXT | Infant | Cu | Zn | 0.05 | 0.08 | -0.10 | 0.20 | |
| BASC-2 EXT | Maternal Prenatal | Mn | As | -0.05 | 0.10 | -0.24 | 0.14 | |
| BASC-2 EXT | Maternal Postnatal | Mn | As | 0.00 | 0.08 | -0.16 | 0.16 | |
| BASC-2 EXT | Infant | Mn | As | -0.10 | 0.11 | -0.31 | 0.11 | |
| BASC-2 EXT | Maternal Prenatal | Mn | Cu | -0.06 | 0.09 | -0.24 | 0.11 | |
| BASC-2 EXT | Maternal Postnatal | Mn | Cu | 0.09 | 0.08 | -0.06 | 0.24 | |
| BASC-2 EXT | Infant | Mn | Cu | -0.01 | 0.10 | -0.20 | 0.19 | |
| BASC-2 EXT | Maternal Prenatal | Mn | Pb | 0.07 | 0.10 | -0.13 | 0.27 | |
| BASC-2 EXT | Maternal Postnatal | Mn | Pb | -0.03 | 0.08 | -0.19 | 0.13 | |
| BASC-2 EXT | Infant | Mn | Pb | 0.00 | 0.11 | -0.22 | 0.21 | |
| BASC-2 EXT | Maternal Prenatal | Mn | Se | -0.15 | 0.10 | -0.36 | 0.05 | |
| BASC-2 EXT | Maternal Postnatal | Mn | Se | 0.01 | 0.08 | -0.14 | 0.17 | |
| BASC-2 EXT | Infant | Mn | Se | 0.01 | 0.09 | -0.18 | 0.20 | |
| BASC-2 EXT | Maternal Prenatal | Mn | Zn | 0.02 | 0.10 | -0.18 | 0.21 | |
| BASC-2 EXT | Maternal Postnatal | Mn | Zn | 0.03 | 0.08 | -0.12 | 0.19 | |
| BASC-2 EXT | Infant | Mn | Zn | 0.01 | 0.10 | -0.18 | 0.20 | |
| BASC-2 EXT | Maternal Prenatal | Pb | As | 0.03 | 0.10 | -0.17 | 0.22 | |
| BASC-2 EXT | Maternal Postnatal | Pb | As | -0.04 | 0.08 | -0.21 | 0.12 | |
| BASC-2 EXT | Infant | Pb | As | 0.09 | 0.13 | -0.17 | 0.34 | |
| BASC-2 EXT | Maternal Prenatal | Pb | Cu | 0.03 | 0.10 | -0.16 | 0.22 | |
| BASC-2 EXT | Maternal Postnatal | Pb | Cu | -0.01 | 0.08 | -0.16 | 0.15 | |
| BASC-2 EXT | Infant | Pb | Cu | -0.03 | 0.11 | -0.24 | 0.19 | |
| BASC-2 EXT | Maternal Prenatal | Pb | Mn | 0.07 | 0.11 | -0.13 | 0.28 | |
| BASC-2 EXT | Maternal Postnatal | Pb | Mn | -0.03 | 0.08 | -0.20 | 0.13 | |
| BASC-2 EXT | Infant | Pb | Mn | 0.00 | 0.12 | -0.24 | 0.23 | |
| BASC-2 EXT | Maternal Prenatal | Pb | Se | 0.03 | 0.11 | -0.18 | 0.23 | |
| BASC-2 EXT | Maternal Postnatal | Pb | Se | -0.02 | 0.08 | -0.18 | 0.14 | |
| BASC-2 EXT | Infant | Pb | Se | 0.07 | 0.10 | -0.13 | 0.27 | |
| BASC-2 EXT | Maternal Prenatal | Pb | Zn | -0.01 | 0.11 | -0.22 | 0.20 | |
| BASC-2 EXT | Maternal Postnatal | Pb | Zn | 0.01 | 0.08 | -0.15 | 0.17 | |
| BASC-2 EXT | Infant | Pb | Zn | -0.10 | 0.10 | -0.30 | 0.10 | |
| BASC-2 EXT | Maternal Prenatal | Se | As | 0.14 | 0.09 | -0.03 | 0.31 | |
| BASC-2 EXT | Maternal Postnatal | Se | As | 0.05 | 0.08 | -0.11 | 0.21 | |
| BASC-2 EXT | Infant | Se | As | 0.04 | 0.05 | -0.06 | 0.14 | |
| BASC-2 EXT | Maternal Prenatal | Se | Cu | -0.03 | 0.08 | -0.19 | 0.14 | |
| BASC-2 EXT | Maternal Postnatal | Se | Cu | -0.06 | 0.08 | -0.21 | 0.10 | |
| BASC-2 EXT | Infant | Se | Cu | -0.02 | 0.03 | -0.09 | 0.04 | |
| BASC-2 EXT | Maternal Prenatal | Se | Mn | -0.15 | 0.10 | -0.34 | 0.04 | |
| BASC-2 EXT | Maternal Postnatal | Se | Mn | 0.01 | 0.09 | -0.15 | 0.18 | |
| BASC-2 EXT | Infant | Se | Mn | 0.01 | 0.04 | -0.08 | 0.10 | |
| BASC-2 EXT | Maternal Prenatal | Se | Pb | 0.03 | 0.10 | -0.16 | 0.21 | |
| BASC-2 EXT | Maternal Postnatal | Se | Pb | -0.02 | 0.08 | -0.18 | 0.15 | |
| BASC-2 EXT | Infant | Se | Pb | 0.07 | 0.04 | -0.01 | 0.15 | |
| BASC-2 EXT | Maternal Prenatal | Se | Zn | 0.13 | 0.09 | -0.04 | 0.30 | |
| BASC-2 EXT | Maternal Postnatal | Se | Zn | 0.02 | 0.08 | -0.13 | 0.18 | |
| BASC-2 EXT | Infant | Se | Zn | -0.02 | 0.03 | -0.08 | 0.05 | |
| BASC-2 EXT | Maternal Prenatal | Zn | As | -0.02 | 0.09 | -0.20 | 0.16 | |
| BASC-2 EXT | Maternal Postnatal | Zn | As | -0.02 | 0.07 | -0.16 | 0.12 | |
| BASC-2 EXT | Infant | Zn | As | 0.04 | 0.07 | -0.09 | 0.18 | |
| BASC-2 EXT | Maternal Prenatal | Zn | Cu | -0.02 | 0.08 | -0.19 | 0.14 | |
| BASC-2 EXT | Maternal Postnatal | Zn | Cu | 0.03 | 0.07 | -0.10 | 0.16 | |
| BASC-2 EXT | Infant | Zn | Cu | 0.05 | 0.05 | -0.05 | 0.15 | |
| BASC-2 EXT | Maternal Prenatal | Zn | Mn | 0.02 | 0.10 | -0.18 | 0.21 | |
| BASC-2 EXT | Maternal Postnatal | Zn | Mn | 0.03 | 0.07 | -0.11 | 0.18 | |
| BASC-2 EXT | Infant | Zn | Mn | 0.01 | 0.06 | -0.11 | 0.13 | |
| BASC-2 EXT | Maternal Prenatal | Zn | Pb | -0.01 | 0.10 | -0.21 | 0.19 | |
| BASC-2 EXT | Maternal Postnatal | Zn | Pb | 0.01 | 0.07 | -0.13 | 0.15 | |
| BASC-2 EXT | Infant | Zn | Pb | -0.10 | 0.06 | -0.21 | 0.02 | |
| BASC-2 EXT | Maternal Prenatal | Zn | Se | 0.13 | 0.09 | -0.04 | 0.31 | |
| BASC-2 EXT | Maternal Postnatal | Zn | Se | 0.02 | 0.07 | -0.11 | 0.15 | |
| BASC-2 EXT | Infant | Zn | Se | -0.02 | 0.05 | -0.11 | 0.08 | |
| BASC-2 INZ | Maternal Prenatal | As | Cu | 0.00 | 0.09 | -0.19 | 0.18 | |
| BASC-2 INZ | Maternal Postnatal | As | Cu | -0.04 | 0.08 | -0.20 | 0.12 | |
| BASC-2 INZ | Infant | As | Cu | -0.01 | 0.14 | -0.29 | 0.27 | |
| BASC-2 INZ | Maternal Prenatal | As | Mn | -0.03 | 0.10 | -0.22 | 0.17 | |
| BASC-2 INZ | Maternal Postnatal | As | Mn | -0.08 | 0.09 | -0.25 | 0.10 | |
| BASC-2 INZ | Infant | As | Mn | 0.00 | 0.14 | -0.27 | 0.26 | |
| BASC-2 INZ | Maternal Prenatal | As | Pb | 0.11 | 0.10 | -0.10 | 0.31 | |
| BASC-2 INZ | Maternal Postnatal | As | Pb | -0.01 | 0.09 | -0.18 | 0.17 | |
| BASC-2 INZ | Infant | As | Pb | -0.01 | 0.15 | -0.31 | 0.30 | |
| BASC-2 INZ | Maternal Prenatal | As | Se | 0.17 | 0.10 | -0.02 | 0.37 | |
| BASC-2 INZ | Maternal Postnatal | As | Se | -0.01 | 0.08 | -0.18 | 0.15 | |
| BASC-2 INZ | Infant | As | Se | -0.06 | 0.13 | -0.31 | 0.18 | |
| BASC-2 INZ | Maternal Prenatal | As | Zn | -0.15 | 0.10 | -0.35 | 0.04 | |
| BASC-2 INZ | Maternal Postnatal | As | Zn | 0.05 | 0.08 | -0.12 | 0.21 | |
| BASC-2 INZ | Infant | As | Zn | 0.09 | 0.13 | -0.17 | 0.34 | |
| BASC-2 INZ | Maternal Prenatal | Cu | As | 0.00 | 0.10 | -0.20 | 0.19 | |
| BASC-2 INZ | Maternal Postnatal | Cu | As | -0.04 | 0.09 | -0.21 | 0.13 | |
| BASC-2 INZ | Infant | Cu | As | -0.01 | 0.11 | -0.23 | 0.20 | |
| BASC-2 INZ | Maternal Prenatal | Cu | Mn | -0.08 | 0.10 | -0.27 | 0.12 | |
| BASC-2 INZ | Maternal Postnatal | Cu | Mn | 0.04 | 0.09 | -0.14 | 0.22 | |
| BASC-2 INZ | Infant | Cu | Mn | -0.03 | 0.10 | -0.22 | 0.16 | |
| BASC-2 INZ | Maternal Prenatal | Cu | Pb | 0.08 | 0.11 | -0.12 | 0.29 | |
| BASC-2 INZ | Maternal Postnatal | Cu | Pb | 0.03 | 0.09 | -0.14 | 0.21 | |
| BASC-2 INZ | Infant | Cu | Pb | 0.07 | 0.10 | -0.12 | 0.25 | |
| BASC-2 INZ | Maternal Prenatal | Cu | Se | 0.10 | 0.10 | -0.10 | 0.30 | |
| BASC-2 INZ | Maternal Postnatal | Cu | Se | 0.02 | 0.09 | -0.15 | 0.19 | |
| BASC-2 INZ | Infant | Cu | Se | -0.03 | 0.08 | -0.18 | 0.13 | |
| BASC-2 INZ | Maternal Prenatal | Cu | Zn | -0.08 | 0.10 | -0.27 | 0.12 | |
| BASC-2 INZ | Maternal Postnatal | Cu | Zn | -0.01 | 0.09 | -0.18 | 0.16 | |
| BASC-2 INZ | Infant | Cu | Zn | 0.06 | 0.09 | -0.11 | 0.22 | |
| BASC-2 INZ | Maternal Prenatal | Mn | As | -0.03 | 0.11 | -0.25 | 0.20 | |
| BASC-2 INZ | Maternal Postnatal | Mn | As | -0.08 | 0.09 | -0.26 | 0.10 | |
| BASC-2 INZ | Infant | Mn | As | 0.00 | 0.12 | -0.23 | 0.23 | |
| BASC-2 INZ | Maternal Prenatal | Mn | Cu | -0.08 | 0.10 | -0.28 | 0.12 | |
| BASC-2 INZ | Maternal Postnatal | Mn | Cu | 0.04 | 0.09 | -0.13 | 0.21 | |
| BASC-2 INZ | Infant | Mn | Cu | -0.03 | 0.11 | -0.25 | 0.19 | |
| BASC-2 INZ | Maternal Prenatal | Mn | Pb | 0.03 | 0.12 | -0.21 | 0.26 | |
| BASC-2 INZ | Maternal Postnatal | Mn | Pb | -0.02 | 0.09 | -0.20 | 0.17 | |
| BASC-2 INZ | Infant | Mn | Pb | 0.00 | 0.12 | -0.24 | 0.24 | |
| BASC-2 INZ | Maternal Prenatal | Mn | Se | -0.05 | 0.12 | -0.29 | 0.19 | |
| BASC-2 INZ | Maternal Postnatal | Mn | Se | 0.03 | 0.09 | -0.15 | 0.20 | |
| BASC-2 INZ | Infant | Mn | Se | 0.02 | 0.10 | -0.18 | 0.23 | |
| BASC-2 INZ | Maternal Prenatal | Mn | Zn | -0.04 | 0.12 | -0.27 | 0.19 | |
| BASC-2 INZ | Maternal Postnatal | Mn | Zn | -0.04 | 0.09 | -0.22 | 0.13 | |
| BASC-2 INZ | Infant | Mn | Zn | 0.00 | 0.11 | -0.21 | 0.21 | |
| BASC-2 INZ | Maternal Prenatal | Pb | As | 0.11 | 0.12 | -0.12 | 0.33 | |
| BASC-2 INZ | Maternal Postnatal | Pb | As | -0.01 | 0.10 | -0.19 | 0.18 | |
| BASC-2 INZ | Infant | Pb | As | -0.01 | 0.14 | -0.29 | 0.28 | |
| BASC-2 INZ | Maternal Prenatal | Pb | Cu | 0.08 | 0.11 | -0.14 | 0.31 | |
| BASC-2 INZ | Maternal Postnatal | Pb | Cu | 0.03 | 0.09 | -0.14 | 0.21 | |
| BASC-2 INZ | Infant | Pb | Cu | 0.07 | 0.12 | -0.17 | 0.30 | |
| BASC-2 INZ | Maternal Prenatal | Pb | Mn | 0.03 | 0.12 | -0.21 | 0.27 | |
| BASC-2 INZ | Maternal Postnatal | Pb | Mn | -0.02 | 0.10 | -0.20 | 0.17 | |
| BASC-2 INZ | Infant | Pb | Mn | 0.00 | 0.13 | -0.26 | 0.25 | |
| BASC-2 INZ | Maternal Prenatal | Pb | Se | -0.11 | 0.12 | -0.35 | 0.13 | |
| BASC-2 INZ | Maternal Postnatal | Pb | Se | 0.05 | 0.09 | -0.14 | 0.23 | |
| BASC-2 INZ | Infant | Pb | Se | 0.07 | 0.11 | -0.15 | 0.29 | |
| BASC-2 INZ | Maternal Prenatal | Pb | Zn | 0.25 | 0.13 | 0.00 | 0.49 | |
| BASC-2 INZ | Maternal Postnatal | Pb | Zn | -0.04 | 0.09 | -0.22 | 0.14 | |
| BASC-2 INZ | Infant | Pb | Zn | -0.07 | 0.12 | -0.30 | 0.15 | |
| BASC-2 INZ | Maternal Prenatal | Se | As | 0.17 | 0.10 | -0.02 | 0.37 | |
| BASC-2 INZ | Maternal Postnatal | Se | As | -0.01 | 0.09 | -0.19 | 0.16 | |
| BASC-2 INZ | Infant | Se | As | -0.06 | 0.06 | -0.18 | 0.05 | |
| BASC-2 INZ | Maternal Prenatal | Se | Cu | 0.10 | 0.10 | -0.09 | 0.29 | |
| BASC-2 INZ | Maternal Postnatal | Se | Cu | 0.02 | 0.09 | -0.16 | 0.19 | |
| BASC-2 INZ | Infant | Se | Cu | -0.03 | 0.04 | -0.10 | 0.05 | |
| BASC-2 INZ | Maternal Prenatal | Se | Mn | -0.05 | 0.11 | -0.28 | 0.17 | |
| BASC-2 INZ | Maternal Postnatal | Se | Mn | 0.03 | 0.09 | -0.16 | 0.21 | |
| BASC-2 INZ | Infant | Se | Mn | 0.02 | 0.05 | -0.07 | 0.12 | |
| BASC-2 INZ | Maternal Prenatal | Se | Pb | -0.11 | 0.11 | -0.33 | 0.10 | |
| BASC-2 INZ | Maternal Postnatal | Se | Pb | 0.05 | 0.09 | -0.13 | 0.23 | |
| BASC-2 INZ | Infant | Se | Pb | 0.07 | 0.05 | -0.02 | 0.16 | |
| BASC-2 INZ | Maternal Prenatal | Se | Zn | 0.16 | 0.10 | -0.04 | 0.35 | |
| BASC-2 INZ | Maternal Postnatal | Se | Zn | -0.01 | 0.09 | -0.18 | 0.16 | |
| BASC-2 INZ | Infant | Se | Zn | -0.05 | 0.04 | -0.12 | 0.03 | |
| BASC-2 INZ | Maternal Prenatal | Zn | As | -0.15 | 0.11 | -0.36 | 0.05 | |
| BASC-2 INZ | Maternal Postnatal | Zn | As | 0.05 | 0.08 | -0.10 | 0.19 | |
| BASC-2 INZ | Infant | Zn | As | 0.09 | 0.07 | -0.06 | 0.23 | |
| BASC-2 INZ | Maternal Prenatal | Zn | Cu | -0.08 | 0.10 | -0.27 | 0.11 | |
| BASC-2 INZ | Maternal Postnatal | Zn | Cu | -0.01 | 0.07 | -0.15 | 0.14 | |
| BASC-2 INZ | Infant | Zn | Cu | 0.06 | 0.06 | -0.06 | 0.17 | |
| BASC-2 INZ | Maternal Prenatal | Zn | Mn | -0.04 | 0.11 | -0.26 | 0.18 | |
| BASC-2 INZ | Maternal Postnatal | Zn | Mn | -0.04 | 0.08 | -0.20 | 0.11 | |
| BASC-2 INZ | Infant | Zn | Mn | 0.00 | 0.07 | -0.14 | 0.14 | |
| BASC-2 INZ | Maternal Prenatal | Zn | Pb | 0.25 | 0.12 | 0.01 | 0.48 | |
| BASC-2 INZ | Maternal Postnatal | Zn | Pb | -0.04 | 0.08 | -0.20 | 0.11 | |
| BASC-2 INZ | Infant | Zn | Pb | -0.07 | 0.07 | -0.20 | 0.06 | |
| BASC-2 INZ | Maternal Prenatal | Zn | Se | 0.16 | 0.10 | -0.05 | 0.36 | |
| BASC-2 INZ | Maternal Postnatal | Zn | Se | -0.01 | 0.07 | -0.16 | 0.13 | |
| BASC-2 INZ | Infant | Zn | Se | -0.05 | 0.05 | -0.15 | 0.06 | |
| BASC-2 AKL | Maternal Prenatal | As | Cu | 0.00 | 0.08 | -0.17 | 0.16 | |
| BASC-2 AKL | Maternal Postnatal | As | Cu | 0.00 | 0.08 | -0.14 | 0.15 | |
| BASC-2 AKL | Infant | As | Cu | 0.18 | 0.14 | -0.08 | 0.45 | |
| BASC-2 AKL | Maternal Prenatal | As | Mn | 0.10 | 0.09 | -0.08 | 0.28 | |
| BASC-2 AKL | Maternal Postnatal | As | Mn | 0.01 | 0.08 | -0.16 | 0.17 | |
| BASC-2 AKL | Infant | As | Mn | 0.09 | 0.13 | -0.16 | 0.34 | |
| BASC-2 AKL | Maternal Prenatal | As | Pb | -0.02 | 0.09 | -0.21 | 0.16 | |
| BASC-2 AKL | Maternal Postnatal | As | Pb | -0.04 | 0.08 | -0.21 | 0.12 | |
| BASC-2 AKL | Infant | As | Pb | -0.16 | 0.15 | -0.44 | 0.13 | |
| BASC-2 AKL | Maternal Prenatal | As | Se | -0.08 | 0.09 | -0.26 | 0.09 | |
| BASC-2 AKL | Maternal Postnatal | As | Se | 0.11 | 0.08 | -0.05 | 0.26 | |
| BASC-2 AKL | Infant | As | Se | -0.18 | 0.12 | -0.41 | 0.06 | |
| BASC-2 AKL | Maternal Prenatal | As | Zn | -0.01 | 0.09 | -0.19 | 0.16 | |
| BASC-2 AKL | Maternal Postnatal | As | Zn | 0.00 | 0.08 | -0.16 | 0.15 | |
| BASC-2 AKL | Infant | As | Zn | 0.11 | 0.12 | -0.13 | 0.35 | |
| BASC-2 AKL | Maternal Prenatal | Cu | As | 0.00 | 0.09 | -0.18 | 0.17 | |
| BASC-2 AKL | Maternal Postnatal | Cu | As | 0.00 | 0.08 | -0.15 | 0.16 | |
| BASC-2 AKL | Infant | Cu | As | 0.18 | 0.10 | -0.02 | 0.39 | |
| BASC-2 AKL | Maternal Prenatal | Cu | Mn | 0.02 | 0.09 | -0.16 | 0.20 | |
| BASC-2 AKL | Maternal Postnatal | Cu | Mn | -0.04 | 0.09 | -0.21 | 0.13 | |
| BASC-2 AKL | Infant | Cu | Mn | -0.09 | 0.09 | -0.27 | 0.09 | |
| BASC-2 AKL | Maternal Prenatal | Cu | Pb | -0.04 | 0.09 | -0.23 | 0.14 | |
| BASC-2 AKL | Maternal Postnatal | Cu | Pb | -0.01 | 0.08 | -0.17 | 0.15 | |
| BASC-2 AKL | Infant | Cu | Pb | 0.11 | 0.09 | -0.07 | 0.29 | |
| BASC-2 AKL | Maternal Prenatal | Cu | Se | 0.01 | 0.09 | -0.17 | 0.19 | |
| BASC-2 AKL | Maternal Postnatal | Cu | Se | 0.03 | 0.08 | -0.14 | 0.19 | |
| BASC-2 AKL | Infant | Cu | Se | -0.02 | 0.08 | -0.17 | 0.13 | |
| BASC-2 AKL | Maternal Prenatal | Cu | Zn | -0.01 | 0.09 | -0.18 | 0.17 | |
| BASC-2 AKL | Maternal Postnatal | Cu | Zn | 0.00 | 0.08 | -0.16 | 0.16 | |
| BASC-2 AKL | Infant | Cu | Zn | -0.02 | 0.08 | -0.18 | 0.14 | |
| BASC-2 AKL | Maternal Prenatal | Mn | As | 0.10 | 0.10 | -0.10 | 0.30 | |
| BASC-2 AKL | Maternal Postnatal | Mn | As | 0.01 | 0.09 | -0.16 | 0.17 | |
| BASC-2 AKL | Infant | Mn | As | 0.09 | 0.11 | -0.13 | 0.31 | |
| BASC-2 AKL | Maternal Prenatal | Mn | Cu | 0.02 | 0.09 | -0.16 | 0.20 | |
| BASC-2 AKL | Maternal Postnatal | Mn | Cu | -0.04 | 0.08 | -0.20 | 0.12 | |
| BASC-2 AKL | Infant | Mn | Cu | -0.09 | 0.11 | -0.30 | 0.12 | |
| BASC-2 AKL | Maternal Prenatal | Mn | Pb | -0.03 | 0.11 | -0.24 | 0.18 | |
| BASC-2 AKL | Maternal Postnatal | Mn | Pb | -0.12 | 0.09 | -0.29 | 0.05 | |
| BASC-2 AKL | Infant | Mn | Pb | 0.07 | 0.12 | -0.16 | 0.30 | |
| BASC-2 AKL | Maternal Prenatal | Mn | Se | 0.01 | 0.11 | -0.21 | 0.22 | |
| BASC-2 AKL | Maternal Postnatal | Mn | Se | -0.05 | 0.09 | -0.21 | 0.12 | |
| BASC-2 AKL | Infant | Mn | Se | 0.00 | 0.10 | -0.20 | 0.19 | |
| BASC-2 AKL | Maternal Prenatal | Mn | Zn | 0.18 | 0.11 | -0.02 | 0.39 | |
| BASC-2 AKL | Maternal Postnatal | Mn | Zn | 0.02 | 0.08 | -0.14 | 0.18 | |
| BASC-2 AKL | Infant | Mn | Zn | -0.02 | 0.10 | -0.22 | 0.18 | |
| BASC-2 AKL | Maternal Prenatal | Pb | As | -0.02 | 0.11 | -0.23 | 0.18 | |
| BASC-2 AKL | Maternal Postnatal | Pb | As | -0.04 | 0.09 | -0.22 | 0.13 | |
| BASC-2 AKL | Infant | Pb | As | -0.16 | 0.14 | -0.43 | 0.11 | |
| BASC-2 AKL | Maternal Prenatal | Pb | Cu | -0.04 | 0.10 | -0.24 | 0.16 | |
| BASC-2 AKL | Maternal Postnatal | Pb | Cu | -0.01 | 0.08 | -0.17 | 0.16 | |
| BASC-2 AKL | Infant | Pb | Cu | 0.11 | 0.12 | -0.12 | 0.34 | |
| BASC-2 AKL | Maternal Prenatal | Pb | Mn | -0.03 | 0.11 | -0.25 | 0.18 | |
| BASC-2 AKL | Maternal Postnatal | Pb | Mn | -0.12 | 0.09 | -0.29 | 0.06 | |
| BASC-2 AKL | Infant | Pb | Mn | 0.07 | 0.13 | -0.17 | 0.32 | |
| BASC-2 AKL | Maternal Prenatal | Pb | Se | 0.19 | 0.11 | -0.03 | 0.40 | |
| BASC-2 AKL | Maternal Postnatal | Pb | Se | -0.01 | 0.09 | -0.18 | 0.17 | |
| BASC-2 AKL | Infant | Pb | Se | 0.05 | 0.11 | -0.16 | 0.27 | |
| BASC-2 AKL | Maternal Prenatal | Pb | Zn | -0.05 | 0.11 | -0.27 | 0.18 | |
| BASC-2 AKL | Maternal Postnatal | Pb | Zn | -0.06 | 0.09 | -0.22 | 0.11 | |
| BASC-2 AKL | Infant | Pb | Zn | -0.09 | 0.11 | -0.31 | 0.12 | |
| BASC-2 AKL | Maternal Prenatal | Se | As | -0.08 | 0.09 | -0.26 | 0.09 | |
| BASC-2 AKL | Maternal Postnatal | Se | As | 0.11 | 0.09 | -0.07 | 0.28 | |
| BASC-2 AKL | Infant | Se | As | -0.18 | 0.05 | -0.28 | -0.07 | |
| BASC-2 AKL | Maternal Prenatal | Se | Cu | 0.01 | 0.09 | -0.16 | 0.18 | |
| BASC-2 AKL | Maternal Postnatal | Se | Cu | 0.03 | 0.09 | -0.14 | 0.19 | |
| BASC-2 AKL | Infant | Se | Cu | -0.02 | 0.04 | -0.09 | 0.05 | |
| BASC-2 AKL | Maternal Prenatal | Se | Mn | 0.01 | 0.10 | -0.20 | 0.21 | |
| BASC-2 AKL | Maternal Postnatal | Se | Mn | -0.05 | 0.09 | -0.23 | 0.13 | |
| BASC-2 AKL | Infant | Se | Mn | 0.00 | 0.05 | -0.09 | 0.09 | |
| BASC-2 AKL | Maternal Prenatal | Se | Pb | 0.19 | 0.10 | -0.01 | 0.38 | |
| BASC-2 AKL | Maternal Postnatal | Se | Pb | -0.01 | 0.09 | -0.18 | 0.17 | |
| BASC-2 AKL | Infant | Se | Pb | 0.05 | 0.04 | -0.03 | 0.14 | |
| BASC-2 AKL | Maternal Prenatal | Se | Zn | -0.05 | 0.09 | -0.23 | 0.13 | |
| BASC-2 AKL | Maternal Postnatal | Se | Zn | 0.02 | 0.08 | -0.14 | 0.19 | |
| BASC-2 AKL | Infant | Se | Zn | 0.02 | 0.03 | -0.04 | 0.09 | |
| BASC-2 AKL | Maternal Prenatal | Zn | As | -0.01 | 0.10 | -0.20 | 0.18 | |
| BASC-2 AKL | Maternal Postnatal | Zn | As | 0.00 | 0.07 | -0.15 | 0.15 | |
| BASC-2 AKL | Infant | Zn | As | 0.11 | 0.07 | -0.03 | 0.25 | |
| BASC-2 AKL | Maternal Prenatal | Zn | Cu | -0.01 | 0.09 | -0.18 | 0.17 | |
| BASC-2 AKL | Maternal Postnatal | Zn | Cu | 0.00 | 0.07 | -0.14 | 0.14 | |
| BASC-2 AKL | Infant | Zn | Cu | -0.02 | 0.05 | -0.12 | 0.09 | |
| BASC-2 AKL | Maternal Prenatal | Zn | Mn | 0.18 | 0.10 | -0.02 | 0.39 | |
| BASC-2 AKL | Maternal Postnatal | Zn | Mn | 0.02 | 0.08 | -0.13 | 0.17 | |
| BASC-2 AKL | Infant | Zn | Mn | -0.02 | 0.07 | -0.15 | 0.11 | |
| BASC-2 AKL | Maternal Prenatal | Zn | Pb | -0.05 | 0.11 | -0.26 | 0.16 | |
| BASC-2 AKL | Maternal Postnatal | Zn | Pb | -0.06 | 0.08 | -0.21 | 0.10 | |
| BASC-2 AKL | Infant | Zn | Pb | -0.09 | 0.06 | -0.21 | 0.03 | |
| BASC-2 AKL | Maternal Prenatal | Zn | Se | -0.05 | 0.09 | -0.23 | 0.14 | |
| BASC-2 AKL | Maternal Postnatal | Zn | Se | 0.02 | 0.07 | -0.12 | 0.17 | |
| BASC-2 AKL | Infant | Zn | Se | 0.02 | 0.05 | -0.07 | 0.12 | |
| Estimate is the difference in the IQR contrast for metal 1 at 75% of metal 2 versus 25% of metal 2. Estimate = [E(Outcome\|Metal 1 = 75%, Metal 2 = 75%) - E(Outcome\|Metal 1 = 25%, Metal 2 = 75%)] - [E(Outcome\|Metal 1 = 75%, Metal 2 = 25%) - E(Outcome\|Metal 1 = 25%, Metal 2 = 25%)].  Models adjusted for maternal age (quadratic), maternal BMI (quadratic), highest level of parental education (high school or less, any college, any graduate), sex (male, female), parity (0, ≥1), smoking status (no second- or first-hand, ever second-hand only, ever first-hand), age at last breastfeeding (<365 days, ≥365 days), maternal marital status (married, other), birthyear (2010-2011, 2012-2013, 2014-2015), Healthy Eating Index (linear), Parenting Relationship Questionnaire (first three principal components), and age at assessment (linear).  Abbreviations: AKL, Adaptive Skills; BASC-2, Behavioral Assessment System for Children, 2^nd^ Ed.; BSI, Behavioral Symptoms Index; CrI, Credible Interval; EXT, Externalizing Problems; INZ, Internalizing Problems; IQR, Interquartile Range; SRS-2, Social Responsiveness Scale, 2^nd^ Ed. | | | | | | | |  |

| Supplemental Table S6. Main effects of each metal at each time point, among male children. | | | | | | |
| --- | --- | --- | --- | --- | --- | --- |
| Outcome | Time | Metal | Estimate | SD | 95% CrI Low | 95% CrI High |
| SRS-2 Total | Maternal Prenatal | As | -0.10 | 0.07 | -0.24 | 0.04 |
| SRS-2 Total | Maternal Prenatal | Cu | 0.08 | 0.07 | -0.05 | 0.21 |
| SRS-2 Total | Maternal Prenatal | Mn | 0.04 | 0.08 | -0.11 | 0.19 |
| SRS-2 Total | Maternal Prenatal | Pb | -0.06 | 0.09 | -0.23 | 0.11 |
| SRS-2 Total | Maternal Prenatal | Se | -0.02 | 0.08 | -0.17 | 0.12 |
| SRS-2 Total | Maternal Prenatal | Zn | 0.01 | 0.09 | -0.16 | 0.18 |
| SRS-2 Total | Maternal Postnatal | As | 0.04 | 0.06 | -0.08 | 0.15 |
| SRS-2 Total | Maternal Postnatal | Cu | 0.02 | 0.06 | -0.09 | 0.13 |
| SRS-2 Total | Maternal Postnatal | Mn | 0.02 | 0.07 | -0.11 | 0.15 |
| SRS-2 Total | Maternal Postnatal | Pb | -0.01 | 0.07 | -0.14 | 0.13 |
| SRS-2 Total | Maternal Postnatal | Se | 0.01 | 0.07 | -0.13 | 0.16 |
| SRS-2 Total | Maternal Postnatal | Zn | -0.07 | 0.06 | -0.19 | 0.04 |
| SRS-2 Total | Infant | As | 0.03 | 0.10 | -0.16 | 0.22 |
| SRS-2 Total | Infant | Cu | -0.18 | 0.07 | -0.32 | -0.05 |
| SRS-2 Total | Infant | Mn | 0.20 | 0.09 | 0.01 | 0.38 |
| SRS-2 Total | Infant | Pb | -0.08 | 0.09 | -0.25 | 0.10 |
| SRS-2 Total | Infant | Se | 0.00 | 0.03 | -0.05 | 0.05 |
| SRS-2 Total | Infant | Zn | 0.09 | 0.06 | -0.03 | 0.20 |
| BASC-2 BSI | Maternal Prenatal | As | -0.14 | 0.07 | -0.27 | 0.00 |
| BASC-2 BSI | Maternal Prenatal | Cu | 0.17 | 0.08 | 0.02 | 0.32 |
| BASC-2 BSI | Maternal Prenatal | Mn | -0.01 | 0.08 | -0.17 | 0.15 |
| BASC-2 BSI | Maternal Prenatal | Pb | -0.26 | 0.09 | -0.43 | -0.09 |
| BASC-2 BSI | Maternal Prenatal | Se | 0.10 | 0.08 | -0.06 | 0.26 |
| BASC-2 BSI | Maternal Prenatal | Zn | 0.01 | 0.09 | -0.17 | 0.18 |
| BASC-2 BSI | Maternal Postnatal | As | 0.05 | 0.07 | -0.08 | 0.17 |
| BASC-2 BSI | Maternal Postnatal | Cu | -0.11 | 0.07 | -0.25 | 0.03 |
| BASC-2 BSI | Maternal Postnatal | Mn | -0.07 | 0.07 | -0.20 | 0.06 |
| BASC-2 BSI | Maternal Postnatal | Pb | 0.17 | 0.08 | 0.03 | 0.32 |
| BASC-2 BSI | Maternal Postnatal | Se | 0.15 | 0.08 | -0.01 | 0.30 |
| BASC-2 BSI | Maternal Postnatal | Zn | -0.11 | 0.07 | -0.24 | 0.02 |
| BASC-2 BSI | Infant | As | 0.04 | 0.10 | -0.16 | 0.23 |
| BASC-2 BSI | Infant | Cu | -0.07 | 0.08 | -0.22 | 0.08 |
| BASC-2 BSI | Infant | Mn | 0.32 | 0.10 | 0.14 | 0.51 |
| BASC-2 BSI | Infant | Pb | 0.01 | 0.10 | -0.20 | 0.21 |
| BASC-2 BSI | Infant | Se | 0.03 | 0.03 | -0.02 | 0.08 |
| BASC-2 BSI | Infant | Zn | -0.04 | 0.06 | -0.16 | 0.09 |
| BASC-2 EXT | Maternal Prenatal | As | 0.06 | 0.07 | -0.07 | 0.19 |
| BASC-2 EXT | Maternal Prenatal | Cu | 0.12 | 0.08 | -0.02 | 0.27 |
| BASC-2 EXT | Maternal Prenatal | Mn | -0.01 | 0.08 | -0.17 | 0.15 |
| BASC-2 EXT | Maternal Prenatal | Pb | -0.20 | 0.09 | -0.37 | -0.03 |
| BASC-2 EXT | Maternal Prenatal | Se | 0.11 | 0.08 | -0.05 | 0.27 |
| BASC-2 EXT | Maternal Prenatal | Zn | 0.05 | 0.09 | -0.12 | 0.23 |
| BASC-2 EXT | Maternal Postnatal | As | -0.04 | 0.06 | -0.16 | 0.09 |
| BASC-2 EXT | Maternal Postnatal | Cu | -0.17 | 0.07 | -0.31 | -0.03 |
| BASC-2 EXT | Maternal Postnatal | Mn | -0.15 | 0.07 | -0.28 | -0.02 |
| BASC-2 EXT | Maternal Postnatal | Pb | 0.18 | 0.07 | 0.04 | 0.33 |
| BASC-2 EXT | Maternal Postnatal | Se | 0.02 | 0.08 | -0.14 | 0.17 |
| BASC-2 EXT | Maternal Postnatal | Zn | -0.01 | 0.07 | -0.14 | 0.12 |
| BASC-2 EXT | Infant | As | 0.00 | 0.10 | -0.18 | 0.19 |
| BASC-2 EXT | Infant | Cu | 0.00 | 0.07 | -0.14 | 0.15 |
| BASC-2 EXT | Infant | Mn | 0.17 | 0.09 | -0.01 | 0.36 |
| BASC-2 EXT | Infant | Pb | 0.01 | 0.10 | -0.19 | 0.21 |
| BASC-2 EXT | Infant | Se | 0.04 | 0.03 | -0.01 | 0.09 |
| BASC-2 EXT | Infant | Zn | -0.04 | 0.06 | -0.16 | 0.09 |
| BASC-2 INZ | Maternal Prenatal | As | -0.16 | 0.07 | -0.30 | -0.01 |
| BASC-2 INZ | Maternal Prenatal | Cu | 0.15 | 0.09 | -0.01 | 0.32 |
| BASC-2 INZ | Maternal Prenatal | Mn | 0.06 | 0.09 | -0.12 | 0.24 |
| BASC-2 INZ | Maternal Prenatal | Pb | -0.17 | 0.09 | -0.36 | 0.01 |
| BASC-2 INZ | Maternal Prenatal | Se | 0.01 | 0.09 | -0.17 | 0.19 |
| BASC-2 INZ | Maternal Prenatal | Zn | 0.00 | 0.10 | -0.20 | 0.19 |
| BASC-2 INZ | Maternal Postnatal | As | 0.09 | 0.07 | -0.05 | 0.24 |
| BASC-2 INZ | Maternal Postnatal | Cu | -0.13 | 0.08 | -0.29 | 0.03 |
| BASC-2 INZ | Maternal Postnatal | Mn | -0.28 | 0.07 | -0.42 | -0.14 |
| BASC-2 INZ | Maternal Postnatal | Pb | 0.31 | 0.08 | 0.15 | 0.47 |
| BASC-2 INZ | Maternal Postnatal | Se | -0.06 | 0.09 | -0.24 | 0.11 |
| BASC-2 INZ | Maternal Postnatal | Zn | -0.12 | 0.07 | -0.27 | 0.03 |
| BASC-2 INZ | Infant | As | 0.24 | 0.11 | 0.03 | 0.46 |
| BASC-2 INZ | Infant | Cu | 0.01 | 0.08 | -0.15 | 0.17 |
| BASC-2 INZ | Infant | Mn | -0.01 | 0.10 | -0.21 | 0.20 |
| BASC-2 INZ | Infant | Pb | 0.09 | 0.11 | -0.13 | 0.31 |
| BASC-2 INZ | Infant | Se | 0.06 | 0.03 | 0.01 | 0.12 |
| BASC-2 INZ | Infant | Zn | -0.02 | 0.07 | -0.15 | 0.12 |
| BASC-2 AKL | Maternal Prenatal | As | 0.11 | 0.07 | -0.03 | 0.25 |
| BASC-2 AKL | Maternal Prenatal | Cu | -0.09 | 0.08 | -0.25 | 0.07 |
| BASC-2 AKL | Maternal Prenatal | Mn | 0.00 | 0.09 | -0.17 | 0.17 |
| BASC-2 AKL | Maternal Prenatal | Pb | -0.01 | 0.09 | -0.19 | 0.18 |
| BASC-2 AKL | Maternal Prenatal | Se | 0.05 | 0.09 | -0.12 | 0.22 |
| BASC-2 AKL | Maternal Prenatal | Zn | -0.06 | 0.10 | -0.25 | 0.13 |
| BASC-2 AKL | Maternal Postnatal | As | -0.03 | 0.07 | -0.17 | 0.10 |
| BASC-2 AKL | Maternal Postnatal | Cu | -0.04 | 0.08 | -0.18 | 0.11 |
| BASC-2 AKL | Maternal Postnatal | Mn | -0.19 | 0.07 | -0.33 | -0.05 |
| BASC-2 AKL | Maternal Postnatal | Pb | 0.08 | 0.08 | -0.07 | 0.24 |
| BASC-2 AKL | Maternal Postnatal | Se | -0.14 | 0.08 | -0.30 | 0.03 |
| BASC-2 AKL | Maternal Postnatal | Zn | 0.08 | 0.07 | -0.06 | 0.22 |
| BASC-2 AKL | Infant | As | 0.12 | 0.11 | -0.09 | 0.33 |
| BASC-2 AKL | Infant | Cu | 0.07 | 0.08 | -0.09 | 0.23 |
| BASC-2 AKL | Infant | Mn | -0.48 | 0.10 | -0.68 | -0.28 |
| BASC-2 AKL | Infant | Pb | 0.10 | 0.11 | -0.13 | 0.32 |
| BASC-2 AKL | Infant | Se | 0.01 | 0.03 | -0.04 | 0.07 |
| BASC-2 AKL | Infant | Zn | 0.07 | 0.07 | -0.07 | 0.21 |
| Estimate is the difference in the mean predicted outcome (standardized) between the metal fixed at 75% versus 25%, with all other metals fixed at their medians.  Models adjusted for maternal age (quadratic), maternal BMI (quadratic), highest level of parental education (high school or less, any college, any graduate), parity (0, ≥1), smoking status (no second- or first-hand, ever second-hand only, ever first-hand), age at last breastfeeding (<365 days, ≥365 days), maternal marital status (married, other), birthyear (2010-2011, 2012-2013, 2014-2015), Healthy Eating Index (linear), Parenting Relationship Questionnaire (first three principal components), and age at assessment (linear).  Abbreviations: AKL, Adaptive Skills; BASC-2, Behavioral Assessment System for Children, 2^nd^ Ed.; BSI, Behavioral Symptoms Index; CrI, Credible Interval; EXT, Externalizing Problems; INZ, Internalizing Problems; SRS-2, Social Responsiveness Scale, 2^nd^ Ed. | | | | | | |

| Supplemental Table S7. Interactions between metals within time points, among male children. | | | | | | | | |  |
| --- | --- | --- | --- | --- | --- | --- | --- | --- | --- |
| Outcome | Time | Metal 1 | Metal 2 | Estimate | SD | 95% CrI Low | 95% CrI High | | |
| SRS-2 Total | Maternal Prenatal | As | Cu | 0.03 | 0.10 | -0.17 | 0.23 | | |
| SRS-2 Total | Maternal Postnatal | As | Cu | 0.00 | 0.09 | -0.17 | | 0.18 | |
| SRS-2 Total | Infant | As | Cu | -0.34 | 0.16 | -0.67 | | -0.02 | |
| SRS-2 Total | Maternal Prenatal | As | Mn | 0.11 | 0.11 | -0.11 | | 0.33 | |
| SRS-2 Total | Maternal Postnatal | As | Mn | 0.14 | 0.10 | -0.06 | | 0.33 | |
| SRS-2 Total | Infant | As | Mn | -0.18 | 0.18 | -0.54 | | 0.17 | |
| SRS-2 Total | Maternal Prenatal | As | Pb | -0.02 | 0.12 | -0.25 | | 0.21 | |
| SRS-2 Total | Maternal Postnatal | As | Pb | -0.11 | 0.10 | -0.30 | | 0.08 | |
| SRS-2 Total | Infant | As | Pb | 0.27 | 0.18 | -0.09 | | 0.63 | |
| SRS-2 Total | Maternal Prenatal | As | Se | -0.03 | 0.12 | -0.25 | | 0.20 | |
| SRS-2 Total | Maternal Postnatal | As | Se | -0.03 | 0.10 | -0.21 | | 0.16 | |
| SRS-2 Total | Infant | As | Se | 0.25 | 0.15 | -0.04 | | 0.55 | |
| SRS-2 Total | Maternal Prenatal | As | Zn | 0.04 | 0.12 | -0.19 | | 0.27 | |
| SRS-2 Total | Maternal Postnatal | As | Zn | -0.06 | 0.09 | -0.25 | | 0.12 | |
| SRS-2 Total | Infant | As | Zn | -0.20 | 0.16 | -0.51 | | 0.11 | |
| SRS-2 Total | Maternal Prenatal | Cu | As | 0.03 | 0.10 | -0.17 | | 0.23 | |
| SRS-2 Total | Maternal Postnatal | Cu | As | 0.00 | 0.08 | -0.16 | | 0.17 | |
| SRS-2 Total | Infant | Cu | As | -0.34 | 0.13 | -0.60 | | -0.09 | |
| SRS-2 Total | Maternal Prenatal | Cu | Mn | -0.10 | 0.10 | -0.30 | | 0.10 | |
| SRS-2 Total | Maternal Postnatal | Cu | Mn | 0.09 | 0.09 | -0.09 | | 0.26 | |
| SRS-2 Total | Infant | Cu | Mn | 0.06 | 0.12 | -0.18 | | 0.30 | |
| SRS-2 Total | Maternal Prenatal | Cu | Pb | -0.03 | 0.11 | -0.25 | | 0.18 | |
| SRS-2 Total | Maternal Postnatal | Cu | Pb | 0.03 | 0.09 | -0.15 | | 0.21 | |
| SRS-2 Total | Infant | Cu | Pb | -0.20 | 0.12 | -0.44 | | 0.04 | |
| SRS-2 Total | Maternal Prenatal | Cu | Se | 0.00 | 0.10 | -0.20 | | 0.20 | |
| SRS-2 Total | Maternal Postnatal | Cu | Se | -0.04 | 0.09 | -0.21 | | 0.13 | |
| SRS-2 Total | Infant | Cu | Se | -0.05 | 0.10 | -0.25 | | 0.15 | |
| SRS-2 Total | Maternal Prenatal | Cu | Zn | 0.12 | 0.10 | -0.08 | | 0.32 | |
| SRS-2 Total | Maternal Postnatal | Cu | Zn | -0.03 | 0.08 | -0.19 | | 0.13 | |
| SRS-2 Total | Infant | Cu | Zn | 0.14 | 0.11 | -0.07 | | 0.35 | |
| SRS-2 Total | Maternal Prenatal | Mn | As | 0.11 | 0.12 | -0.13 | | 0.34 | |
| SRS-2 Total | Maternal Postnatal | Mn | As | 0.14 | 0.11 | -0.08 | | 0.35 | |
| SRS-2 Total | Infant | Mn | As | -0.18 | 0.18 | -0.53 | | 0.16 | |
| SRS-2 Total | Maternal Prenatal | Mn | Cu | -0.10 | 0.11 | -0.32 | | 0.11 | |
| SRS-2 Total | Maternal Postnatal | Mn | Cu | 0.09 | 0.10 | -0.11 | | 0.28 | |
| SRS-2 Total | Infant | Mn | Cu | 0.06 | 0.15 | -0.23 | | 0.36 | |
| SRS-2 Total | Maternal Prenatal | Mn | Pb | 0.07 | 0.13 | -0.19 | | 0.32 | |
| SRS-2 Total | Maternal Postnatal | Mn | Pb | 0.19 | 0.11 | -0.03 | | 0.42 | |
| SRS-2 Total | Infant | Mn | Pb | -0.17 | 0.15 | -0.47 | | 0.14 | |
| SRS-2 Total | Maternal Prenatal | Mn | Se | 0.14 | 0.13 | -0.11 | | 0.39 | |
| SRS-2 Total | Maternal Postnatal | Mn | Se | 0.02 | 0.11 | -0.20 | | 0.23 | |
| SRS-2 Total | Infant | Mn | Se | -0.08 | 0.14 | -0.36 | | 0.20 | |
| SRS-2 Total | Maternal Prenatal | Mn | Zn | -0.12 | 0.13 | -0.38 | | 0.14 | |
| SRS-2 Total | Maternal Postnatal | Mn | Zn | -0.03 | 0.11 | -0.24 | | 0.18 | |
| SRS-2 Total | Infant | Mn | Zn | 0.13 | 0.15 | -0.16 | | 0.43 | |
| SRS-2 Total | Maternal Prenatal | Pb | As | -0.02 | 0.14 | -0.29 | | 0.25 | |
| SRS-2 Total | Maternal Postnatal | Pb | As | -0.11 | 0.11 | -0.33 | | 0.11 | |
| SRS-2 Total | Infant | Pb | As | 0.27 | 0.18 | -0.08 | | 0.62 | |
| SRS-2 Total | Maternal Prenatal | Pb | Cu | -0.03 | 0.14 | -0.30 | | 0.24 | |
| SRS-2 Total | Maternal Postnatal | Pb | Cu | 0.03 | 0.11 | -0.18 | | 0.24 | |
| SRS-2 Total | Infant | Pb | Cu | -0.20 | 0.15 | -0.49 | | 0.09 | |
| SRS-2 Total | Maternal Prenatal | Pb | Mn | 0.07 | 0.15 | -0.22 | | 0.35 | |
| SRS-2 Total | Maternal Postnatal | Pb | Mn | 0.19 | 0.12 | -0.04 | | 0.43 | |
| SRS-2 Total | Infant | Pb | Mn | -0.17 | 0.15 | -0.46 | | 0.13 | |
| SRS-2 Total | Maternal Prenatal | Pb | Se | -0.01 | 0.15 | -0.30 | | 0.29 | |
| SRS-2 Total | Maternal Postnatal | Pb | Se | -0.05 | 0.11 | -0.27 | | 0.17 | |
| SRS-2 Total | Infant | Pb | Se | -0.02 | 0.14 | -0.29 | | 0.25 | |
| SRS-2 Total | Maternal Prenatal | Pb | Zn | -0.16 | 0.16 | -0.47 | | 0.15 | |
| SRS-2 Total | Maternal Postnatal | Pb | Zn | -0.03 | 0.11 | -0.24 | | 0.19 | |
| SRS-2 Total | Infant | Pb | Zn | 0.07 | 0.15 | -0.21 | | 0.36 | |
| SRS-2 Total | Maternal Prenatal | Se | As | -0.03 | 0.12 | -0.27 | | 0.21 | |
| SRS-2 Total | Maternal Postnatal | Se | As | -0.03 | 0.11 | -0.25 | | 0.20 | |
| SRS-2 Total | Infant | Se | As | 0.25 | 0.07 | 0.12 | | 0.39 | |
| SRS-2 Total | Maternal Prenatal | Se | Cu | 0.00 | 0.11 | -0.22 | | 0.22 | |
| SRS-2 Total | Maternal Postnatal | Se | Cu | -0.04 | 0.11 | -0.25 | | 0.17 | |
| SRS-2 Total | Infant | Se | Cu | -0.05 | 0.05 | -0.14 | | 0.04 | |
| SRS-2 Total | Maternal Prenatal | Se | Mn | 0.14 | 0.13 | -0.11 | | 0.39 | |
| SRS-2 Total | Maternal Postnatal | Se | Mn | 0.02 | 0.12 | -0.21 | | 0.25 | |
| SRS-2 Total | Infant | Se | Mn | -0.08 | 0.06 | -0.20 | | 0.04 | |
| SRS-2 Total | Maternal Prenatal | Se | Pb | -0.01 | 0.14 | -0.28 | | 0.26 | |
| SRS-2 Total | Maternal Postnatal | Se | Pb | -0.05 | 0.12 | -0.28 | | 0.18 | |
| SRS-2 Total | Infant | Se | Pb | -0.02 | 0.06 | -0.14 | | 0.09 | |
| SRS-2 Total | Maternal Prenatal | Se | Zn | -0.01 | 0.13 | -0.25 | | 0.24 | |
| SRS-2 Total | Maternal Postnatal | Se | Zn | -0.08 | 0.11 | -0.29 | | 0.13 | |
| SRS-2 Total | Infant | Se | Zn | -0.04 | 0.04 | -0.12 | | 0.04 | |
| SRS-2 Total | Maternal Prenatal | Zn | As | 0.04 | 0.14 | -0.23 | | 0.31 | |
| SRS-2 Total | Maternal Postnatal | Zn | As | -0.06 | 0.09 | -0.24 | | 0.11 | |
| SRS-2 Total | Infant | Zn | As | -0.20 | 0.11 | -0.41 | | 0.02 | |
| SRS-2 Total | Maternal Prenatal | Zn | Cu | 0.12 | 0.13 | -0.13 | | 0.37 | |
| SRS-2 Total | Maternal Postnatal | Zn | Cu | -0.03 | 0.08 | -0.19 | | 0.13 | |
| SRS-2 Total | Infant | Zn | Cu | 0.14 | 0.10 | -0.05 | | 0.33 | |
| SRS-2 Total | Maternal Prenatal | Zn | Mn | -0.12 | 0.14 | -0.40 | | 0.17 | |
| SRS-2 Total | Maternal Postnatal | Zn | Mn | -0.03 | 0.09 | -0.21 | | 0.16 | |
| SRS-2 Total | Infant | Zn | Mn | 0.13 | 0.11 | -0.08 | | 0.35 | |
| SRS-2 Total | Maternal Prenatal | Zn | Pb | -0.16 | 0.16 | -0.47 | | 0.15 | |
| SRS-2 Total | Maternal Postnatal | Zn | Pb | -0.03 | 0.09 | -0.21 | | 0.16 | |
| SRS-2 Total | Infant | Zn | Pb | 0.07 | 0.11 | -0.14 | | 0.29 | |
| SRS-2 Total | Maternal Prenatal | Zn | Se | -0.01 | 0.14 | -0.28 | | 0.26 | |
| SRS-2 Total | Maternal Postnatal | Zn | Se | -0.08 | 0.09 | -0.25 | | 0.09 | |
| SRS-2 Total | Infant | Zn | Se | -0.04 | 0.09 | -0.21 | | 0.13 | |
| BASC-2 BSI | Maternal Prenatal | As | Cu | -0.01 | 0.10 | -0.22 | | 0.19 | |
| BASC-2 BSI | Maternal Postnatal | As | Cu | -0.06 | 0.10 | -0.26 | | 0.13 | |
| BASC-2 BSI | Infant | As | Cu | -0.35 | 0.16 | -0.67 | | -0.03 | |
| BASC-2 BSI | Maternal Prenatal | As | Mn | 0.12 | 0.11 | -0.10 | | 0.34 | |
| BASC-2 BSI | Maternal Postnatal | As | Mn | -0.01 | 0.11 | -0.22 | | 0.20 | |
| BASC-2 BSI | Infant | As | Mn | 0.05 | 0.17 | -0.29 | | 0.38 | |
| BASC-2 BSI | Maternal Prenatal | As | Pb | -0.06 | 0.12 | -0.29 | | 0.17 | |
| BASC-2 BSI | Maternal Postnatal | As | Pb | -0.11 | 0.11 | -0.32 | | 0.10 | |
| BASC-2 BSI | Infant | As | Pb | 0.07 | 0.18 | -0.29 | | 0.43 | |
| BASC-2 BSI | Maternal Prenatal | As | Se | 0.10 | 0.11 | -0.12 | | 0.33 | |
| BASC-2 BSI | Maternal Postnatal | As | Se | -0.01 | 0.11 | -0.21 | | 0.20 | |
| BASC-2 BSI | Infant | As | Se | 0.02 | 0.15 | -0.27 | | 0.31 | |
| BASC-2 BSI | Maternal Prenatal | As | Zn | -0.12 | 0.12 | -0.35 | | 0.11 | |
| BASC-2 BSI | Maternal Postnatal | As | Zn | 0.04 | 0.10 | -0.16 | | 0.24 | |
| BASC-2 BSI | Infant | As | Zn | -0.05 | 0.15 | -0.35 | | 0.25 | |
| BASC-2 BSI | Maternal Prenatal | Cu | As | -0.01 | 0.12 | -0.25 | | 0.22 | |
| BASC-2 BSI | Maternal Postnatal | Cu | As | -0.06 | 0.11 | -0.28 | | 0.15 | |
| BASC-2 BSI | Infant | Cu | As | -0.35 | 0.14 | -0.63 | | -0.07 | |
| BASC-2 BSI | Maternal Prenatal | Cu | Mn | -0.12 | 0.12 | -0.35 | | 0.11 | |
| BASC-2 BSI | Maternal Postnatal | Cu | Mn | 0.19 | 0.12 | -0.04 | | 0.42 | |
| BASC-2 BSI | Infant | Cu | Mn | 0.09 | 0.13 | -0.17 | | 0.36 | |
| BASC-2 BSI | Maternal Prenatal | Cu | Pb | 0.05 | 0.13 | -0.20 | | 0.30 | |
| BASC-2 BSI | Maternal Postnatal | Cu | Pb | 0.08 | 0.12 | -0.15 | | 0.31 | |
| BASC-2 BSI | Infant | Cu | Pb | -0.11 | 0.15 | -0.39 | | 0.18 | |
| BASC-2 BSI | Maternal Prenatal | Cu | Se | -0.01 | 0.12 | -0.24 | | 0.22 | |
| BASC-2 BSI | Maternal Postnatal | Cu | Se | -0.03 | 0.11 | -0.26 | | 0.19 | |
| BASC-2 BSI | Infant | Cu | Se | 0.01 | 0.11 | -0.21 | | 0.23 | |
| BASC-2 BSI | Maternal Prenatal | Cu | Zn | -0.03 | 0.12 | -0.26 | | 0.21 | |
| BASC-2 BSI | Maternal Postnatal | Cu | Zn | -0.01 | 0.11 | -0.23 | | 0.21 | |
| BASC-2 BSI | Infant | Cu | Zn | 0.12 | 0.12 | -0.12 | | 0.36 | |
| BASC-2 BSI | Maternal Prenatal | Mn | As | 0.12 | 0.13 | -0.13 | | 0.38 | |
| BASC-2 BSI | Maternal Postnatal | Mn | As | -0.01 | 0.11 | -0.22 | | 0.21 | |
| BASC-2 BSI | Infant | Mn | As | 0.05 | 0.17 | -0.29 | | 0.39 | |
| BASC-2 BSI | Maternal Prenatal | Mn | Cu | -0.12 | 0.12 | -0.36 | | 0.12 | |
| BASC-2 BSI | Maternal Postnatal | Mn | Cu | 0.19 | 0.11 | -0.02 | | 0.40 | |
| BASC-2 BSI | Infant | Mn | Cu | 0.09 | 0.15 | -0.21 | | 0.39 | |
| BASC-2 BSI | Maternal Prenatal | Mn | Pb | 0.19 | 0.15 | -0.11 | | 0.48 | |
| BASC-2 BSI | Maternal Postnatal | Mn | Pb | 0.07 | 0.12 | -0.16 | | 0.30 | |
| BASC-2 BSI | Infant | Mn | Pb | -0.51 | 0.17 | -0.85 | | -0.17 | |
| BASC-2 BSI | Maternal Prenatal | Mn | Se | 0.15 | 0.14 | -0.13 | | 0.43 | |
| BASC-2 BSI | Maternal Postnatal | Mn | Se | 0.07 | 0.11 | -0.15 | | 0.29 | |
| BASC-2 BSI | Infant | Mn | Se | -0.08 | 0.15 | -0.36 | | 0.21 | |
| BASC-2 BSI | Maternal Prenatal | Mn | Zn | -0.32 | 0.15 | -0.61 | | -0.04 | |
| BASC-2 BSI | Maternal Postnatal | Mn | Zn | -0.04 | 0.11 | -0.26 | | 0.18 | |
| BASC-2 BSI | Infant | Mn | Zn | 0.22 | 0.15 | -0.07 | | 0.51 | |
| BASC-2 BSI | Maternal Prenatal | Pb | As | -0.06 | 0.14 | -0.33 | | 0.21 | |
| BASC-2 BSI | Maternal Postnatal | Pb | As | -0.11 | 0.12 | -0.34 | | 0.13 | |
| BASC-2 BSI | Infant | Pb | As | 0.07 | 0.19 | -0.31 | | 0.45 | |
| BASC-2 BSI | Maternal Prenatal | Pb | Cu | 0.05 | 0.14 | -0.22 | | 0.32 | |
| BASC-2 BSI | Maternal Postnatal | Pb | Cu | 0.08 | 0.12 | -0.16 | | 0.32 | |
| BASC-2 BSI | Infant | Pb | Cu | -0.11 | 0.18 | -0.45 | | 0.24 | |
| BASC-2 BSI | Maternal Prenatal | Pb | Mn | 0.19 | 0.15 | -0.11 | | 0.49 | |
| BASC-2 BSI | Maternal Postnatal | Pb | Mn | 0.07 | 0.13 | -0.18 | | 0.32 | |
| BASC-2 BSI | Infant | Pb | Mn | -0.51 | 0.18 | -0.86 | | -0.16 | |
| BASC-2 BSI | Maternal Prenatal | Pb | Se | -0.04 | 0.15 | -0.34 | | 0.26 | |
| BASC-2 BSI | Maternal Postnatal | Pb | Se | -0.07 | 0.12 | -0.31 | | 0.17 | |
| BASC-2 BSI | Infant | Pb | Se | 0.01 | 0.16 | -0.29 | | 0.32 | |
| BASC-2 BSI | Maternal Prenatal | Pb | Zn | 0.12 | 0.16 | -0.20 | | 0.44 | |
| BASC-2 BSI | Maternal Postnatal | Pb | Zn | 0.02 | 0.12 | -0.21 | | 0.26 | |
| BASC-2 BSI | Infant | Pb | Zn | 0.04 | 0.16 | -0.29 | | 0.36 | |
| BASC-2 BSI | Maternal Prenatal | Se | As | 0.10 | 0.13 | -0.15 | | 0.36 | |
| BASC-2 BSI | Maternal Postnatal | Se | As | -0.01 | 0.12 | -0.25 | | 0.23 | |
| BASC-2 BSI | Infant | Se | As | 0.02 | 0.06 | -0.10 | | 0.14 | |
| BASC-2 BSI | Maternal Prenatal | Se | Cu | -0.01 | 0.12 | -0.25 | | 0.23 | |
| BASC-2 BSI | Maternal Postnatal | Se | Cu | -0.03 | 0.12 | -0.27 | | 0.20 | |
| BASC-2 BSI | Infant | Se | Cu | 0.01 | 0.05 | -0.08 | | 0.10 | |
| BASC-2 BSI | Maternal Prenatal | Se | Mn | 0.15 | 0.14 | -0.13 | | 0.43 | |
| BASC-2 BSI | Maternal Postnatal | Se | Mn | 0.07 | 0.13 | -0.18 | | 0.32 | |
| BASC-2 BSI | Infant | Se | Mn | -0.08 | 0.06 | -0.19 | | 0.04 | |
| BASC-2 BSI | Maternal Prenatal | Se | Pb | -0.04 | 0.15 | -0.32 | | 0.25 | |
| BASC-2 BSI | Maternal Postnatal | Se | Pb | -0.07 | 0.13 | -0.32 | | 0.18 | |
| BASC-2 BSI | Infant | Se | Pb | 0.01 | 0.06 | -0.10 | | 0.13 | |
| BASC-2 BSI | Maternal Prenatal | Se | Zn | 0.05 | 0.14 | -0.22 | | 0.33 | |
| BASC-2 BSI | Maternal Postnatal | Se | Zn | 0.00 | 0.12 | -0.23 | | 0.23 | |
| BASC-2 BSI | Infant | Se | Zn | -0.03 | 0.04 | -0.11 | | 0.04 | |
| BASC-2 BSI | Maternal Prenatal | Zn | As | -0.12 | 0.15 | -0.41 | | 0.17 | |
| BASC-2 BSI | Maternal Postnatal | Zn | As | 0.04 | 0.10 | -0.16 | | 0.24 | |
| BASC-2 BSI | Infant | Zn | As | -0.05 | 0.11 | -0.26 | | 0.16 | |
| BASC-2 BSI | Maternal Prenatal | Zn | Cu | -0.03 | 0.13 | -0.29 | | 0.24 | |
| BASC-2 BSI | Maternal Postnatal | Zn | Cu | -0.01 | 0.10 | -0.20 | | 0.18 | |
| BASC-2 BSI | Infant | Zn | Cu | 0.12 | 0.11 | -0.09 | | 0.33 | |
| BASC-2 BSI | Maternal Prenatal | Zn | Mn | -0.32 | 0.16 | -0.63 | | -0.02 | |
| BASC-2 BSI | Maternal Postnatal | Zn | Mn | -0.04 | 0.11 | -0.25 | | 0.17 | |
| BASC-2 BSI | Infant | Zn | Mn | 0.22 | 0.11 | 0.00 | | 0.44 | |
| BASC-2 BSI | Maternal Prenatal | Zn | Pb | 0.12 | 0.17 | -0.21 | | 0.45 | |
| BASC-2 BSI | Maternal Postnatal | Zn | Pb | 0.02 | 0.11 | -0.19 | | 0.23 | |
| BASC-2 BSI | Infant | Zn | Pb | 0.04 | 0.12 | -0.19 | | 0.26 | |
| BASC-2 BSI | Maternal Prenatal | Zn | Se | 0.05 | 0.15 | -0.24 | | 0.34 | |
| BASC-2 BSI | Maternal Postnatal | Zn | Se | 0.00 | 0.10 | -0.20 | | 0.19 | |
| BASC-2 BSI | Infant | Zn | Se | -0.03 | 0.09 | -0.21 | | 0.14 | |
| BASC-2 EXT | Maternal Prenatal | As | Cu | -0.02 | 0.10 | -0.22 | | 0.18 | |
| BASC-2 EXT | Maternal Postnatal | As | Cu | -0.06 | 0.10 | -0.25 | | 0.13 | |
| BASC-2 EXT | Infant | As | Cu | -0.18 | 0.16 | -0.49 | | 0.13 | |
| BASC-2 EXT | Maternal Prenatal | As | Mn | 0.10 | 0.11 | -0.11 | | 0.32 | |
| BASC-2 EXT | Maternal Postnatal | As | Mn | 0.10 | 0.11 | -0.11 | | 0.31 | |
| BASC-2 EXT | Infant | As | Mn | -0.34 | 0.17 | -0.66 | | -0.01 | |
| BASC-2 EXT | Maternal Prenatal | As | Pb | -0.14 | 0.11 | -0.36 | | 0.09 | |
| BASC-2 EXT | Maternal Postnatal | As | Pb | -0.09 | 0.11 | -0.29 | | 0.12 | |
| BASC-2 EXT | Infant | As | Pb | 0.10 | 0.18 | -0.25 | | 0.44 | |
| BASC-2 EXT | Maternal Prenatal | As | Se | 0.10 | 0.11 | -0.12 | | 0.32 | |
| BASC-2 EXT | Maternal Postnatal | As | Se | 0.11 | 0.10 | -0.09 | | 0.32 | |
| BASC-2 EXT | Infant | As | Se | -0.11 | 0.15 | -0.40 | | 0.17 | |
| BASC-2 EXT | Maternal Prenatal | As | Zn | -0.04 | 0.12 | -0.27 | | 0.18 | |
| BASC-2 EXT | Maternal Postnatal | As | Zn | -0.03 | 0.10 | -0.23 | | 0.16 | |
| BASC-2 EXT | Infant | As | Zn | 0.10 | 0.15 | -0.19 | | 0.39 | |
| BASC-2 EXT | Maternal Prenatal | Cu | As | -0.02 | 0.12 | -0.25 | | 0.21 | |
| BASC-2 EXT | Maternal Postnatal | Cu | As | -0.06 | 0.11 | -0.27 | | 0.15 | |
| BASC-2 EXT | Infant | Cu | As | -0.18 | 0.14 | -0.45 | | 0.09 | |
| BASC-2 EXT | Maternal Prenatal | Cu | Mn | -0.07 | 0.12 | -0.30 | | 0.16 | |
| BASC-2 EXT | Maternal Postnatal | Cu | Mn | 0.08 | 0.12 | -0.14 | | 0.31 | |
| BASC-2 EXT | Infant | Cu | Mn | 0.17 | 0.13 | -0.08 | | 0.43 | |
| BASC-2 EXT | Maternal Prenatal | Cu | Pb | 0.00 | 0.13 | -0.25 | | 0.24 | |
| BASC-2 EXT | Maternal Postnatal | Cu | Pb | 0.08 | 0.12 | -0.15 | | 0.31 | |
| BASC-2 EXT | Infant | Cu | Pb | 0.00 | 0.14 | -0.27 | | 0.28 | |
| BASC-2 EXT | Maternal Prenatal | Cu | Se | -0.02 | 0.12 | -0.24 | | 0.21 | |
| BASC-2 EXT | Maternal Postnatal | Cu | Se | -0.12 | 0.11 | -0.34 | | 0.10 | |
| BASC-2 EXT | Infant | Cu | Se | -0.02 | 0.11 | -0.23 | | 0.19 | |
| BASC-2 EXT | Maternal Prenatal | Cu | Zn | -0.18 | 0.12 | -0.41 | | 0.05 | |
| BASC-2 EXT | Maternal Postnatal | Cu | Zn | 0.08 | 0.11 | -0.13 | | 0.29 | |
| BASC-2 EXT | Infant | Cu | Zn | -0.06 | 0.12 | -0.30 | | 0.17 | |
| BASC-2 EXT | Maternal Prenatal | Mn | As | 0.10 | 0.13 | -0.15 | | 0.35 | |
| BASC-2 EXT | Maternal Postnatal | Mn | As | 0.10 | 0.11 | -0.11 | | 0.31 | |
| BASC-2 EXT | Infant | Mn | As | -0.34 | 0.17 | -0.67 | | -0.01 | |
| BASC-2 EXT | Maternal Prenatal | Mn | Cu | -0.07 | 0.12 | -0.30 | | 0.17 | |
| BASC-2 EXT | Maternal Postnatal | Mn | Cu | 0.08 | 0.11 | -0.12 | | 0.29 | |
| BASC-2 EXT | Infant | Mn | Cu | 0.17 | 0.15 | -0.12 | | 0.47 | |
| BASC-2 EXT | Maternal Prenatal | Mn | Pb | 0.21 | 0.15 | -0.08 | | 0.50 | |
| BASC-2 EXT | Maternal Postnatal | Mn | Pb | 0.03 | 0.11 | -0.19 | | 0.26 | |
| BASC-2 EXT | Infant | Mn | Pb | -0.35 | 0.17 | -0.68 | | -0.02 | |
| BASC-2 EXT | Maternal Prenatal | Mn | Se | -0.09 | 0.14 | -0.36 | | 0.19 | |
| BASC-2 EXT | Maternal Postnatal | Mn | Se | -0.04 | 0.11 | -0.25 | | 0.18 | |
| BASC-2 EXT | Infant | Mn | Se | -0.01 | 0.14 | -0.28 | | 0.26 | |
| BASC-2 EXT | Maternal Prenatal | Mn | Zn | -0.03 | 0.14 | -0.31 | | 0.26 | |
| BASC-2 EXT | Maternal Postnatal | Mn | Zn | 0.15 | 0.11 | -0.06 | | 0.36 | |
| BASC-2 EXT | Infant | Mn | Zn | 0.02 | 0.15 | -0.27 | | 0.30 | |
| BASC-2 EXT | Maternal Prenatal | Pb | As | -0.14 | 0.14 | -0.40 | | 0.13 | |
| BASC-2 EXT | Maternal Postnatal | Pb | As | -0.09 | 0.12 | -0.32 | | 0.14 | |
| BASC-2 EXT | Infant | Pb | As | 0.10 | 0.18 | -0.26 | | 0.46 | |
| BASC-2 EXT | Maternal Prenatal | Pb | Cu | 0.00 | 0.14 | -0.27 | | 0.26 | |
| BASC-2 EXT | Maternal Postnatal | Pb | Cu | 0.08 | 0.12 | -0.16 | | 0.32 | |
| BASC-2 EXT | Infant | Pb | Cu | 0.00 | 0.17 | -0.33 | | 0.34 | |
| BASC-2 EXT | Maternal Prenatal | Pb | Mn | 0.21 | 0.15 | -0.08 | | 0.51 | |
| BASC-2 EXT | Maternal Postnatal | Pb | Mn | 0.03 | 0.12 | -0.21 | | 0.28 | |
| BASC-2 EXT | Infant | Pb | Mn | -0.35 | 0.18 | -0.70 | | -0.01 | |
| BASC-2 EXT | Maternal Prenatal | Pb | Se | -0.10 | 0.15 | -0.40 | | 0.20 | |
| BASC-2 EXT | Maternal Postnatal | Pb | Se | -0.11 | 0.12 | -0.35 | | 0.13 | |
| BASC-2 EXT | Infant | Pb | Se | 0.10 | 0.15 | -0.19 | | 0.39 | |
| BASC-2 EXT | Maternal Prenatal | Pb | Zn | 0.22 | 0.16 | -0.09 | | 0.54 | |
| BASC-2 EXT | Maternal Postnatal | Pb | Zn | 0.02 | 0.12 | -0.22 | | 0.25 | |
| BASC-2 EXT | Infant | Pb | Zn | 0.09 | 0.16 | -0.22 | | 0.41 | |
| BASC-2 EXT | Maternal Prenatal | Se | As | 0.10 | 0.13 | -0.15 | | 0.35 | |
| BASC-2 EXT | Maternal Postnatal | Se | As | 0.11 | 0.12 | -0.13 | | 0.35 | |
| BASC-2 EXT | Infant | Se | As | -0.11 | 0.06 | -0.23 | | 0.00 | |
| BASC-2 EXT | Maternal Prenatal | Se | Cu | -0.02 | 0.12 | -0.25 | | 0.22 | |
| BASC-2 EXT | Maternal Postnatal | Se | Cu | -0.12 | 0.12 | -0.36 | | 0.11 | |
| BASC-2 EXT | Infant | Se | Cu | -0.02 | 0.04 | -0.10 | | 0.07 | |
| BASC-2 EXT | Maternal Prenatal | Se | Mn | -0.09 | 0.14 | -0.36 | | 0.19 | |
| BASC-2 EXT | Maternal Postnatal | Se | Mn | -0.04 | 0.13 | -0.29 | | 0.21 | |
| BASC-2 EXT | Infant | Se | Mn | -0.01 | 0.06 | -0.13 | | 0.11 | |
| BASC-2 EXT | Maternal Prenatal | Se | Pb | -0.10 | 0.15 | -0.38 | | 0.19 | |
| BASC-2 EXT | Maternal Postnatal | Se | Pb | -0.11 | 0.13 | -0.36 | | 0.13 | |
| BASC-2 EXT | Infant | Se | Pb | 0.10 | 0.06 | -0.01 | | 0.21 | |
| BASC-2 EXT | Maternal Prenatal | Se | Zn | 0.14 | 0.14 | -0.12 | | 0.41 | |
| BASC-2 EXT | Maternal Postnatal | Se | Zn | 0.09 | 0.12 | -0.14 | | 0.33 | |
| BASC-2 EXT | Infant | Se | Zn | 0.01 | 0.04 | -0.07 | | 0.08 | |
| BASC-2 EXT | Maternal Prenatal | Zn | As | -0.04 | 0.14 | -0.32 | | 0.24 | |
| BASC-2 EXT | Maternal Postnatal | Zn | As | -0.03 | 0.10 | -0.24 | | 0.17 | |
| BASC-2 EXT | Infant | Zn | As | 0.10 | 0.10 | -0.11 | | 0.30 | |
| BASC-2 EXT | Maternal Prenatal | Zn | Cu | -0.18 | 0.13 | -0.44 | | 0.08 | |
| BASC-2 EXT | Maternal Postnatal | Zn | Cu | 0.08 | 0.10 | -0.12 | | 0.28 | |
| BASC-2 EXT | Infant | Zn | Cu | -0.06 | 0.10 | -0.27 | | 0.14 | |
| BASC-2 EXT | Maternal Prenatal | Zn | Mn | -0.03 | 0.15 | -0.33 | | 0.28 | |
| BASC-2 EXT | Maternal Postnatal | Zn | Mn | 0.15 | 0.11 | -0.07 | | 0.37 | |
| BASC-2 EXT | Infant | Zn | Mn | 0.02 | 0.11 | -0.20 | | 0.23 | |
| BASC-2 EXT | Maternal Prenatal | Zn | Pb | 0.22 | 0.17 | -0.10 | | 0.55 | |
| BASC-2 EXT | Maternal Postnatal | Zn | Pb | 0.02 | 0.11 | -0.20 | | 0.23 | |
| BASC-2 EXT | Infant | Zn | Pb | 0.09 | 0.11 | -0.13 | | 0.31 | |
| BASC-2 EXT | Maternal Prenatal | Zn | Se | 0.14 | 0.15 | -0.15 | | 0.43 | |
| BASC-2 EXT | Maternal Postnatal | Zn | Se | 0.09 | 0.10 | -0.10 | | 0.29 | |
| BASC-2 EXT | Infant | Zn | Se | 0.01 | 0.09 | -0.17 | | 0.18 | |
| BASC-2 INZ | Maternal Prenatal | As | Cu | 0.03 | 0.11 | -0.19 | | 0.25 | |
| BASC-2 INZ | Maternal Postnatal | As | Cu | 0.03 | 0.11 | -0.19 | | 0.25 | |
| BASC-2 INZ | Infant | As | Cu | -0.42 | 0.18 | -0.77 | | -0.08 | |
| BASC-2 INZ | Maternal Prenatal | As | Mn | 0.13 | 0.12 | -0.10 | | 0.37 | |
| BASC-2 INZ | Maternal Postnatal | As | Mn | -0.07 | 0.12 | -0.31 | | 0.16 | |
| BASC-2 INZ | Infant | As | Mn | -0.26 | 0.18 | -0.63 | | 0.10 | |
| BASC-2 INZ | Maternal Prenatal | As | Pb | -0.23 | 0.13 | -0.48 | | 0.02 | |
| BASC-2 INZ | Maternal Postnatal | As | Pb | -0.13 | 0.12 | -0.37 | | 0.10 | |
| BASC-2 INZ | Infant | As | Pb | 0.16 | 0.20 | -0.22 | | 0.55 | |
| BASC-2 INZ | Maternal Prenatal | As | Se | 0.12 | 0.12 | -0.12 | | 0.36 | |
| BASC-2 INZ | Maternal Postnatal | As | Se | -0.04 | 0.12 | -0.27 | | 0.20 | |
| BASC-2 INZ | Infant | As | Se | -0.12 | 0.16 | -0.43 | | 0.20 | |
| BASC-2 INZ | Maternal Prenatal | As | Zn | -0.20 | 0.13 | -0.46 | | 0.05 | |
| BASC-2 INZ | Maternal Postnatal | As | Zn | 0.17 | 0.11 | -0.05 | | 0.39 | |
| BASC-2 INZ | Infant | As | Zn | 0.07 | 0.16 | -0.25 | | 0.39 | |
| BASC-2 INZ | Maternal Prenatal | Cu | As | 0.03 | 0.13 | -0.23 | | 0.29 | |
| BASC-2 INZ | Maternal Postnatal | Cu | As | 0.03 | 0.12 | -0.21 | | 0.27 | |
| BASC-2 INZ | Infant | Cu | As | -0.42 | 0.15 | -0.72 | | -0.12 | |
| BASC-2 INZ | Maternal Prenatal | Cu | Mn | -0.16 | 0.13 | -0.42 | | 0.10 | |
| BASC-2 INZ | Maternal Postnatal | Cu | Mn | 0.14 | 0.13 | -0.11 | | 0.40 | |
| BASC-2 INZ | Infant | Cu | Mn | -0.06 | 0.14 | -0.34 | | 0.22 | |
| BASC-2 INZ | Maternal Prenatal | Cu | Pb | -0.07 | 0.14 | -0.35 | | 0.21 | |
| BASC-2 INZ | Maternal Postnatal | Cu | Pb | 0.16 | 0.13 | -0.10 | | 0.42 | |
| BASC-2 INZ | Infant | Cu | Pb | 0.23 | 0.16 | -0.08 | | 0.53 | |
| BASC-2 INZ | Maternal Prenatal | Cu | Se | 0.20 | 0.13 | -0.06 | | 0.46 | |
| BASC-2 INZ | Maternal Postnatal | Cu | Se | 0.11 | 0.13 | -0.14 | | 0.36 | |
| BASC-2 INZ | Infant | Cu | Se | -0.04 | 0.12 | -0.27 | | 0.20 | |
| BASC-2 INZ | Maternal Prenatal | Cu | Zn | -0.14 | 0.13 | -0.40 | | 0.11 | |
| BASC-2 INZ | Maternal Postnatal | Cu | Zn | -0.08 | 0.12 | -0.32 | | 0.16 | |
| BASC-2 INZ | Infant | Cu | Zn | 0.04 | 0.13 | -0.22 | | 0.29 | |
| BASC-2 INZ | Maternal Prenatal | Mn | As | 0.13 | 0.14 | -0.14 | | 0.41 | |
| BASC-2 INZ | Maternal Postnatal | Mn | As | -0.07 | 0.12 | -0.31 | | 0.16 | |
| BASC-2 INZ | Infant | Mn | As | -0.26 | 0.19 | -0.63 | | 0.10 | |
| BASC-2 INZ | Maternal Prenatal | Mn | Cu | -0.16 | 0.14 | -0.43 | | 0.10 | |
| BASC-2 INZ | Maternal Postnatal | Mn | Cu | 0.14 | 0.12 | -0.09 | | 0.37 | |
| BASC-2 INZ | Infant | Mn | Cu | -0.06 | 0.16 | -0.38 | | 0.26 | |
| BASC-2 INZ | Maternal Prenatal | Mn | Pb | 0.07 | 0.16 | -0.25 | | 0.40 | |
| BASC-2 INZ | Maternal Postnatal | Mn | Pb | 0.01 | 0.13 | -0.24 | | 0.26 | |
| BASC-2 INZ | Infant | Mn | Pb | -0.02 | 0.19 | -0.38 | | 0.35 | |
| BASC-2 INZ | Maternal Prenatal | Mn | Se | 0.18 | 0.16 | -0.13 | | 0.49 | |
| BASC-2 INZ | Maternal Postnatal | Mn | Se | 0.03 | 0.12 | -0.22 | | 0.27 | |
| BASC-2 INZ | Infant | Mn | Se | -0.02 | 0.16 | -0.33 | | 0.29 | |
| BASC-2 INZ | Maternal Prenatal | Mn | Zn | -0.25 | 0.16 | -0.57 | | 0.06 | |
| BASC-2 INZ | Maternal Postnatal | Mn | Zn | -0.29 | 0.12 | -0.53 | | -0.05 | |
| BASC-2 INZ | Infant | Mn | Zn | 0.13 | 0.16 | -0.19 | | 0.44 | |
| BASC-2 INZ | Maternal Prenatal | Pb | As | -0.23 | 0.15 | -0.53 | | 0.07 | |
| BASC-2 INZ | Maternal Postnatal | Pb | As | -0.13 | 0.13 | -0.39 | | 0.13 | |
| BASC-2 INZ | Infant | Pb | As | 0.16 | 0.20 | -0.24 | | 0.57 | |
| BASC-2 INZ | Maternal Prenatal | Pb | Cu | -0.07 | 0.15 | -0.37 | | 0.23 | |
| BASC-2 INZ | Maternal Postnatal | Pb | Cu | 0.16 | 0.14 | -0.11 | | 0.42 | |
| BASC-2 INZ | Infant | Pb | Cu | 0.23 | 0.19 | -0.14 | | 0.60 | |
| BASC-2 INZ | Maternal Prenatal | Pb | Mn | 0.07 | 0.17 | -0.25 | | 0.40 | |
| BASC-2 INZ | Maternal Postnatal | Pb | Mn | 0.01 | 0.14 | -0.26 | | 0.29 | |
| BASC-2 INZ | Infant | Pb | Mn | -0.02 | 0.19 | -0.39 | | 0.36 | |
| BASC-2 INZ | Maternal Prenatal | Pb | Se | -0.23 | 0.17 | -0.56 | | 0.10 | |
| BASC-2 INZ | Maternal Postnatal | Pb | Se | -0.11 | 0.14 | -0.38 | | 0.16 | |
| BASC-2 INZ | Infant | Pb | Se | 0.10 | 0.16 | -0.22 | | 0.43 | |
| BASC-2 INZ | Maternal Prenatal | Pb | Zn | 0.36 | 0.18 | 0.01 | | 0.71 | |
| BASC-2 INZ | Maternal Postnatal | Pb | Zn | -0.05 | 0.13 | -0.31 | | 0.21 | |
| BASC-2 INZ | Infant | Pb | Zn | -0.05 | 0.18 | -0.40 | | 0.30 | |
| BASC-2 INZ | Maternal Prenatal | Se | As | 0.12 | 0.14 | -0.16 | | 0.40 | |
| BASC-2 INZ | Maternal Postnatal | Se | As | -0.04 | 0.14 | -0.31 | | 0.24 | |
| BASC-2 INZ | Infant | Se | As | -0.12 | 0.07 | -0.25 | | 0.01 | |
| BASC-2 INZ | Maternal Prenatal | Se | Cu | 0.20 | 0.13 | -0.07 | | 0.46 | |
| BASC-2 INZ | Maternal Postnatal | Se | Cu | 0.11 | 0.13 | -0.16 | | 0.37 | |
| BASC-2 INZ | Infant | Se | Cu | -0.04 | 0.05 | -0.13 | | 0.06 | |
| BASC-2 INZ | Maternal Prenatal | Se | Mn | 0.18 | 0.16 | -0.13 | | 0.49 | |
| BASC-2 INZ | Maternal Postnatal | Se | Mn | 0.03 | 0.14 | -0.26 | | 0.31 | |
| BASC-2 INZ | Infant | Se | Mn | -0.02 | 0.06 | -0.15 | | 0.11 | |
| BASC-2 INZ | Maternal Prenatal | Se | Pb | -0.23 | 0.16 | -0.55 | | 0.09 | |
| BASC-2 INZ | Maternal Postnatal | Se | Pb | -0.11 | 0.14 | -0.39 | | 0.17 | |
| BASC-2 INZ | Infant | Se | Pb | 0.10 | 0.06 | -0.02 | | 0.22 | |
| BASC-2 INZ | Maternal Prenatal | Se | Zn | 0.06 | 0.15 | -0.24 | | 0.36 | |
| BASC-2 INZ | Maternal Postnatal | Se | Zn | -0.04 | 0.13 | -0.30 | | 0.22 | |
| BASC-2 INZ | Infant | Se | Zn | -0.04 | 0.04 | -0.12 | | 0.04 | |
| BASC-2 INZ | Maternal Prenatal | Zn | As | -0.20 | 0.16 | -0.52 | | 0.11 | |
| BASC-2 INZ | Maternal Postnatal | Zn | As | 0.17 | 0.12 | -0.06 | | 0.40 | |
| BASC-2 INZ | Infant | Zn | As | 0.07 | 0.12 | -0.16 | | 0.30 | |
| BASC-2 INZ | Maternal Prenatal | Zn | Cu | -0.14 | 0.15 | -0.43 | | 0.14 | |
| BASC-2 INZ | Maternal Postnatal | Zn | Cu | -0.08 | 0.11 | -0.31 | | 0.14 | |
| BASC-2 INZ | Infant | Zn | Cu | 0.04 | 0.11 | -0.19 | | 0.26 | |
| BASC-2 INZ | Maternal Prenatal | Zn | Mn | -0.25 | 0.17 | -0.59 | | 0.09 | |
| BASC-2 INZ | Maternal Postnatal | Zn | Mn | -0.29 | 0.12 | -0.54 | | -0.05 | |
| BASC-2 INZ | Infant | Zn | Mn | 0.13 | 0.12 | -0.11 | | 0.37 | |
| BASC-2 INZ | Maternal Prenatal | Zn | Pb | 0.36 | 0.19 | 0.00 | | 0.72 | |
| BASC-2 INZ | Maternal Postnatal | Zn | Pb | -0.05 | 0.12 | -0.29 | | 0.19 | |
| BASC-2 INZ | Infant | Zn | Pb | -0.05 | 0.12 | -0.29 | | 0.20 | |
| BASC-2 INZ | Maternal Prenatal | Zn | Se | 0.06 | 0.17 | -0.27 | | 0.38 | |
| BASC-2 INZ | Maternal Postnatal | Zn | Se | -0.04 | 0.11 | -0.26 | | 0.18 | |
| BASC-2 INZ | Infant | Zn | Se | -0.04 | 0.10 | -0.23 | | 0.15 | |
| BASC-2 AKL | Maternal Prenatal | As | Cu | 0.00 | 0.11 | -0.22 | | 0.21 | |
| BASC-2 AKL | Maternal Postnatal | As | Cu | 0.01 | 0.10 | -0.20 | | 0.21 | |
| BASC-2 AKL | Infant | As | Cu | 0.28 | 0.18 | -0.07 | | 0.62 | |
| BASC-2 AKL | Maternal Prenatal | As | Mn | 0.12 | 0.12 | -0.11 | | 0.35 | |
| BASC-2 AKL | Maternal Postnatal | As | Mn | -0.04 | 0.11 | -0.26 | | 0.18 | |
| BASC-2 AKL | Infant | As | Mn | -0.18 | 0.19 | -0.55 | | 0.18 | |
| BASC-2 AKL | Maternal Prenatal | As | Pb | -0.10 | 0.12 | -0.34 | | 0.14 | |
| BASC-2 AKL | Maternal Postnatal | As | Pb | 0.09 | 0.11 | -0.13 | | 0.31 | |
| BASC-2 AKL | Infant | As | Pb | -0.13 | 0.20 | -0.52 | | 0.26 | |
| BASC-2 AKL | Maternal Prenatal | As | Se | -0.10 | 0.12 | -0.33 | | 0.13 | |
| BASC-2 AKL | Maternal Postnatal | As | Se | 0.13 | 0.11 | -0.09 | | 0.35 | |
| BASC-2 AKL | Infant | As | Se | -0.22 | 0.16 | -0.53 | | 0.10 | |
| BASC-2 AKL | Maternal Prenatal | As | Zn | -0.08 | 0.12 | -0.32 | | 0.17 | |
| BASC-2 AKL | Maternal Postnatal | As | Zn | 0.00 | 0.11 | -0.21 | | 0.21 | |
| BASC-2 AKL | Infant | As | Zn | 0.09 | 0.17 | -0.23 | | 0.42 | |
| BASC-2 AKL | Maternal Prenatal | Cu | As | 0.00 | 0.13 | -0.25 | | 0.24 | |
| BASC-2 AKL | Maternal Postnatal | Cu | As | 0.01 | 0.11 | -0.22 | | 0.23 | |
| BASC-2 AKL | Infant | Cu | As | 0.28 | 0.15 | -0.03 | | 0.58 | |
| BASC-2 AKL | Maternal Prenatal | Cu | Mn | 0.06 | 0.13 | -0.19 | | 0.31 | |
| BASC-2 AKL | Maternal Postnatal | Cu | Mn | -0.07 | 0.12 | -0.31 | | 0.17 | |
| BASC-2 AKL | Infant | Cu | Mn | -0.08 | 0.14 | -0.36 | | 0.20 | |
| BASC-2 AKL | Maternal Prenatal | Cu | Pb | 0.12 | 0.14 | -0.15 | | 0.38 | |
| BASC-2 AKL | Maternal Postnatal | Cu | Pb | -0.02 | 0.12 | -0.27 | | 0.22 | |
| BASC-2 AKL | Infant | Cu | Pb | 0.21 | 0.16 | -0.10 | | 0.53 | |
| BASC-2 AKL | Maternal Prenatal | Cu | Se | 0.10 | 0.13 | -0.14 | | 0.35 | |
| BASC-2 AKL | Maternal Postnatal | Cu | Se | -0.03 | 0.12 | -0.26 | | 0.21 | |
| BASC-2 AKL | Infant | Cu | Se | -0.05 | 0.12 | -0.28 | | 0.19 | |
| BASC-2 AKL | Maternal Prenatal | Cu | Zn | -0.07 | 0.13 | -0.31 | | 0.18 | |
| BASC-2 AKL | Maternal Postnatal | Cu | Zn | 0.07 | 0.12 | -0.15 | | 0.30 | |
| BASC-2 AKL | Infant | Cu | Zn | -0.21 | 0.13 | -0.47 | | 0.05 | |
| BASC-2 AKL | Maternal Prenatal | Mn | As | 0.12 | 0.14 | -0.15 | | 0.39 | |
| BASC-2 AKL | Maternal Postnatal | Mn | As | -0.04 | 0.12 | -0.27 | | 0.19 | |
| BASC-2 AKL | Infant | Mn | As | -0.18 | 0.19 | -0.55 | | 0.18 | |
| BASC-2 AKL | Maternal Prenatal | Mn | Cu | 0.06 | 0.13 | -0.20 | | 0.32 | |
| BASC-2 AKL | Maternal Postnatal | Mn | Cu | -0.07 | 0.11 | -0.29 | | 0.16 | |
| BASC-2 AKL | Infant | Mn | Cu | -0.08 | 0.17 | -0.40 | | 0.25 | |
| BASC-2 AKL | Maternal Prenatal | Mn | Pb | -0.19 | 0.16 | -0.51 | | 0.12 | |
| BASC-2 AKL | Maternal Postnatal | Mn | Pb | -0.13 | 0.12 | -0.38 | | 0.11 | |
| BASC-2 AKL | Infant | Mn | Pb | 0.57 | 0.19 | 0.20 | | 0.94 | |
| BASC-2 AKL | Maternal Prenatal | Mn | Se | -0.12 | 0.15 | -0.42 | | 0.19 | |
| BASC-2 AKL | Maternal Postnatal | Mn | Se | -0.18 | 0.12 | -0.41 | | 0.06 | |
| BASC-2 AKL | Infant | Mn | Se | 0.11 | 0.16 | -0.19 | | 0.42 | |
| BASC-2 AKL | Maternal Prenatal | Mn | Zn | 0.45 | 0.16 | 0.14 | | 0.76 | |
| BASC-2 AKL | Maternal Postnatal | Mn | Zn | 0.11 | 0.12 | -0.12 | | 0.34 | |
| BASC-2 AKL | Infant | Mn | Zn | -0.24 | 0.16 | -0.56 | | 0.08 | |
| BASC-2 AKL | Maternal Prenatal | Pb | As | -0.10 | 0.15 | -0.39 | | 0.19 | |
| BASC-2 AKL | Maternal Postnatal | Pb | As | 0.09 | 0.13 | -0.16 | | 0.34 | |
| BASC-2 AKL | Infant | Pb | As | -0.13 | 0.21 | -0.54 | | 0.28 | |
| BASC-2 AKL | Maternal Prenatal | Pb | Cu | 0.12 | 0.15 | -0.18 | | 0.41 | |
| BASC-2 AKL | Maternal Postnatal | Pb | Cu | -0.02 | 0.13 | -0.28 | | 0.23 | |
| BASC-2 AKL | Infant | Pb | Cu | 0.21 | 0.19 | -0.16 | | 0.59 | |
| BASC-2 AKL | Maternal Prenatal | Pb | Mn | -0.19 | 0.16 | -0.51 | | 0.13 | |
| BASC-2 AKL | Maternal Postnatal | Pb | Mn | -0.13 | 0.13 | -0.40 | | 0.13 | |
| BASC-2 AKL | Infant | Pb | Mn | 0.57 | 0.20 | 0.18 | | 0.95 | |
| BASC-2 AKL | Maternal Prenatal | Pb | Se | 0.19 | 0.16 | -0.13 | | 0.51 | |
| BASC-2 AKL | Maternal Postnatal | Pb | Se | 0.07 | 0.13 | -0.19 | | 0.32 | |
| BASC-2 AKL | Infant | Pb | Se | 0.14 | 0.17 | -0.19 | | 0.47 | |
| BASC-2 AKL | Maternal Prenatal | Pb | Zn | -0.07 | 0.17 | -0.42 | | 0.27 | |
| BASC-2 AKL | Maternal Postnatal | Pb | Zn | -0.19 | 0.13 | -0.44 | | 0.06 | |
| BASC-2 AKL | Infant | Pb | Zn | -0.04 | 0.18 | -0.39 | | 0.31 | |
| BASC-2 AKL | Maternal Prenatal | Se | As | -0.10 | 0.14 | -0.37 | | 0.17 | |
| BASC-2 AKL | Maternal Postnatal | Se | As | 0.13 | 0.13 | -0.13 | | 0.39 | |
| BASC-2 AKL | Infant | Se | As | -0.22 | 0.07 | -0.35 | | -0.09 | |
| BASC-2 AKL | Maternal Prenatal | Se | Cu | 0.10 | 0.13 | -0.15 | | 0.35 | |
| BASC-2 AKL | Maternal Postnatal | Se | Cu | -0.03 | 0.13 | -0.28 | | 0.22 | |
| BASC-2 AKL | Infant | Se | Cu | -0.05 | 0.05 | -0.15 | | 0.05 | |
| BASC-2 AKL | Maternal Prenatal | Se | Mn | -0.12 | 0.15 | -0.42 | | 0.18 | |
| BASC-2 AKL | Maternal Postnatal | Se | Mn | -0.18 | 0.14 | -0.44 | | 0.09 | |
| BASC-2 AKL | Infant | Se | Mn | 0.11 | 0.07 | -0.02 | | 0.24 | |
| BASC-2 AKL | Maternal Prenatal | Se | Pb | 0.19 | 0.16 | -0.12 | | 0.49 | |
| BASC-2 AKL | Maternal Postnatal | Se | Pb | 0.07 | 0.13 | -0.20 | | 0.33 | |
| BASC-2 AKL | Infant | Se | Pb | 0.14 | 0.06 | 0.01 | | 0.26 | |
| BASC-2 AKL | Maternal Prenatal | Se | Zn | -0.08 | 0.14 | -0.36 | | 0.21 | |
| BASC-2 AKL | Maternal Postnatal | Se | Zn | 0.01 | 0.13 | -0.23 | | 0.26 | |
| BASC-2 AKL | Infant | Se | Zn | 0.06 | 0.04 | -0.03 | | 0.14 | |
| BASC-2 AKL | Maternal Prenatal | Zn | As | -0.08 | 0.16 | -0.38 | | 0.23 | |
| BASC-2 AKL | Maternal Postnatal | Zn | As | 0.00 | 0.11 | -0.22 | | 0.21 | |
| BASC-2 AKL | Infant | Zn | As | 0.09 | 0.12 | -0.13 | | 0.32 | |
| BASC-2 AKL | Maternal Prenatal | Zn | Cu | -0.07 | 0.14 | -0.35 | | 0.21 | |
| BASC-2 AKL | Maternal Postnatal | Zn | Cu | 0.07 | 0.11 | -0.14 | | 0.28 | |
| BASC-2 AKL | Infant | Zn | Cu | -0.21 | 0.12 | -0.44 | | 0.02 | |
| BASC-2 AKL | Maternal Prenatal | Zn | Mn | 0.45 | 0.17 | 0.12 | | 0.78 | |
| BASC-2 AKL | Maternal Postnatal | Zn | Mn | 0.11 | 0.12 | -0.12 | | 0.34 | |
| BASC-2 AKL | Infant | Zn | Mn | -0.24 | 0.12 | -0.48 | | 0.00 | |
| BASC-2 AKL | Maternal Prenatal | Zn | Pb | -0.07 | 0.18 | -0.43 | | 0.28 | |
| BASC-2 AKL | Maternal Postnatal | Zn | Pb | -0.19 | 0.12 | -0.42 | | 0.04 | |
| BASC-2 AKL | Infant | Zn | Pb | -0.04 | 0.13 | -0.28 | | 0.21 | |
| BASC-2 AKL | Maternal Prenatal | Zn | Se | -0.08 | 0.16 | -0.38 | | 0.23 | |
| BASC-2 AKL | Maternal Postnatal | Zn | Se | 0.01 | 0.11 | -0.20 | | 0.23 | |
| BASC-2 AKL | Infant | Zn | Se | 0.06 | 0.10 | -0.14 | | 0.25 | |
| Estimate is the difference in the IQR contrast for metal 1 at 75% of metal 2 versus 25% of metal 2. Estimate = [E(Outcome\|Metal 1 = 75%, Metal 2 = 75%) - E(Outcome\|Metal 1 = 25%, Metal 2 = 75%)] - [E(Outcome\|Metal 1 = 75%, Metal 2 = 25%) - E(Outcome\|Metal 1 = 25%, Metal 2 = 25%)].  Models adjusted for maternal age (quadratic), maternal BMI (quadratic), highest level of parental education (high school or less, any college, any graduate), sex (male, female), parity (0, ≥1), smoking status (no second- or first-hand, ever second-hand only, ever first-hand), age at last breastfeeding (<365 days, ≥365 days), maternal marital status (married, other), birthyear (2010-2011, 2012-2013, 2014-2015), Healthy Eating Index (linear), Parenting Relationship Questionnaire (first three principal components), and age at assessment (linear).  Abbreviations: AKL, Adaptive Skills; BASC-2, Behavioral Assessment System for Children, 2^nd^ Ed.; BSI, Behavioral Symptoms Index; CrI, Credible Interval; EXT, Externalizing Problems; INZ, Internalizing Problems; IQR, Interquartile Range; SRS-2, Social Responsiveness Scale, 2^nd^ Ed. | | | | | | | | |  |

| Supplemental Table S8. Main effects of each metal at each time point, among female children. | | | | | | |
| --- | --- | --- | --- | --- | --- | --- |
| Outcome | Time | Metal | Estimate | SD | 95% CrI Low | 95% CrI High |
| SRS-2 Total | Maternal Prenatal | As | 0.11 | 0.07 | -0.04 | 0.26 |
| SRS-2 Total | Maternal Prenatal | Cu | -0.02 | 0.08 | -0.18 | 0.15 |
| SRS-2 Total | Maternal Prenatal | Mn | -0.12 | 0.08 | -0.28 | 0.04 |
| SRS-2 Total | Maternal Prenatal | Pb | -0.04 | 0.08 | -0.19 | 0.11 |
| SRS-2 Total | Maternal Prenatal | Se | -0.05 | 0.08 | -0.20 | 0.10 |
| SRS-2 Total | Maternal Prenatal | Zn | 0.07 | 0.08 | -0.09 | 0.22 |
| SRS-2 Total | Maternal Postnatal | As | -0.02 | 0.07 | -0.17 | 0.12 |
| SRS-2 Total | Maternal Postnatal | Cu | 0.01 | 0.08 | -0.13 | 0.16 |
| SRS-2 Total | Maternal Postnatal | Mn | -0.01 | 0.08 | -0.16 | 0.14 |
| SRS-2 Total | Maternal Postnatal | Pb | 0.07 | 0.07 | -0.08 | 0.21 |
| SRS-2 Total | Maternal Postnatal | Se | 0.01 | 0.06 | -0.11 | 0.13 |
| SRS-2 Total | Maternal Postnatal | Zn | -0.14 | 0.07 | -0.28 | 0.01 |
| SRS-2 Total | Infant | As | 0.09 | 0.10 | -0.09 | 0.28 |
| SRS-2 Total | Infant | Cu | 0.00 | 0.08 | -0.15 | 0.15 |
| SRS-2 Total | Infant | Mn | -0.08 | 0.07 | -0.21 | 0.06 |
| SRS-2 Total | Infant | Pb | -0.05 | 0.08 | -0.21 | 0.11 |
| SRS-2 Total | Infant | Se | 0.03 | 0.04 | -0.04 | 0.11 |
| SRS-2 Total | Infant | Zn | 0.00 | 0.05 | -0.09 | 0.09 |
| BASC-2 BSI | Maternal Prenatal | As | -0.09 | 0.08 | -0.24 | 0.06 |
| BASC-2 BSI | Maternal Prenatal | Cu | -0.13 | 0.08 | -0.29 | 0.03 |
| BASC-2 BSI | Maternal Prenatal | Mn | 0.02 | 0.09 | -0.15 | 0.19 |
| BASC-2 BSI | Maternal Prenatal | Pb | 0.02 | 0.08 | -0.14 | 0.18 |
| BASC-2 BSI | Maternal Prenatal | Se | 0.13 | 0.08 | -0.02 | 0.28 |
| BASC-2 BSI | Maternal Prenatal | Zn | -0.06 | 0.07 | -0.20 | 0.09 |
| BASC-2 BSI | Maternal Postnatal | As | 0.21 | 0.08 | 0.05 | 0.36 |
| BASC-2 BSI | Maternal Postnatal | Cu | 0.02 | 0.08 | -0.13 | 0.17 |
| BASC-2 BSI | Maternal Postnatal | Mn | -0.16 | 0.08 | -0.32 | 0.00 |
| BASC-2 BSI | Maternal Postnatal | Pb | 0.09 | 0.08 | -0.06 | 0.25 |
| BASC-2 BSI | Maternal Postnatal | Se | -0.12 | 0.07 | -0.26 | 0.02 |
| BASC-2 BSI | Maternal Postnatal | Zn | -0.04 | 0.07 | -0.17 | 0.09 |
| BASC-2 BSI | Infant | As | 0.08 | 0.11 | -0.13 | 0.29 |
| BASC-2 BSI | Infant | Cu | 0.14 | 0.08 | -0.02 | 0.29 |
| BASC-2 BSI | Infant | Mn | 0.11 | 0.07 | -0.03 | 0.25 |
| BASC-2 BSI | Infant | Pb | -0.30 | 0.10 | -0.50 | -0.10 |
| BASC-2 BSI | Infant | Se | 0.08 | 0.04 | 0.00 | 0.17 |
| BASC-2 BSI | Infant | Zn | -0.06 | 0.05 | -0.15 | 0.03 |
| BASC-2 EXT | Maternal Prenatal | As | -0.04 | 0.08 | -0.20 | 0.12 |
| BASC-2 EXT | Maternal Prenatal | Cu | -0.09 | 0.08 | -0.25 | 0.07 |
| BASC-2 EXT | Maternal Prenatal | Mn | -0.10 | 0.09 | -0.28 | 0.07 |
| BASC-2 EXT | Maternal Prenatal | Pb | 0.04 | 0.08 | -0.13 | 0.20 |
| BASC-2 EXT | Maternal Prenatal | Se | 0.17 | 0.08 | 0.02 | 0.32 |
| BASC-2 EXT | Maternal Prenatal | Zn | 0.07 | 0.08 | -0.08 | 0.22 |
| BASC-2 EXT | Maternal Postnatal | As | 0.13 | 0.08 | -0.02 | 0.28 |
| BASC-2 EXT | Maternal Postnatal | Cu | 0.02 | 0.08 | -0.14 | 0.17 |
| BASC-2 EXT | Maternal Postnatal | Mn | -0.16 | 0.08 | -0.33 | 0.00 |
| BASC-2 EXT | Maternal Postnatal | Pb | 0.10 | 0.08 | -0.06 | 0.26 |
| BASC-2 EXT | Maternal Postnatal | Se | -0.11 | 0.07 | -0.25 | 0.03 |
| BASC-2 EXT | Maternal Postnatal | Zn | -0.03 | 0.07 | -0.16 | 0.10 |
| BASC-2 EXT | Infant | As | -0.09 | 0.11 | -0.31 | 0.12 |
| BASC-2 EXT | Infant | Cu | 0.12 | 0.08 | -0.04 | 0.28 |
| BASC-2 EXT | Infant | Mn | 0.14 | 0.07 | 0.00 | 0.28 |
| BASC-2 EXT | Infant | Pb | -0.22 | 0.10 | -0.42 | -0.02 |
| BASC-2 EXT | Infant | Se | 0.10 | 0.04 | 0.01 | 0.19 |
| BASC-2 EXT | Infant | Zn | -0.09 | 0.05 | -0.18 | 0.00 |
| BASC-2 INZ | Maternal Prenatal | As | 0.15 | 0.09 | -0.02 | 0.31 |
| BASC-2 INZ | Maternal Prenatal | Cu | -0.07 | 0.09 | -0.25 | 0.10 |
| BASC-2 INZ | Maternal Prenatal | Mn | 0.15 | 0.10 | -0.04 | 0.34 |
| BASC-2 INZ | Maternal Prenatal | Pb | -0.16 | 0.09 | -0.33 | 0.01 |
| BASC-2 INZ | Maternal Prenatal | Se | 0.05 | 0.08 | -0.11 | 0.22 |
| BASC-2 INZ | Maternal Prenatal | Zn | -0.12 | 0.08 | -0.28 | 0.04 |
| BASC-2 INZ | Maternal Postnatal | As | 0.29 | 0.09 | 0.12 | 0.45 |
| BASC-2 INZ | Maternal Postnatal | Cu | -0.01 | 0.09 | -0.17 | 0.16 |
| BASC-2 INZ | Maternal Postnatal | Mn | -0.09 | 0.09 | -0.27 | 0.09 |
| BASC-2 INZ | Maternal Postnatal | Pb | -0.04 | 0.09 | -0.20 | 0.13 |
| BASC-2 INZ | Maternal Postnatal | Se | -0.09 | 0.08 | -0.24 | 0.06 |
| BASC-2 INZ | Maternal Postnatal | Zn | 0.12 | 0.07 | -0.02 | 0.26 |
| BASC-2 INZ | Infant | As | -0.02 | 0.11 | -0.24 | 0.21 |
| BASC-2 INZ | Infant | Cu | 0.18 | 0.08 | 0.02 | 0.35 |
| BASC-2 INZ | Infant | Mn | -0.04 | 0.07 | -0.19 | 0.10 |
| BASC-2 INZ | Infant | Pb | -0.15 | 0.11 | -0.36 | 0.06 |
| BASC-2 INZ | Infant | Se | 0.01 | 0.05 | -0.08 | 0.10 |
| BASC-2 INZ | Infant | Zn | -0.05 | 0.05 | -0.14 | 0.05 |
| BASC-2 AKL | Maternal Prenatal | As | 0.18 | 0.07 | 0.03 | 0.33 |
| BASC-2 AKL | Maternal Prenatal | Cu | -0.05 | 0.08 | -0.20 | 0.10 |
| BASC-2 AKL | Maternal Prenatal | Mn | 0.04 | 0.08 | -0.12 | 0.20 |
| BASC-2 AKL | Maternal Prenatal | Pb | -0.19 | 0.08 | -0.34 | -0.04 |
| BASC-2 AKL | Maternal Prenatal | Se | -0.12 | 0.07 | -0.27 | 0.02 |
| BASC-2 AKL | Maternal Prenatal | Zn | 0.03 | 0.07 | -0.11 | 0.17 |
| BASC-2 AKL | Maternal Postnatal | As | -0.13 | 0.08 | -0.28 | 0.02 |
| BASC-2 AKL | Maternal Postnatal | Cu | 0.18 | 0.08 | 0.02 | 0.33 |
| BASC-2 AKL | Maternal Postnatal | Mn | -0.05 | 0.08 | -0.21 | 0.11 |
| BASC-2 AKL | Maternal Postnatal | Pb | 0.07 | 0.08 | -0.08 | 0.23 |
| BASC-2 AKL | Maternal Postnatal | Se | 0.07 | 0.07 | -0.07 | 0.21 |
| BASC-2 AKL | Maternal Postnatal | Zn | 0.14 | 0.07 | 0.01 | 0.28 |
| BASC-2 AKL | Infant | As | -0.02 | 0.10 | -0.21 | 0.18 |
| BASC-2 AKL | Infant | Cu | -0.04 | 0.07 | -0.19 | 0.10 |
| BASC-2 AKL | Infant | Mn | 0.05 | 0.06 | -0.08 | 0.18 |
| BASC-2 AKL | Infant | Pb | 0.26 | 0.10 | 0.07 | 0.45 |
| BASC-2 AKL | Infant | Se | -0.07 | 0.04 | -0.15 | 0.01 |
| BASC-2 AKL | Infant | Zn | 0.00 | 0.04 | -0.08 | 0.08 |
| Estimate is the difference in the mean predicted outcome (standardized) between the metal fixed at 75% versus 25%, with all other metals fixed at their medians.  Models adjusted for maternal age (quadratic), maternal BMI (quadratic), highest level of parental education (high school or less, any college, any graduate), parity (0, ≥1), smoking status (no second- or first-hand, ever second-hand only, ever first-hand), age at last breastfeeding (<365 days, ≥365 days), maternal marital status (married, other), birthyear (2010-2011, 2012-2013, 2014-2015), Healthy Eating Index (linear), Parenting Relationship Questionnaire (first three principal components), and age at assessment (linear).  Abbreviations: AKL, Adaptive Skills; BASC-2, Behavioral Assessment System for Children, 2^nd^ Ed.; BSI, Behavioral Symptoms Index; CrI, Credible Interval; EXT, Externalizing Problems; INZ, Internalizing Problems; SRS-2, Social Responsiveness Scale, 2^nd^ Ed. | | | | | | |

| Supplemental Table S9. Interactions between metals within time points, among female children. | | | | | | | | |
| --- | --- | --- | --- | --- | --- | --- | --- | --- |
| Outcome | Time | Metal 1 | Metal 2 | Estimate | SD | 95% CrI Low | | 95% CrI High |
| SRS-2 Total | Maternal Prenatal | As | Cu | -0.20 | 0.12 | -0.43 | 0.03 | |
| SRS-2 Total | Maternal Postnatal | As | Cu | 0.00 | 0.11 | -0.21 | 0.21 | |
| SRS-2 Total | Infant | As | Cu | 0.11 | 0.15 | -0.19 | 0.40 | |
| SRS-2 Total | Maternal Prenatal | As | Mn | 0.06 | 0.12 | -0.19 | 0.30 | |
| SRS-2 Total | Maternal Postnatal | As | Mn | 0.08 | 0.12 | -0.17 | 0.32 | |
| SRS-2 Total | Infant | As | Mn | 0.01 | 0.15 | -0.28 | 0.30 | |
| SRS-2 Total | Maternal Prenatal | As | Pb | 0.25 | 0.12 | 0.02 | 0.48 | |
| SRS-2 Total | Maternal Postnatal | As | Pb | -0.01 | 0.12 | -0.25 | 0.23 | |
| SRS-2 Total | Infant | As | Pb | 0.09 | 0.18 | -0.26 | 0.44 | |
| SRS-2 Total | Maternal Prenatal | As | Se | 0.07 | 0.12 | -0.17 | 0.30 | |
| SRS-2 Total | Maternal Postnatal | As | Se | -0.05 | 0.11 | -0.26 | 0.17 | |
| SRS-2 Total | Infant | As | Se | 0.09 | 0.15 | -0.20 | 0.38 | |
| SRS-2 Total | Maternal Prenatal | As | Zn | 0.08 | 0.12 | -0.16 | 0.32 | |
| SRS-2 Total | Maternal Postnatal | As | Zn | 0.05 | 0.12 | -0.18 | 0.27 | |
| SRS-2 Total | Infant | As | Zn | -0.21 | 0.15 | -0.50 | 0.09 | |
| SRS-2 Total | Maternal Prenatal | Cu | As | -0.20 | 0.13 | -0.45 | 0.05 | |
| SRS-2 Total | Maternal Postnatal | Cu | As | 0.00 | 0.11 | -0.21 | 0.22 | |
| SRS-2 Total | Infant | Cu | As | 0.11 | 0.12 | -0.14 | 0.35 | |
| SRS-2 Total | Maternal Prenatal | Cu | Mn | -0.05 | 0.13 | -0.30 | 0.20 | |
| SRS-2 Total | Maternal Postnatal | Cu | Mn | 0.01 | 0.12 | -0.24 | 0.25 | |
| SRS-2 Total | Infant | Cu | Mn | -0.05 | 0.12 | -0.29 | 0.19 | |
| SRS-2 Total | Maternal Prenatal | Cu | Pb | 0.20 | 0.13 | -0.06 | 0.46 | |
| SRS-2 Total | Maternal Postnatal | Cu | Pb | 0.07 | 0.11 | -0.16 | 0.29 | |
| SRS-2 Total | Infant | Cu | Pb | -0.10 | 0.12 | -0.34 | 0.15 | |
| SRS-2 Total | Maternal Prenatal | Cu | Se | 0.08 | 0.13 | -0.17 | 0.34 | |
| SRS-2 Total | Maternal Postnatal | Cu | Se | 0.01 | 0.11 | -0.21 | 0.23 | |
| SRS-2 Total | Infant | Cu | Se | 0.03 | 0.11 | -0.18 | 0.25 | |
| SRS-2 Total | Maternal Prenatal | Cu | Zn | -0.11 | 0.13 | -0.36 | 0.14 | |
| SRS-2 Total | Maternal Postnatal | Cu | Zn | 0.02 | 0.11 | -0.20 | 0.25 | |
| SRS-2 Total | Infant | Cu | Zn | 0.10 | 0.11 | -0.12 | 0.32 | |
| SRS-2 Total | Maternal Prenatal | Mn | As | 0.06 | 0.13 | -0.20 | 0.32 | |
| SRS-2 Total | Maternal Postnatal | Mn | As | 0.08 | 0.13 | -0.17 | 0.33 | |
| SRS-2 Total | Infant | Mn | As | 0.01 | 0.12 | -0.22 | 0.24 | |
| SRS-2 Total | Maternal Prenatal | Mn | Cu | -0.05 | 0.12 | -0.29 | 0.19 | |
| SRS-2 Total | Maternal Postnatal | Mn | Cu | 0.01 | 0.12 | -0.24 | 0.25 | |
| SRS-2 Total | Infant | Mn | Cu | -0.05 | 0.11 | -0.26 | 0.17 | |
| SRS-2 Total | Maternal Prenatal | Mn | Pb | 0.12 | 0.14 | -0.14 | 0.39 | |
| SRS-2 Total | Maternal Postnatal | Mn | Pb | 0.17 | 0.13 | -0.08 | 0.41 | |
| SRS-2 Total | Infant | Mn | Pb | 0.09 | 0.12 | -0.14 | 0.32 | |
| SRS-2 Total | Maternal Prenatal | Mn | Se | 0.10 | 0.14 | -0.19 | 0.38 | |
| SRS-2 Total | Maternal Postnatal | Mn | Se | -0.08 | 0.13 | -0.33 | 0.17 | |
| SRS-2 Total | Infant | Mn | Se | 0.08 | 0.11 | -0.13 | 0.28 | |
| SRS-2 Total | Maternal Prenatal | Mn | Zn | -0.18 | 0.14 | -0.45 | 0.09 | |
| SRS-2 Total | Maternal Postnatal | Mn | Zn | 0.09 | 0.13 | -0.16 | 0.34 | |
| SRS-2 Total | Infant | Mn | Zn | -0.01 | 0.11 | -0.22 | 0.20 | |
| SRS-2 Total | Maternal Prenatal | Pb | As | 0.25 | 0.12 | 0.01 | 0.49 | |
| SRS-2 Total | Maternal Postnatal | Pb | As | -0.01 | 0.12 | -0.25 | 0.23 | |
| SRS-2 Total | Infant | Pb | As | 0.09 | 0.17 | -0.24 | 0.42 | |
| SRS-2 Total | Maternal Prenatal | Pb | Cu | 0.20 | 0.13 | -0.05 | 0.45 | |
| SRS-2 Total | Maternal Postnatal | Pb | Cu | 0.07 | 0.11 | -0.15 | 0.28 | |
| SRS-2 Total | Infant | Pb | Cu | -0.10 | 0.13 | -0.36 | 0.17 | |
| SRS-2 Total | Maternal Prenatal | Pb | Mn | 0.12 | 0.13 | -0.14 | 0.39 | |
| SRS-2 Total | Maternal Postnatal | Pb | Mn | 0.17 | 0.12 | -0.06 | 0.40 | |
| SRS-2 Total | Infant | Pb | Mn | 0.09 | 0.14 | -0.18 | 0.36 | |
| SRS-2 Total | Maternal Prenatal | Pb | Se | -0.29 | 0.14 | -0.57 | -0.02 | |
| SRS-2 Total | Maternal Postnatal | Pb | Se | -0.08 | 0.12 | -0.31 | 0.15 | |
| SRS-2 Total | Infant | Pb | Se | -0.12 | 0.13 | -0.37 | 0.13 | |
| SRS-2 Total | Maternal Prenatal | Pb | Zn | -0.06 | 0.13 | -0.32 | 0.20 | |
| SRS-2 Total | Maternal Postnatal | Pb | Zn | 0.02 | 0.12 | -0.22 | 0.26 | |
| SRS-2 Total | Infant | Pb | Zn | 0.03 | 0.12 | -0.22 | 0.27 | |
| SRS-2 Total | Maternal Prenatal | Se | As | 0.07 | 0.12 | -0.17 | 0.31 | |
| SRS-2 Total | Maternal Postnatal | Se | As | -0.05 | 0.09 | -0.23 | 0.14 | |
| SRS-2 Total | Infant | Se | As | 0.09 | 0.08 | -0.07 | 0.25 | |
| SRS-2 Total | Maternal Prenatal | Se | Cu | 0.08 | 0.12 | -0.16 | 0.32 | |
| SRS-2 Total | Maternal Postnatal | Se | Cu | 0.01 | 0.09 | -0.17 | 0.20 | |
| SRS-2 Total | Infant | Se | Cu | 0.03 | 0.06 | -0.09 | 0.16 | |
| SRS-2 Total | Maternal Prenatal | Se | Mn | 0.10 | 0.14 | -0.17 | 0.37 | |
| SRS-2 Total | Maternal Postnatal | Se | Mn | -0.08 | 0.11 | -0.30 | 0.13 | |
| SRS-2 Total | Infant | Se | Mn | 0.08 | 0.07 | -0.05 | 0.21 | |
| SRS-2 Total | Maternal Prenatal | Se | Pb | -0.29 | 0.13 | -0.56 | -0.03 | |
| SRS-2 Total | Maternal Postnatal | Se | Pb | -0.08 | 0.10 | -0.28 | 0.12 | |
| SRS-2 Total | Infant | Se | Pb | -0.12 | 0.07 | -0.25 | 0.01 | |
| SRS-2 Total | Maternal Prenatal | Se | Zn | 0.09 | 0.13 | -0.16 | 0.34 | |
| SRS-2 Total | Maternal Postnatal | Se | Zn | -0.05 | 0.09 | -0.23 | 0.14 | |
| SRS-2 Total | Infant | Se | Zn | -0.01 | 0.06 | -0.13 | 0.11 | |
| SRS-2 Total | Maternal Prenatal | Zn | As | 0.08 | 0.13 | -0.17 | 0.34 | |
| SRS-2 Total | Maternal Postnatal | Zn | As | 0.05 | 0.12 | -0.18 | 0.27 | |
| SRS-2 Total | Infant | Zn | As | -0.21 | 0.10 | -0.40 | -0.02 | |
| SRS-2 Total | Maternal Prenatal | Zn | Cu | -0.11 | 0.13 | -0.36 | 0.13 | |
| SRS-2 Total | Maternal Postnatal | Zn | Cu | 0.02 | 0.11 | -0.19 | 0.24 | |
| SRS-2 Total | Infant | Zn | Cu | 0.10 | 0.08 | -0.05 | 0.25 | |
| SRS-2 Total | Maternal Prenatal | Zn | Mn | -0.18 | 0.13 | -0.45 | 0.08 | |
| SRS-2 Total | Maternal Postnatal | Zn | Mn | 0.09 | 0.12 | -0.16 | 0.33 | |
| SRS-2 Total | Infant | Zn | Mn | -0.01 | 0.08 | -0.18 | 0.15 | |
| SRS-2 Total | Maternal Prenatal | Zn | Pb | -0.06 | 0.13 | -0.32 | 0.20 | |
| SRS-2 Total | Maternal Postnatal | Zn | Pb | 0.02 | 0.12 | -0.22 | 0.26 | |
| SRS-2 Total | Infant | Zn | Pb | 0.03 | 0.08 | -0.13 | 0.19 | |
| SRS-2 Total | Maternal Prenatal | Zn | Se | 0.09 | 0.13 | -0.17 | 0.35 | |
| SRS-2 Total | Maternal Postnatal | Zn | Se | -0.05 | 0.11 | -0.26 | 0.17 | |
| SRS-2 Total | Infant | Zn | Se | -0.01 | 0.07 | -0.14 | 0.13 | |
| BASC-2 BSI | Maternal Prenatal | As | Cu | -0.01 | 0.12 | -0.25 | 0.22 | |
| BASC-2 BSI | Maternal Postnatal | As | Cu | 0.02 | 0.11 | -0.20 | 0.24 | |
| BASC-2 BSI | Infant | As | Cu | 0.07 | 0.20 | -0.32 | 0.47 | |
| BASC-2 BSI | Maternal Prenatal | As | Mn | -0.29 | 0.13 | -0.55 | -0.03 | |
| BASC-2 BSI | Maternal Postnatal | As | Mn | 0.04 | 0.13 | -0.22 | 0.31 | |
| BASC-2 BSI | Infant | As | Mn | -0.08 | 0.17 | -0.41 | 0.24 | |
| BASC-2 BSI | Maternal Prenatal | As | Pb | 0.25 | 0.13 | 0.00 | 0.49 | |
| BASC-2 BSI | Maternal Postnatal | As | Pb | -0.03 | 0.13 | -0.29 | 0.23 | |
| BASC-2 BSI | Infant | As | Pb | 0.22 | 0.22 | -0.22 | 0.66 | |
| BASC-2 BSI | Maternal Prenatal | As | Se | 0.04 | 0.13 | -0.21 | 0.28 | |
| BASC-2 BSI | Maternal Postnatal | As | Se | 0.02 | 0.12 | -0.21 | 0.25 | |
| BASC-2 BSI | Infant | As | Se | 0.14 | 0.17 | -0.19 | 0.47 | |
| BASC-2 BSI | Maternal Prenatal | As | Zn | 0.00 | 0.13 | -0.25 | 0.25 | |
| BASC-2 BSI | Maternal Postnatal | As | Zn | -0.02 | 0.12 | -0.25 | 0.22 | |
| BASC-2 BSI | Infant | As | Zn | -0.14 | 0.17 | -0.47 | 0.18 | |
| BASC-2 BSI | Maternal Prenatal | Cu | As | -0.01 | 0.12 | -0.26 | 0.23 | |
| BASC-2 BSI | Maternal Postnatal | Cu | As | 0.02 | 0.12 | -0.20 | 0.25 | |
| BASC-2 BSI | Infant | Cu | As | 0.07 | 0.17 | -0.26 | 0.41 | |
| BASC-2 BSI | Maternal Prenatal | Cu | Mn | -0.01 | 0.12 | -0.25 | 0.24 | |
| BASC-2 BSI | Maternal Postnatal | Cu | Mn | 0.18 | 0.13 | -0.07 | 0.44 | |
| BASC-2 BSI | Infant | Cu | Mn | -0.10 | 0.13 | -0.36 | 0.17 | |
| BASC-2 BSI | Maternal Prenatal | Cu | Pb | -0.14 | 0.13 | -0.40 | 0.11 | |
| BASC-2 BSI | Maternal Postnatal | Cu | Pb | 0.07 | 0.12 | -0.16 | 0.30 | |
| BASC-2 BSI | Infant | Cu | Pb | -0.12 | 0.14 | -0.39 | 0.15 | |
| BASC-2 BSI | Maternal Prenatal | Cu | Se | -0.03 | 0.13 | -0.28 | 0.23 | |
| BASC-2 BSI | Maternal Postnatal | Cu | Se | 0.02 | 0.12 | -0.21 | 0.25 | |
| BASC-2 BSI | Infant | Cu | Se | -0.05 | 0.11 | -0.27 | 0.18 | |
| BASC-2 BSI | Maternal Prenatal | Cu | Zn | 0.05 | 0.12 | -0.19 | 0.30 | |
| BASC-2 BSI | Maternal Postnatal | Cu | Zn | -0.01 | 0.11 | -0.24 | 0.21 | |
| BASC-2 BSI | Infant | Cu | Zn | 0.12 | 0.12 | -0.11 | 0.35 | |
| BASC-2 BSI | Maternal Prenatal | Mn | As | -0.29 | 0.14 | -0.56 | -0.02 | |
| BASC-2 BSI | Maternal Postnatal | Mn | As | 0.04 | 0.14 | -0.23 | 0.32 | |
| BASC-2 BSI | Infant | Mn | As | -0.08 | 0.12 | -0.32 | 0.16 | |
| BASC-2 BSI | Maternal Prenatal | Mn | Cu | -0.01 | 0.13 | -0.26 | 0.25 | |
| BASC-2 BSI | Maternal Postnatal | Mn | Cu | 0.18 | 0.13 | -0.08 | 0.45 | |
| BASC-2 BSI | Infant | Mn | Cu | -0.10 | 0.12 | -0.33 | 0.14 | |
| BASC-2 BSI | Maternal Prenatal | Mn | Pb | 0.07 | 0.14 | -0.21 | 0.36 | |
| BASC-2 BSI | Maternal Postnatal | Mn | Pb | 0.06 | 0.14 | -0.21 | 0.32 | |
| BASC-2 BSI | Infant | Mn | Pb | 0.31 | 0.13 | 0.05 | 0.56 | |
| BASC-2 BSI | Maternal Prenatal | Mn | Se | -0.28 | 0.15 | -0.58 | 0.02 | |
| BASC-2 BSI | Maternal Postnatal | Mn | Se | -0.06 | 0.14 | -0.33 | 0.21 | |
| BASC-2 BSI | Infant | Mn | Se | 0.02 | 0.11 | -0.19 | 0.23 | |
| BASC-2 BSI | Maternal Prenatal | Mn | Zn | 0.10 | 0.14 | -0.18 | 0.38 | |
| BASC-2 BSI | Maternal Postnatal | Mn | Zn | -0.09 | 0.13 | -0.35 | 0.17 | |
| BASC-2 BSI | Infant | Mn | Zn | -0.12 | 0.11 | -0.33 | 0.10 | |
| BASC-2 BSI | Maternal Prenatal | Pb | As | 0.25 | 0.13 | 0.00 | 0.49 | |
| BASC-2 BSI | Maternal Postnatal | Pb | As | -0.03 | 0.14 | -0.30 | 0.23 | |
| BASC-2 BSI | Infant | Pb | As | 0.22 | 0.22 | -0.21 | 0.65 | |
| BASC-2 BSI | Maternal Prenatal | Pb | Cu | -0.14 | 0.13 | -0.40 | 0.11 | |
| BASC-2 BSI | Maternal Postnatal | Pb | Cu | 0.07 | 0.12 | -0.17 | 0.31 | |
| BASC-2 BSI | Infant | Pb | Cu | -0.12 | 0.16 | -0.44 | 0.20 | |
| BASC-2 BSI | Maternal Prenatal | Pb | Mn | 0.07 | 0.14 | -0.20 | 0.35 | |
| BASC-2 BSI | Maternal Postnatal | Pb | Mn | 0.06 | 0.13 | -0.19 | 0.31 | |
| BASC-2 BSI | Infant | Pb | Mn | 0.31 | 0.17 | -0.02 | 0.64 | |
| BASC-2 BSI | Maternal Prenatal | Pb | Se | 0.18 | 0.14 | -0.10 | 0.46 | |
| BASC-2 BSI | Maternal Postnatal | Pb | Se | -0.16 | 0.13 | -0.42 | 0.09 | |
| BASC-2 BSI | Infant | Pb | Se | -0.04 | 0.15 | -0.34 | 0.26 | |
| BASC-2 BSI | Maternal Prenatal | Pb | Zn | -0.14 | 0.13 | -0.41 | 0.12 | |
| BASC-2 BSI | Maternal Postnatal | Pb | Zn | 0.05 | 0.13 | -0.20 | 0.30 | |
| BASC-2 BSI | Infant | Pb | Zn | 0.06 | 0.15 | -0.23 | 0.35 | |
| BASC-2 BSI | Maternal Prenatal | Se | As | 0.04 | 0.13 | -0.21 | 0.29 | |
| BASC-2 BSI | Maternal Postnatal | Se | As | 0.02 | 0.11 | -0.19 | 0.23 | |
| BASC-2 BSI | Infant | Se | As | 0.14 | 0.09 | -0.04 | 0.32 | |
| BASC-2 BSI | Maternal Prenatal | Se | Cu | -0.03 | 0.13 | -0.28 | 0.22 | |
| BASC-2 BSI | Maternal Postnatal | Se | Cu | 0.02 | 0.11 | -0.19 | 0.23 | |
| BASC-2 BSI | Infant | Se | Cu | -0.05 | 0.07 | -0.18 | 0.09 | |
| BASC-2 BSI | Maternal Prenatal | Se | Mn | -0.28 | 0.14 | -0.56 | 0.00 | |
| BASC-2 BSI | Maternal Postnatal | Se | Mn | -0.06 | 0.12 | -0.30 | 0.18 | |
| BASC-2 BSI | Infant | Se | Mn | 0.02 | 0.08 | -0.13 | 0.17 | |
| BASC-2 BSI | Maternal Prenatal | Se | Pb | 0.18 | 0.14 | -0.09 | 0.45 | |
| BASC-2 BSI | Maternal Postnatal | Se | Pb | -0.16 | 0.12 | -0.40 | 0.07 | |
| BASC-2 BSI | Infant | Se | Pb | -0.04 | 0.08 | -0.19 | 0.11 | |
| BASC-2 BSI | Maternal Prenatal | Se | Zn | 0.25 | 0.13 | 0.00 | 0.50 | |
| BASC-2 BSI | Maternal Postnatal | Se | Zn | 0.02 | 0.10 | -0.18 | 0.23 | |
| BASC-2 BSI | Infant | Se | Zn | -0.02 | 0.07 | -0.15 | 0.11 | |
| BASC-2 BSI | Maternal Prenatal | Zn | As | 0.00 | 0.12 | -0.23 | 0.24 | |
| BASC-2 BSI | Maternal Postnatal | Zn | As | -0.02 | 0.10 | -0.22 | 0.19 | |
| BASC-2 BSI | Infant | Zn | As | -0.14 | 0.11 | -0.35 | 0.06 | |
| BASC-2 BSI | Maternal Prenatal | Zn | Cu | 0.05 | 0.11 | -0.17 | 0.27 | |
| BASC-2 BSI | Maternal Postnatal | Zn | Cu | -0.01 | 0.10 | -0.21 | 0.18 | |
| BASC-2 BSI | Infant | Zn | Cu | 0.12 | 0.08 | -0.03 | 0.27 | |
| BASC-2 BSI | Maternal Prenatal | Zn | Mn | 0.10 | 0.13 | -0.15 | 0.35 | |
| BASC-2 BSI | Maternal Postnatal | Zn | Mn | -0.09 | 0.11 | -0.31 | 0.13 | |
| BASC-2 BSI | Infant | Zn | Mn | -0.12 | 0.09 | -0.29 | 0.05 | |
| BASC-2 BSI | Maternal Prenatal | Zn | Pb | -0.14 | 0.13 | -0.39 | 0.11 | |
| BASC-2 BSI | Maternal Postnatal | Zn | Pb | 0.05 | 0.11 | -0.17 | 0.27 | |
| BASC-2 BSI | Infant | Zn | Pb | 0.06 | 0.08 | -0.10 | 0.22 | |
| BASC-2 BSI | Maternal Prenatal | Zn | Se | 0.25 | 0.12 | 0.01 | 0.50 | |
| BASC-2 BSI | Maternal Postnatal | Zn | Se | 0.02 | 0.10 | -0.17 | 0.22 | |
| BASC-2 BSI | Infant | Zn | Se | -0.02 | 0.07 | -0.15 | 0.12 | |
| BASC-2 EXT | Maternal Prenatal | As | Cu | -0.01 | 0.12 | -0.25 | 0.23 | |
| BASC-2 EXT | Maternal Postnatal | As | Cu | 0.02 | 0.11 | -0.20 | 0.24 | |
| BASC-2 EXT | Infant | As | Cu | -0.06 | 0.21 | -0.47 | 0.35 | |
| BASC-2 EXT | Maternal Prenatal | As | Mn | -0.35 | 0.14 | -0.62 | -0.09 | |
| BASC-2 EXT | Maternal Postnatal | As | Mn | 0.07 | 0.14 | -0.19 | 0.34 | |
| BASC-2 EXT | Infant | As | Mn | -0.15 | 0.17 | -0.48 | 0.19 | |
| BASC-2 EXT | Maternal Prenatal | As | Pb | 0.33 | 0.13 | 0.08 | 0.58 | |
| BASC-2 EXT | Maternal Postnatal | As | Pb | -0.16 | 0.13 | -0.42 | 0.10 | |
| BASC-2 EXT | Infant | As | Pb | 0.30 | 0.23 | -0.16 | 0.76 | |
| BASC-2 EXT | Maternal Prenatal | As | Se | 0.12 | 0.13 | -0.14 | 0.37 | |
| BASC-2 EXT | Maternal Postnatal | As | Se | 0.07 | 0.12 | -0.16 | 0.30 | |
| BASC-2 EXT | Infant | As | Se | 0.26 | 0.17 | -0.08 | 0.59 | |
| BASC-2 EXT | Maternal Prenatal | As | Zn | -0.01 | 0.13 | -0.26 | 0.24 | |
| BASC-2 EXT | Maternal Postnatal | As | Zn | -0.02 | 0.12 | -0.25 | 0.21 | |
| BASC-2 EXT | Infant | As | Zn | 0.01 | 0.17 | -0.33 | 0.35 | |
| BASC-2 EXT | Maternal Prenatal | Cu | As | -0.01 | 0.13 | -0.26 | 0.24 | |
| BASC-2 EXT | Maternal Postnatal | Cu | As | 0.02 | 0.12 | -0.21 | 0.25 | |
| BASC-2 EXT | Infant | Cu | As | -0.06 | 0.18 | -0.41 | 0.29 | |
| BASC-2 EXT | Maternal Prenatal | Cu | Mn | 0.06 | 0.13 | -0.19 | 0.31 | |
| BASC-2 EXT | Maternal Postnatal | Cu | Mn | 0.15 | 0.13 | -0.10 | 0.41 | |
| BASC-2 EXT | Infant | Cu | Mn | -0.11 | 0.14 | -0.38 | 0.16 | |
| BASC-2 EXT | Maternal Prenatal | Cu | Pb | -0.13 | 0.13 | -0.39 | 0.13 | |
| BASC-2 EXT | Maternal Postnatal | Cu | Pb | 0.05 | 0.12 | -0.18 | 0.29 | |
| BASC-2 EXT | Infant | Cu | Pb | -0.13 | 0.14 | -0.41 | 0.15 | |
| BASC-2 EXT | Maternal Prenatal | Cu | Se | -0.09 | 0.13 | -0.35 | 0.17 | |
| BASC-2 EXT | Maternal Postnatal | Cu | Se | -0.03 | 0.12 | -0.26 | 0.21 | |
| BASC-2 EXT | Infant | Cu | Se | -0.04 | 0.12 | -0.27 | 0.19 | |
| BASC-2 EXT | Maternal Prenatal | Cu | Zn | 0.03 | 0.13 | -0.22 | 0.28 | |
| BASC-2 EXT | Maternal Postnatal | Cu | Zn | -0.02 | 0.12 | -0.25 | 0.20 | |
| BASC-2 EXT | Infant | Cu | Zn | 0.11 | 0.12 | -0.14 | 0.35 | |
| BASC-2 EXT | Maternal Prenatal | Mn | As | -0.35 | 0.14 | -0.63 | -0.07 | |
| BASC-2 EXT | Maternal Postnatal | Mn | As | 0.07 | 0.14 | -0.20 | 0.35 | |
| BASC-2 EXT | Infant | Mn | As | -0.15 | 0.13 | -0.40 | 0.10 | |
| BASC-2 EXT | Maternal Prenatal | Mn | Cu | 0.06 | 0.13 | -0.20 | 0.32 | |
| BASC-2 EXT | Maternal Postnatal | Mn | Cu | 0.15 | 0.14 | -0.12 | 0.42 | |
| BASC-2 EXT | Infant | Mn | Cu | -0.11 | 0.12 | -0.35 | 0.13 | |
| BASC-2 EXT | Maternal Prenatal | Mn | Pb | -0.06 | 0.15 | -0.35 | 0.23 | |
| BASC-2 EXT | Maternal Postnatal | Mn | Pb | -0.04 | 0.14 | -0.31 | 0.23 | |
| BASC-2 EXT | Infant | Mn | Pb | 0.29 | 0.13 | 0.02 | 0.55 | |
| BASC-2 EXT | Maternal Prenatal | Mn | Se | -0.23 | 0.16 | -0.54 | 0.08 | |
| BASC-2 EXT | Maternal Postnatal | Mn | Se | -0.04 | 0.14 | -0.32 | 0.23 | |
| BASC-2 EXT | Infant | Mn | Se | 0.01 | 0.11 | -0.21 | 0.23 | |
| BASC-2 EXT | Maternal Prenatal | Mn | Zn | -0.04 | 0.15 | -0.32 | 0.25 | |
| BASC-2 EXT | Maternal Postnatal | Mn | Zn | -0.06 | 0.13 | -0.32 | 0.20 | |
| BASC-2 EXT | Infant | Mn | Zn | -0.05 | 0.11 | -0.28 | 0.17 | |
| BASC-2 EXT | Maternal Prenatal | Pb | As | 0.33 | 0.13 | 0.08 | 0.59 | |
| BASC-2 EXT | Maternal Postnatal | Pb | As | -0.16 | 0.14 | -0.43 | 0.11 | |
| BASC-2 EXT | Infant | Pb | As | 0.30 | 0.23 | -0.15 | 0.74 | |
| BASC-2 EXT | Maternal Prenatal | Pb | Cu | -0.13 | 0.13 | -0.40 | 0.13 | |
| BASC-2 EXT | Maternal Postnatal | Pb | Cu | 0.05 | 0.12 | -0.19 | 0.29 | |
| BASC-2 EXT | Infant | Pb | Cu | -0.13 | 0.17 | -0.46 | 0.20 | |
| BASC-2 EXT | Maternal Prenatal | Pb | Mn | -0.06 | 0.14 | -0.34 | 0.22 | |
| BASC-2 EXT | Maternal Postnatal | Pb | Mn | -0.04 | 0.13 | -0.29 | 0.22 | |
| BASC-2 EXT | Infant | Pb | Mn | 0.29 | 0.17 | -0.05 | 0.63 | |
| BASC-2 EXT | Maternal Prenatal | Pb | Se | 0.05 | 0.15 | -0.24 | 0.34 | |
| BASC-2 EXT | Maternal Postnatal | Pb | Se | -0.15 | 0.13 | -0.41 | 0.11 | |
| BASC-2 EXT | Infant | Pb | Se | 0.03 | 0.16 | -0.28 | 0.34 | |
| BASC-2 EXT | Maternal Prenatal | Pb | Zn | -0.08 | 0.14 | -0.35 | 0.19 | |
| BASC-2 EXT | Maternal Postnatal | Pb | Zn | 0.03 | 0.13 | -0.23 | 0.28 | |
| BASC-2 EXT | Infant | Pb | Zn | -0.10 | 0.15 | -0.40 | 0.20 | |
| BASC-2 EXT | Maternal Prenatal | Se | As | 0.12 | 0.13 | -0.14 | 0.37 | |
| BASC-2 EXT | Maternal Postnatal | Se | As | 0.07 | 0.11 | -0.14 | 0.28 | |
| BASC-2 EXT | Infant | Se | As | 0.26 | 0.10 | 0.06 | 0.45 | |
| BASC-2 EXT | Maternal Prenatal | Se | Cu | -0.09 | 0.13 | -0.34 | 0.16 | |
| BASC-2 EXT | Maternal Postnatal | Se | Cu | -0.03 | 0.11 | -0.24 | 0.18 | |
| BASC-2 EXT | Infant | Se | Cu | -0.04 | 0.07 | -0.18 | 0.10 | |
| BASC-2 EXT | Maternal Prenatal | Se | Mn | -0.23 | 0.15 | -0.52 | 0.06 | |
| BASC-2 EXT | Maternal Postnatal | Se | Mn | -0.04 | 0.12 | -0.28 | 0.20 | |
| BASC-2 EXT | Infant | Se | Mn | 0.01 | 0.08 | -0.14 | 0.16 | |
| BASC-2 EXT | Maternal Prenatal | Se | Pb | 0.05 | 0.14 | -0.23 | 0.33 | |
| BASC-2 EXT | Maternal Postnatal | Se | Pb | -0.15 | 0.12 | -0.38 | 0.09 | |
| BASC-2 EXT | Infant | Se | Pb | 0.03 | 0.08 | -0.13 | 0.19 | |
| BASC-2 EXT | Maternal Prenatal | Se | Zn | 0.25 | 0.13 | -0.01 | 0.50 | |
| BASC-2 EXT | Maternal Postnatal | Se | Zn | 0.02 | 0.10 | -0.18 | 0.23 | |
| BASC-2 EXT | Infant | Se | Zn | -0.03 | 0.07 | -0.16 | 0.11 | |
| BASC-2 EXT | Maternal Prenatal | Zn | As | -0.01 | 0.12 | -0.25 | 0.23 | |
| BASC-2 EXT | Maternal Postnatal | Zn | As | -0.02 | 0.10 | -0.22 | 0.18 | |
| BASC-2 EXT | Infant | Zn | As | 0.01 | 0.11 | -0.20 | 0.23 | |
| BASC-2 EXT | Maternal Prenatal | Zn | Cu | 0.03 | 0.12 | -0.20 | 0.26 | |
| BASC-2 EXT | Maternal Postnatal | Zn | Cu | -0.02 | 0.10 | -0.22 | 0.17 | |
| BASC-2 EXT | Infant | Zn | Cu | 0.11 | 0.08 | -0.05 | 0.26 | |
| BASC-2 EXT | Maternal Prenatal | Zn | Mn | -0.04 | 0.13 | -0.29 | 0.22 | |
| BASC-2 EXT | Maternal Postnatal | Zn | Mn | -0.06 | 0.11 | -0.28 | 0.16 | |
| BASC-2 EXT | Infant | Zn | Mn | -0.05 | 0.09 | -0.23 | 0.12 | |
| BASC-2 EXT | Maternal Prenatal | Zn | Pb | -0.08 | 0.13 | -0.33 | 0.18 | |
| BASC-2 EXT | Maternal Postnatal | Zn | Pb | 0.03 | 0.11 | -0.19 | 0.25 | |
| BASC-2 EXT | Infant | Zn | Pb | -0.10 | 0.09 | -0.26 | 0.07 | |
| BASC-2 EXT | Maternal Prenatal | Zn | Se | 0.25 | 0.13 | 0.00 | 0.50 | |
| BASC-2 EXT | Maternal Postnatal | Zn | Se | 0.02 | 0.10 | -0.17 | 0.21 | |
| BASC-2 EXT | Infant | Zn | Se | -0.03 | 0.07 | -0.17 | 0.11 | |
| BASC-2 INZ | Maternal Prenatal | As | Cu | -0.04 | 0.13 | -0.30 | 0.21 | |
| BASC-2 INZ | Maternal Postnatal | As | Cu | -0.15 | 0.12 | -0.40 | 0.09 | |
| BASC-2 INZ | Infant | As | Cu | 0.21 | 0.21 | -0.21 | 0.63 | |
| BASC-2 INZ | Maternal Prenatal | As | Mn | -0.40 | 0.15 | -0.69 | -0.11 | |
| BASC-2 INZ | Maternal Postnatal | As | Mn | -0.11 | 0.15 | -0.40 | 0.18 | |
| BASC-2 INZ | Infant | As | Mn | 0.10 | 0.18 | -0.25 | 0.44 | |
| BASC-2 INZ | Maternal Prenatal | As | Pb | 0.55 | 0.14 | 0.28 | 0.82 | |
| BASC-2 INZ | Maternal Postnatal | As | Pb | 0.03 | 0.15 | -0.25 | 0.32 | |
| BASC-2 INZ | Infant | As | Pb | 0.25 | 0.24 | -0.21 | 0.72 | |
| BASC-2 INZ | Maternal Prenatal | As | Se | 0.25 | 0.14 | -0.02 | 0.52 | |
| BASC-2 INZ | Maternal Postnatal | As | Se | -0.02 | 0.13 | -0.28 | 0.23 | |
| BASC-2 INZ | Infant | As | Se | -0.12 | 0.18 | -0.47 | 0.22 | |
| BASC-2 INZ | Maternal Prenatal | As | Zn | -0.13 | 0.14 | -0.40 | 0.14 | |
| BASC-2 INZ | Maternal Postnatal | As | Zn | 0.10 | 0.13 | -0.16 | 0.36 | |
| BASC-2 INZ | Infant | As | Zn | 0.00 | 0.18 | -0.35 | 0.35 | |
| BASC-2 INZ | Maternal Prenatal | Cu | As | -0.04 | 0.14 | -0.31 | 0.23 | |
| BASC-2 INZ | Maternal Postnatal | Cu | As | -0.15 | 0.13 | -0.40 | 0.09 | |
| BASC-2 INZ | Infant | Cu | As | 0.21 | 0.18 | -0.15 | 0.56 | |
| BASC-2 INZ | Maternal Prenatal | Cu | Mn | -0.09 | 0.14 | -0.36 | 0.17 | |
| BASC-2 INZ | Maternal Postnatal | Cu | Mn | 0.17 | 0.14 | -0.11 | 0.44 | |
| BASC-2 INZ | Infant | Cu | Mn | -0.07 | 0.14 | -0.34 | 0.21 | |
| BASC-2 INZ | Maternal Prenatal | Cu | Pb | 0.09 | 0.14 | -0.20 | 0.37 | |
| BASC-2 INZ | Maternal Postnatal | Cu | Pb | -0.02 | 0.13 | -0.28 | 0.24 | |
| BASC-2 INZ | Infant | Cu | Pb | 0.02 | 0.15 | -0.27 | 0.30 | |
| BASC-2 INZ | Maternal Prenatal | Cu | Se | 0.05 | 0.14 | -0.23 | 0.33 | |
| BASC-2 INZ | Maternal Postnatal | Cu | Se | 0.18 | 0.13 | -0.07 | 0.43 | |
| BASC-2 INZ | Infant | Cu | Se | -0.17 | 0.12 | -0.41 | 0.06 | |
| BASC-2 INZ | Maternal Prenatal | Cu | Zn | -0.01 | 0.14 | -0.28 | 0.26 | |
| BASC-2 INZ | Maternal Postnatal | Cu | Zn | -0.12 | 0.13 | -0.37 | 0.13 | |
| BASC-2 INZ | Infant | Cu | Zn | 0.01 | 0.13 | -0.24 | 0.25 | |
| BASC-2 INZ | Maternal Prenatal | Mn | As | -0.40 | 0.15 | -0.70 | -0.10 | |
| BASC-2 INZ | Maternal Postnatal | Mn | As | -0.11 | 0.15 | -0.41 | 0.19 | |
| BASC-2 INZ | Infant | Mn | As | 0.10 | 0.13 | -0.16 | 0.35 | |
| BASC-2 INZ | Maternal Prenatal | Mn | Cu | -0.09 | 0.14 | -0.37 | 0.19 | |
| BASC-2 INZ | Maternal Postnatal | Mn | Cu | 0.17 | 0.15 | -0.12 | 0.45 | |
| BASC-2 INZ | Infant | Mn | Cu | -0.07 | 0.12 | -0.31 | 0.17 | |
| BASC-2 INZ | Maternal Prenatal | Mn | Pb | 0.18 | 0.16 | -0.12 | 0.49 | |
| BASC-2 INZ | Maternal Postnatal | Mn | Pb | 0.10 | 0.15 | -0.18 | 0.39 | |
| BASC-2 INZ | Infant | Mn | Pb | -0.07 | 0.14 | -0.33 | 0.20 | |
| BASC-2 INZ | Maternal Prenatal | Mn | Se | -0.44 | 0.17 | -0.77 | -0.10 | |
| BASC-2 INZ | Maternal Postnatal | Mn | Se | 0.04 | 0.15 | -0.26 | 0.34 | |
| BASC-2 INZ | Infant | Mn | Se | 0.07 | 0.12 | -0.16 | 0.29 | |
| BASC-2 INZ | Maternal Prenatal | Mn | Zn | 0.22 | 0.16 | -0.09 | 0.52 | |
| BASC-2 INZ | Maternal Postnatal | Mn | Zn | -0.22 | 0.14 | -0.51 | 0.06 | |
| BASC-2 INZ | Infant | Mn | Zn | -0.12 | 0.12 | -0.35 | 0.11 | |
| BASC-2 INZ | Maternal Prenatal | Pb | As | 0.55 | 0.14 | 0.28 | 0.82 | |
| BASC-2 INZ | Maternal Postnatal | Pb | As | 0.03 | 0.15 | -0.26 | 0.32 | |
| BASC-2 INZ | Infant | Pb | As | 0.25 | 0.23 | -0.20 | 0.71 | |
| BASC-2 INZ | Maternal Prenatal | Pb | Cu | 0.09 | 0.14 | -0.19 | 0.37 | |
| BASC-2 INZ | Maternal Postnatal | Pb | Cu | -0.02 | 0.13 | -0.28 | 0.24 | |
| BASC-2 INZ | Infant | Pb | Cu | 0.02 | 0.17 | -0.32 | 0.36 | |
| BASC-2 INZ | Maternal Prenatal | Pb | Mn | 0.18 | 0.15 | -0.11 | 0.48 | |
| BASC-2 INZ | Maternal Postnatal | Pb | Mn | 0.10 | 0.14 | -0.17 | 0.37 | |
| BASC-2 INZ | Infant | Pb | Mn | -0.07 | 0.18 | -0.41 | 0.28 | |
| BASC-2 INZ | Maternal Prenatal | Pb | Se | -0.04 | 0.15 | -0.34 | 0.27 | |
| BASC-2 INZ | Maternal Postnatal | Pb | Se | -0.11 | 0.14 | -0.39 | 0.17 | |
| BASC-2 INZ | Infant | Pb | Se | -0.03 | 0.16 | -0.34 | 0.29 | |
| BASC-2 INZ | Maternal Prenatal | Pb | Zn | 0.22 | 0.15 | -0.07 | 0.51 | |
| BASC-2 INZ | Maternal Postnatal | Pb | Zn | 0.00 | 0.14 | -0.27 | 0.28 | |
| BASC-2 INZ | Infant | Pb | Zn | 0.19 | 0.16 | -0.12 | 0.50 | |
| BASC-2 INZ | Maternal Prenatal | Se | As | 0.25 | 0.14 | -0.03 | 0.52 | |
| BASC-2 INZ | Maternal Postnatal | Se | As | -0.02 | 0.12 | -0.26 | 0.22 | |
| BASC-2 INZ | Infant | Se | As | -0.12 | 0.10 | -0.32 | 0.07 | |
| BASC-2 INZ | Maternal Prenatal | Se | Cu | 0.05 | 0.14 | -0.22 | 0.32 | |
| BASC-2 INZ | Maternal Postnatal | Se | Cu | 0.18 | 0.12 | -0.05 | 0.41 | |
| BASC-2 INZ | Infant | Se | Cu | -0.17 | 0.07 | -0.32 | -0.03 | |
| BASC-2 INZ | Maternal Prenatal | Se | Mn | -0.44 | 0.16 | -0.74 | -0.13 | |
| BASC-2 INZ | Maternal Postnatal | Se | Mn | 0.04 | 0.14 | -0.23 | 0.31 | |
| BASC-2 INZ | Infant | Se | Mn | 0.07 | 0.08 | -0.09 | 0.22 | |
| BASC-2 INZ | Maternal Prenatal | Se | Pb | -0.04 | 0.15 | -0.34 | 0.26 | |
| BASC-2 INZ | Maternal Postnatal | Se | Pb | -0.11 | 0.13 | -0.37 | 0.15 | |
| BASC-2 INZ | Infant | Se | Pb | -0.03 | 0.08 | -0.19 | 0.13 | |
| BASC-2 INZ | Maternal Prenatal | Se | Zn | 0.41 | 0.14 | 0.13 | 0.68 | |
| BASC-2 INZ | Maternal Postnatal | Se | Zn | 0.06 | 0.12 | -0.17 | 0.28 | |
| BASC-2 INZ | Infant | Se | Zn | 0.04 | 0.07 | -0.10 | 0.18 | |
| BASC-2 INZ | Maternal Prenatal | Zn | As | -0.13 | 0.13 | -0.38 | 0.13 | |
| BASC-2 INZ | Maternal Postnatal | Zn | As | 0.10 | 0.11 | -0.12 | 0.33 | |
| BASC-2 INZ | Infant | Zn | As | 0.00 | 0.11 | -0.22 | 0.22 | |
| BASC-2 INZ | Maternal Prenatal | Zn | Cu | -0.01 | 0.13 | -0.26 | 0.24 | |
| BASC-2 INZ | Maternal Postnatal | Zn | Cu | -0.12 | 0.11 | -0.34 | 0.09 | |
| BASC-2 INZ | Infant | Zn | Cu | 0.01 | 0.08 | -0.15 | 0.16 | |
| BASC-2 INZ | Maternal Prenatal | Zn | Mn | 0.22 | 0.14 | -0.06 | 0.49 | |
| BASC-2 INZ | Maternal Postnatal | Zn | Mn | -0.22 | 0.12 | -0.47 | 0.02 | |
| BASC-2 INZ | Infant | Zn | Mn | -0.12 | 0.09 | -0.30 | 0.06 | |
| BASC-2 INZ | Maternal Prenatal | Zn | Pb | 0.22 | 0.14 | -0.05 | 0.50 | |
| BASC-2 INZ | Maternal Postnatal | Zn | Pb | 0.00 | 0.13 | -0.24 | 0.25 | |
| BASC-2 INZ | Infant | Zn | Pb | 0.19 | 0.09 | 0.02 | 0.36 | |
| BASC-2 INZ | Maternal Prenatal | Zn | Se | 0.41 | 0.14 | 0.14 | 0.67 | |
| BASC-2 INZ | Maternal Postnatal | Zn | Se | 0.06 | 0.11 | -0.16 | 0.27 | |
| BASC-2 INZ | Infant | Zn | Se | 0.04 | 0.07 | -0.10 | 0.18 | |
| BASC-2 AKL | Maternal Prenatal | As | Cu | 0.14 | 0.11 | -0.09 | 0.36 | |
| BASC-2 AKL | Maternal Postnatal | As | Cu | -0.06 | 0.11 | -0.28 | 0.16 | |
| BASC-2 AKL | Infant | As | Cu | -0.15 | 0.19 | -0.53 | 0.22 | |
| BASC-2 AKL | Maternal Prenatal | As | Mn | 0.25 | 0.13 | 0.00 | 0.50 | |
| BASC-2 AKL | Maternal Postnatal | As | Mn | -0.05 | 0.14 | -0.31 | 0.22 | |
| BASC-2 AKL | Infant | As | Mn | 0.11 | 0.16 | -0.20 | 0.42 | |
| BASC-2 AKL | Maternal Prenatal | As | Pb | -0.09 | 0.12 | -0.32 | 0.15 | |
| BASC-2 AKL | Maternal Postnatal | As | Pb | -0.18 | 0.13 | -0.44 | 0.08 | |
| BASC-2 AKL | Infant | As | Pb | -0.16 | 0.22 | -0.58 | 0.26 | |
| BASC-2 AKL | Maternal Prenatal | As | Se | 0.15 | 0.12 | -0.09 | 0.38 | |
| BASC-2 AKL | Maternal Postnatal | As | Se | 0.26 | 0.12 | 0.02 | 0.50 | |
| BASC-2 AKL | Infant | As | Se | -0.07 | 0.15 | -0.37 | 0.23 | |
| BASC-2 AKL | Maternal Prenatal | As | Zn | -0.15 | 0.12 | -0.39 | 0.08 | |
| BASC-2 AKL | Maternal Postnatal | As | Zn | -0.07 | 0.12 | -0.31 | 0.16 | |
| BASC-2 AKL | Infant | As | Zn | 0.45 | 0.16 | 0.15 | 0.76 | |
| BASC-2 AKL | Maternal Prenatal | Cu | As | 0.14 | 0.12 | -0.10 | 0.37 | |
| BASC-2 AKL | Maternal Postnatal | Cu | As | -0.06 | 0.12 | -0.29 | 0.17 | |
| BASC-2 AKL | Infant | Cu | As | -0.15 | 0.16 | -0.48 | 0.17 | |
| BASC-2 AKL | Maternal Prenatal | Cu | Mn | -0.02 | 0.12 | -0.25 | 0.22 | |
| BASC-2 AKL | Maternal Postnatal | Cu | Mn | -0.02 | 0.13 | -0.28 | 0.23 | |
| BASC-2 AKL | Infant | Cu | Mn | -0.03 | 0.13 | -0.28 | 0.21 | |
| BASC-2 AKL | Maternal Prenatal | Cu | Pb | 0.01 | 0.13 | -0.23 | 0.26 | |
| BASC-2 AKL | Maternal Postnatal | Cu | Pb | -0.14 | 0.12 | -0.37 | 0.09 | |
| BASC-2 AKL | Infant | Cu | Pb | 0.19 | 0.13 | -0.07 | 0.45 | |
| BASC-2 AKL | Maternal Prenatal | Cu | Se | -0.04 | 0.12 | -0.28 | 0.20 | |
| BASC-2 AKL | Maternal Postnatal | Cu | Se | 0.02 | 0.12 | -0.22 | 0.25 | |
| BASC-2 AKL | Infant | Cu | Se | -0.04 | 0.11 | -0.25 | 0.18 | |
| BASC-2 AKL | Maternal Prenatal | Cu | Zn | -0.07 | 0.12 | -0.30 | 0.16 | |
| BASC-2 AKL | Maternal Postnatal | Cu | Zn | 0.02 | 0.12 | -0.21 | 0.25 | |
| BASC-2 AKL | Infant | Cu | Zn | -0.02 | 0.11 | -0.24 | 0.21 | |
| BASC-2 AKL | Maternal Prenatal | Mn | As | 0.25 | 0.13 | -0.01 | 0.51 | |
| BASC-2 AKL | Maternal Postnatal | Mn | As | -0.05 | 0.14 | -0.32 | 0.23 | |
| BASC-2 AKL | Infant | Mn | As | 0.11 | 0.12 | -0.12 | 0.34 | |
| BASC-2 AKL | Maternal Prenatal | Mn | Cu | -0.02 | 0.12 | -0.26 | 0.23 | |
| BASC-2 AKL | Maternal Postnatal | Mn | Cu | -0.02 | 0.13 | -0.28 | 0.23 | |
| BASC-2 AKL | Infant | Mn | Cu | -0.03 | 0.11 | -0.25 | 0.18 | |
| BASC-2 AKL | Maternal Prenatal | Mn | Pb | -0.09 | 0.14 | -0.36 | 0.18 | |
| BASC-2 AKL | Maternal Postnatal | Mn | Pb | -0.23 | 0.13 | -0.49 | 0.02 | |
| BASC-2 AKL | Infant | Mn | Pb | -0.30 | 0.12 | -0.53 | -0.06 | |
| BASC-2 AKL | Maternal Prenatal | Mn | Se | 0.11 | 0.15 | -0.17 | 0.40 | |
| BASC-2 AKL | Maternal Postnatal | Mn | Se | -0.10 | 0.14 | -0.37 | 0.17 | |
| BASC-2 AKL | Infant | Mn | Se | 0.09 | 0.10 | -0.11 | 0.30 | |
| BASC-2 AKL | Maternal Prenatal | Mn | Zn | -0.01 | 0.13 | -0.28 | 0.25 | |
| BASC-2 AKL | Maternal Postnatal | Mn | Zn | 0.09 | 0.13 | -0.16 | 0.35 | |
| BASC-2 AKL | Infant | Mn | Zn | -0.03 | 0.10 | -0.23 | 0.17 | |
| BASC-2 AKL | Maternal Prenatal | Pb | As | -0.09 | 0.12 | -0.32 | 0.15 | |
| BASC-2 AKL | Maternal Postnatal | Pb | As | -0.18 | 0.13 | -0.45 | 0.08 | |
| BASC-2 AKL | Infant | Pb | As | -0.16 | 0.21 | -0.58 | 0.26 | |
| BASC-2 AKL | Maternal Prenatal | Pb | Cu | 0.01 | 0.12 | -0.23 | 0.25 | |
| BASC-2 AKL | Maternal Postnatal | Pb | Cu | -0.14 | 0.12 | -0.37 | 0.09 | |
| BASC-2 AKL | Infant | Pb | Cu | 0.19 | 0.16 | -0.12 | 0.50 | |
| BASC-2 AKL | Maternal Prenatal | Pb | Mn | -0.09 | 0.13 | -0.34 | 0.17 | |
| BASC-2 AKL | Maternal Postnatal | Pb | Mn | -0.23 | 0.12 | -0.47 | 0.01 | |
| BASC-2 AKL | Infant | Pb | Mn | -0.30 | 0.16 | -0.61 | 0.02 | |
| BASC-2 AKL | Maternal Prenatal | Pb | Se | 0.13 | 0.13 | -0.13 | 0.39 | |
| BASC-2 AKL | Maternal Postnatal | Pb | Se | 0.25 | 0.13 | 0.00 | 0.50 | |
| BASC-2 AKL | Infant | Pb | Se | 0.17 | 0.15 | -0.12 | 0.46 | |
| BASC-2 AKL | Maternal Prenatal | Pb | Zn | 0.23 | 0.13 | -0.02 | 0.48 | |
| BASC-2 AKL | Maternal Postnatal | Pb | Zn | -0.25 | 0.13 | -0.50 | 0.00 | |
| BASC-2 AKL | Infant | Pb | Zn | -0.17 | 0.14 | -0.45 | 0.11 | |
| BASC-2 AKL | Maternal Prenatal | Se | As | 0.15 | 0.12 | -0.09 | 0.38 | |
| BASC-2 AKL | Maternal Postnatal | Se | As | 0.26 | 0.11 | 0.04 | 0.48 | |
| BASC-2 AKL | Infant | Se | As | -0.07 | 0.09 | -0.24 | 0.11 | |
| BASC-2 AKL | Maternal Prenatal | Se | Cu | -0.04 | 0.12 | -0.28 | 0.19 | |
| BASC-2 AKL | Maternal Postnatal | Se | Cu | 0.02 | 0.11 | -0.20 | 0.23 | |
| BASC-2 AKL | Infant | Se | Cu | -0.04 | 0.07 | -0.17 | 0.09 | |
| BASC-2 AKL | Maternal Prenatal | Se | Mn | 0.11 | 0.14 | -0.16 | 0.38 | |
| BASC-2 AKL | Maternal Postnatal | Se | Mn | -0.10 | 0.13 | -0.35 | 0.15 | |
| BASC-2 AKL | Infant | Se | Mn | 0.09 | 0.07 | -0.05 | 0.24 | |
| BASC-2 AKL | Maternal Prenatal | Se | Pb | 0.13 | 0.13 | -0.13 | 0.39 | |
| BASC-2 AKL | Maternal Postnatal | Se | Pb | 0.25 | 0.12 | 0.01 | 0.49 | |
| BASC-2 AKL | Infant | Se | Pb | 0.17 | 0.07 | 0.03 | 0.31 | |
| BASC-2 AKL | Maternal Prenatal | Se | Zn | 0.01 | 0.12 | -0.23 | 0.25 | |
| BASC-2 AKL | Maternal Postnatal | Se | Zn | -0.07 | 0.11 | -0.28 | 0.14 | |
| BASC-2 AKL | Infant | Se | Zn | -0.12 | 0.06 | -0.24 | 0.01 | |
| BASC-2 AKL | Maternal Prenatal | Zn | As | -0.15 | 0.11 | -0.38 | 0.07 | |
| BASC-2 AKL | Maternal Postnatal | Zn | As | -0.07 | 0.11 | -0.29 | 0.14 | |
| BASC-2 AKL | Infant | Zn | As | 0.45 | 0.10 | 0.26 | 0.65 | |
| BASC-2 AKL | Maternal Prenatal | Zn | Cu | -0.07 | 0.11 | -0.29 | 0.15 | |
| BASC-2 AKL | Maternal Postnatal | Zn | Cu | 0.02 | 0.10 | -0.18 | 0.22 | |
| BASC-2 AKL | Infant | Zn | Cu | -0.02 | 0.07 | -0.15 | 0.12 | |
| BASC-2 AKL | Maternal Prenatal | Zn | Mn | -0.01 | 0.12 | -0.25 | 0.22 | |
| BASC-2 AKL | Maternal Postnatal | Zn | Mn | 0.09 | 0.12 | -0.13 | 0.32 | |
| BASC-2 AKL | Infant | Zn | Mn | -0.03 | 0.08 | -0.19 | 0.13 | |
| BASC-2 AKL | Maternal Prenatal | Zn | Pb | 0.23 | 0.12 | -0.01 | 0.47 | |
| BASC-2 AKL | Maternal Postnatal | Zn | Pb | -0.25 | 0.12 | -0.48 | -0.02 | |
| BASC-2 AKL | Infant | Zn | Pb | -0.17 | 0.08 | -0.32 | -0.01 | |
| BASC-2 AKL | Maternal Prenatal | Zn | Se | 0.01 | 0.12 | -0.22 | 0.24 | |
| BASC-2 AKL | Maternal Postnatal | Zn | Se | -0.07 | 0.10 | -0.28 | 0.13 | |
| BASC-2 AKL | Infant | Zn | Se | -0.12 | 0.06 | -0.25 | 0.01 | |
| Estimate is the difference in the IQR contrast for metal 1 at 75% of metal 2 versus 25% of metal 2. Estimate = [E(Outcome\|Metal 1 = 75%, Metal 2 = 75%) - E(Outcome\|Metal 1 = 25%, Metal 2 = 75%)] - [E(Outcome\|Metal 1 = 75%, Metal 2 = 25%) - E(Outcome\|Metal 1 = 25%, Metal 2 = 25%)].  Models adjusted for maternal age (quadratic), maternal BMI (quadratic), highest level of parental education (high school or less, any college, any graduate), sex (male, female), parity (0, ≥1), smoking status (no second- or first-hand, ever second-hand only, ever first-hand), age at last breastfeeding (<365 days, ≥365 days), maternal marital status (married, other), birthyear (2010-2011, 2012-2013, 2014-2015), Healthy Eating Index (linear), Parenting Relationship Questionnaire (first three principal components), and age at assessment (linear).  Abbreviations: AKL, Adaptive Skills; BASC-2, Behavioral Assessment System for Children, 2^nd^ Ed.; BSI, Behavioral Symptoms Index; CrI, Credible Interval; EXT, Externalizing Problems; INZ, Internalizing Problems; IQR, Interquartile Range; SRS-2, Social Responsiveness Scale, 2^nd^ Ed. | | | | | | | | |

| Supplemental Table S10. Main effects of each metal at each time point, with imputed covariate data (SRS-2: n = 477; BASC-2: n = 410). Estimate is the difference in the mean predicted outcome (standardized) between the metal fixed at 75% versus 25%, with all other metals fixed at their medians. | | | | | | |
| --- | --- | --- | --- | --- | --- | --- |
| Outcome | Time | Metal | Estimate | SD | 95% Low | 95% High |
| SRS Total | Maternal Prenatal | As | 0.01 | 0.05 | -0.08 | 0.11 |
| SRS Total | Maternal Prenatal | Cu | 0.00 | 0.05 | -0.10 | 0.10 |
| SRS Total | Maternal Prenatal | Mn | -0.07 | 0.06 | -0.18 | 0.04 |
| SRS Total | Maternal Prenatal | Pb | -0.06 | 0.06 | -0.18 | 0.06 |
| SRS Total | Maternal Prenatal | Se | -0.02 | 0.05 | -0.12 | 0.08 |
| SRS Total | Maternal Prenatal | Zn | 0.05 | 0.05 | -0.05 | 0.15 |
| SRS Total | Maternal Postnatal | As | 0.03 | 0.04 | -0.06 | 0.12 |
| SRS Total | Maternal Postnatal | Cu | 0.01 | 0.05 | -0.08 | 0.10 |
| SRS Total | Maternal Postnatal | Mn | 0.05 | 0.05 | -0.04 | 0.15 |
| SRS Total | Maternal Postnatal | Pb | 0.02 | 0.05 | -0.07 | 0.12 |
| SRS Total | Maternal Postnatal | Se | 0.03 | 0.04 | -0.05 | 0.12 |
| SRS Total | Maternal Postnatal | Zn | -0.04 | 0.04 | -0.12 | 0.04 |
| SRS Total | Infant | As | 0.05 | 0.07 | -0.09 | 0.18 |
| SRS Total | Infant | Cu | -0.04 | 0.05 | -0.14 | 0.05 |
| SRS Total | Infant | Mn | 0.05 | 0.06 | -0.06 | 0.16 |
| SRS Total | Infant | Pb | -0.09 | 0.06 | -0.20 | 0.03 |
| SRS Total | Infant | Se | 0.02 | 0.02 | -0.02 | 0.06 |
| SRS Total | Infant | Zn | 0.01 | 0.03 | -0.05 | 0.07 |
| BASC-2 BSI | Maternal Prenatal | As | -0.04 | 0.05 | -0.13 | 0.05 |
| BASC-2 BSI | Maternal Prenatal | Cu | -0.03 | 0.05 | -0.13 | 0.06 |
| BASC-2 BSI | Maternal Prenatal | Mn | 0.04 | 0.05 | -0.07 | 0.15 |
| BASC-2 BSI | Maternal Prenatal | Pb | -0.03 | 0.06 | -0.14 | 0.09 |
| BASC-2 BSI | Maternal Prenatal | Se | 0.04 | 0.05 | -0.05 | 0.14 |
| BASC-2 BSI | Maternal Prenatal | Zn | -0.01 | 0.05 | -0.10 | 0.08 |
| BASC-2 BSI | Maternal Postnatal | As | 0.02 | 0.04 | -0.06 | 0.10 |
| BASC-2 BSI | Maternal Postnatal | Cu | -0.03 | 0.04 | -0.12 | 0.05 |
| BASC-2 BSI | Maternal Postnatal | Mn | -0.01 | 0.05 | -0.10 | 0.08 |
| BASC-2 BSI | Maternal Postnatal | Pb | 0.02 | 0.05 | -0.08 | 0.11 |
| BASC-2 BSI | Maternal Postnatal | Se | 0.04 | 0.04 | -0.05 | 0.12 |
| BASC-2 BSI | Maternal Postnatal | Zn | -0.03 | 0.04 | -0.11 | 0.05 |
| BASC-2 BSI | Infant | As | 0.01 | 0.07 | -0.12 | 0.15 |
| BASC-2 BSI | Infant | Cu | 0.06 | 0.05 | -0.03 | 0.16 |
| BASC-2 BSI | Infant | Mn | 0.12 | 0.06 | 0.00 | 0.23 |
| BASC-2 BSI | Infant | Pb | -0.11 | 0.06 | -0.23 | 0.01 |
| BASC-2 BSI | Infant | Se | 0.01 | 0.02 | -0.03 | 0.06 |
| BASC-2 BSI | Infant | Zn | -0.04 | 0.03 | -0.10 | 0.02 |
| BASC-2 EXT | Maternal Prenatal | As | 0.00 | 0.05 | -0.09 | 0.10 |
| BASC-2 EXT | Maternal Prenatal | Cu | -0.02 | 0.05 | -0.12 | 0.08 |
| BASC-2 EXT | Maternal Prenatal | Mn | 0.01 | 0.06 | -0.10 | 0.11 |
| BASC-2 EXT | Maternal Prenatal | Pb | 0.01 | 0.06 | -0.11 | 0.13 |
| BASC-2 EXT | Maternal Prenatal | Se | 0.04 | 0.05 | -0.06 | 0.13 |
| BASC-2 EXT | Maternal Prenatal | Zn | 0.04 | 0.05 | -0.06 | 0.13 |
| BASC-2 EXT | Maternal Postnatal | As | 0.00 | 0.04 | -0.08 | 0.08 |
| BASC-2 EXT | Maternal Postnatal | Cu | -0.05 | 0.05 | -0.14 | 0.04 |
| BASC-2 EXT | Maternal Postnatal | Mn | -0.03 | 0.05 | -0.12 | 0.06 |
| BASC-2 EXT | Maternal Postnatal | Pb | 0.00 | 0.05 | -0.09 | 0.10 |
| BASC-2 EXT | Maternal Postnatal | Se | 0.03 | 0.05 | -0.06 | 0.11 |
| BASC-2 EXT | Maternal Postnatal | Zn | -0.01 | 0.04 | -0.09 | 0.07 |
| BASC-2 EXT | Infant | As | -0.01 | 0.07 | -0.15 | 0.12 |
| BASC-2 EXT | Infant | Cu | 0.08 | 0.05 | -0.02 | 0.17 |
| BASC-2 EXT | Infant | Mn | 0.09 | 0.06 | -0.02 | 0.21 |
| BASC-2 EXT | Infant | Pb | -0.11 | 0.06 | -0.23 | 0.01 |
| BASC-2 EXT | Infant | Se | 0.00 | 0.02 | -0.05 | 0.04 |
| BASC-2 EXT | Infant | Zn | -0.03 | 0.03 | -0.10 | 0.03 |
| BASC-2 INZ | Maternal Prenatal | As | -0.04 | 0.05 | -0.14 | 0.06 |
| BASC-2 INZ | Maternal Prenatal | Cu | -0.05 | 0.06 | -0.15 | 0.06 |
| BASC-2 INZ | Maternal Prenatal | Mn | 0.04 | 0.06 | -0.07 | 0.16 |
| BASC-2 INZ | Maternal Prenatal | Pb | -0.04 | 0.07 | -0.17 | 0.09 |
| BASC-2 INZ | Maternal Prenatal | Se | 0.00 | 0.05 | -0.10 | 0.10 |
| BASC-2 INZ | Maternal Prenatal | Zn | 0.01 | 0.05 | -0.09 | 0.11 |
| BASC-2 INZ | Maternal Postnatal | As | 0.04 | 0.04 | -0.05 | 0.12 |
| BASC-2 INZ | Maternal Postnatal | Cu | -0.03 | 0.05 | -0.13 | 0.06 |
| BASC-2 INZ | Maternal Postnatal | Mn | -0.03 | 0.05 | -0.13 | 0.06 |
| BASC-2 INZ | Maternal Postnatal | Pb | 0.04 | 0.05 | -0.06 | 0.15 |
| BASC-2 INZ | Maternal Postnatal | Se | 0.03 | 0.05 | -0.07 | 0.12 |
| BASC-2 INZ | Maternal Postnatal | Zn | -0.03 | 0.04 | -0.11 | 0.06 |
| BASC-2 INZ | Infant | As | 0.05 | 0.07 | -0.09 | 0.20 |
| BASC-2 INZ | Infant | Cu | 0.05 | 0.05 | -0.06 | 0.15 |
| BASC-2 INZ | Infant | Mn | -0.05 | 0.06 | -0.17 | 0.07 |
| BASC-2 INZ | Infant | Pb | -0.01 | 0.07 | -0.14 | 0.12 |
| BASC-2 INZ | Infant | Se | 0.02 | 0.02 | -0.03 | 0.06 |
| BASC-2 INZ | Infant | Zn | 0.01 | 0.03 | -0.05 | 0.08 |
| BASC-2 AKL | Maternal Prenatal | As | 0.03 | 0.05 | -0.07 | 0.12 |
| BASC-2 AKL | Maternal Prenatal | Cu | 0.04 | 0.05 | -0.06 | 0.14 |
| BASC-2 AKL | Maternal Prenatal | Mn | 0.00 | 0.06 | -0.11 | 0.11 |
| BASC-2 AKL | Maternal Prenatal | Pb | -0.04 | 0.06 | -0.16 | 0.09 |
| BASC-2 AKL | Maternal Prenatal | Se | -0.06 | 0.05 | -0.16 | 0.04 |
| BASC-2 AKL | Maternal Prenatal | Zn | 0.09 | 0.05 | -0.01 | 0.18 |
| BASC-2 AKL | Maternal Postnatal | As | -0.01 | 0.04 | -0.10 | 0.07 |
| BASC-2 AKL | Maternal Postnatal | Cu | 0.03 | 0.05 | -0.06 | 0.12 |
| BASC-2 AKL | Maternal Postnatal | Mn | -0.07 | 0.05 | -0.16 | 0.02 |
| BASC-2 AKL | Maternal Postnatal | Pb | 0.03 | 0.05 | -0.06 | 0.13 |
| BASC-2 AKL | Maternal Postnatal | Se | -0.05 | 0.05 | -0.14 | 0.04 |
| BASC-2 AKL | Maternal Postnatal | Zn | 0.04 | 0.04 | -0.04 | 0.12 |
| BASC-2 AKL | Infant | As | 0.07 | 0.07 | -0.07 | 0.21 |
| BASC-2 AKL | Infant | Cu | -0.06 | 0.05 | -0.16 | 0.04 |
| BASC-2 AKL | Infant | Mn | -0.11 | 0.06 | -0.23 | 0.01 |
| BASC-2 AKL | Infant | Pb | 0.11 | 0.06 | -0.02 | 0.23 |
| BASC-2 AKL | Infant | Se | 0.00 | 0.02 | -0.04 | 0.04 |
| BASC-2 AKL | Infant | Zn | 0.03 | 0.03 | -0.03 | 0.10 |
| Models adjusted for maternal age (quadratic), maternal BMI (quadratic), highest level of parental education (high school or less, any college, any graduate), sex (male, female), parity (0, ≥1), smoking status (no second- or first-hand, ever second-hand only, ever first-hand), age at last breastfeeding (<365 days, ≥365 days), maternal marital status (married, other), birth year (2010-2011, 2012-2013, 2014-2015), Healthy Eating Index (linear), Parenting Relationship Questionnaire (first three principal components), and age at assessment (linear).  Abbreviations: AKL, Adaptive Skills; BASC-2, Behavioral Assessment System for Children, 2^nd^ Ed.; BSI, Behavioral Symptoms Index; CrI, Credible Interval; EXT, Externalizing Problems; INZ, Internalizing Problems; IQR, Interquartile Range; SRS-2, Social Responsiveness Scale, 2^nd^ Ed. | | | | | | |

| Supplemental Table S11. Linear models containing metal biomarkers and their interactions identified as statistically significant at α = 0.05 in MFVB-LKMR analyses. | | | |
| --- | --- | --- | --- |
| Outcome | Exposure | Beta^a^ | 95% CI |
| SRS-2 Total Score | As, Infant | 0.48 | (-1.87, 2.83) |
|  | Se, Infant | -0.77 | (-2.55, 1.00) |
|  | (As, Infant)*(Se, Infant) | -0.44 | (-1.60, 0.72) |
| BASC-2 Behavioral Symptoms Index | As, Maternal Postnatal | 4.25 | (1.84, 6.66) |
|  | Mn, Infant | 4.35 | (0.04, 8.65) |
| BASC-2 Externalizing Problems | Mn, Infant | 0.90 | (-0.84, 2.64) |
| BASC-2 Internalizing Problems | Pb, Maternal Prenatal | -39.53 | (-91.57, 12.51) |
|  | Zn, Maternal Prenatal | 3.40 | (-1.03, 7.82) |
|  | As, Maternal Postnatal | 1.49 | (-0.87, 3.85) |
|  | (Pb, Maternal Prenatal)*(Zn, Maternal Prenatal) | 2.29 | (-0.75, 5.33) |
| BASC-2 Adaptive Skills | As, Infant | -3.75 | (-6.90, -0.61) |
|  | Mn, Infant | -0.33 | (-3.60, 2.93) |
|  | Se, Infant | 2.70 | (-1.73, 7.12) |
|  | (As, Infant)*(Se, Infant) | -0.58 | (-2.56, 1.41) |
| Models adjusted for maternal age (quadratic), maternal BMI (quadratic), highest level of parental education (high school or less, any college, any graduate), sex (male, female), parity (0, ≥1), smoking status (no second- or first-hand, ever second-hand only, ever first-hand), age at last breastfeeding (<365 days, ≥365 days), maternal marital status (married, other), birth year (2010-2011, 2012-2013, 2014-2015), Healthy Eating Index (linear), Parenting Relationship Questionnaire (first three principal components), age at assessment (linear), and the first three principal components of all metal biomarkers not included as main effects or interactions in the model.  ^a^Change in outcome per interquartile range increase in exposure.  Abbreviations: BASC-2, Behavioral Assessment System for Children, 2^nd^ Ed.; MFVB-LKMR, Mean Field Variational Bayes for Lagged Kernel Machine Regression; SRS-2, Social Responsiveness Scale, 2^nd^ Ed. | | | |
