# Supplementary material for: Periconceptional and prenatal exposure to metal mixtures in relation to behavioral development at 3 years of age
Source: Environ Epidemiol. 2020 Jul 6;4(4):e0106. doi: 10.1097/EE9.0000000000000106 (PMC7595192; doi:10.1097/EE9.0000000000000106)
Supplement: Supplementary file 2 [file ee9-4-e0106-s002.pdf]

SRS−2 Total Score

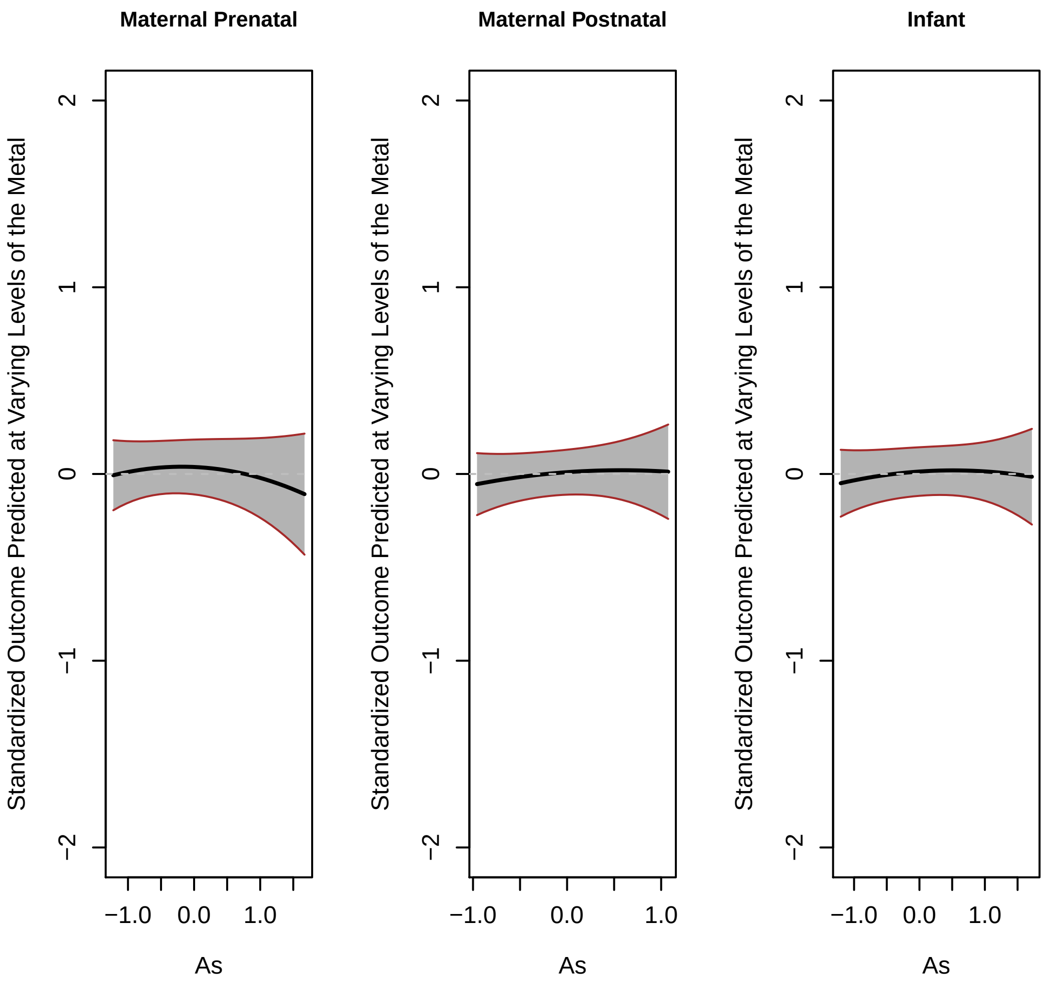

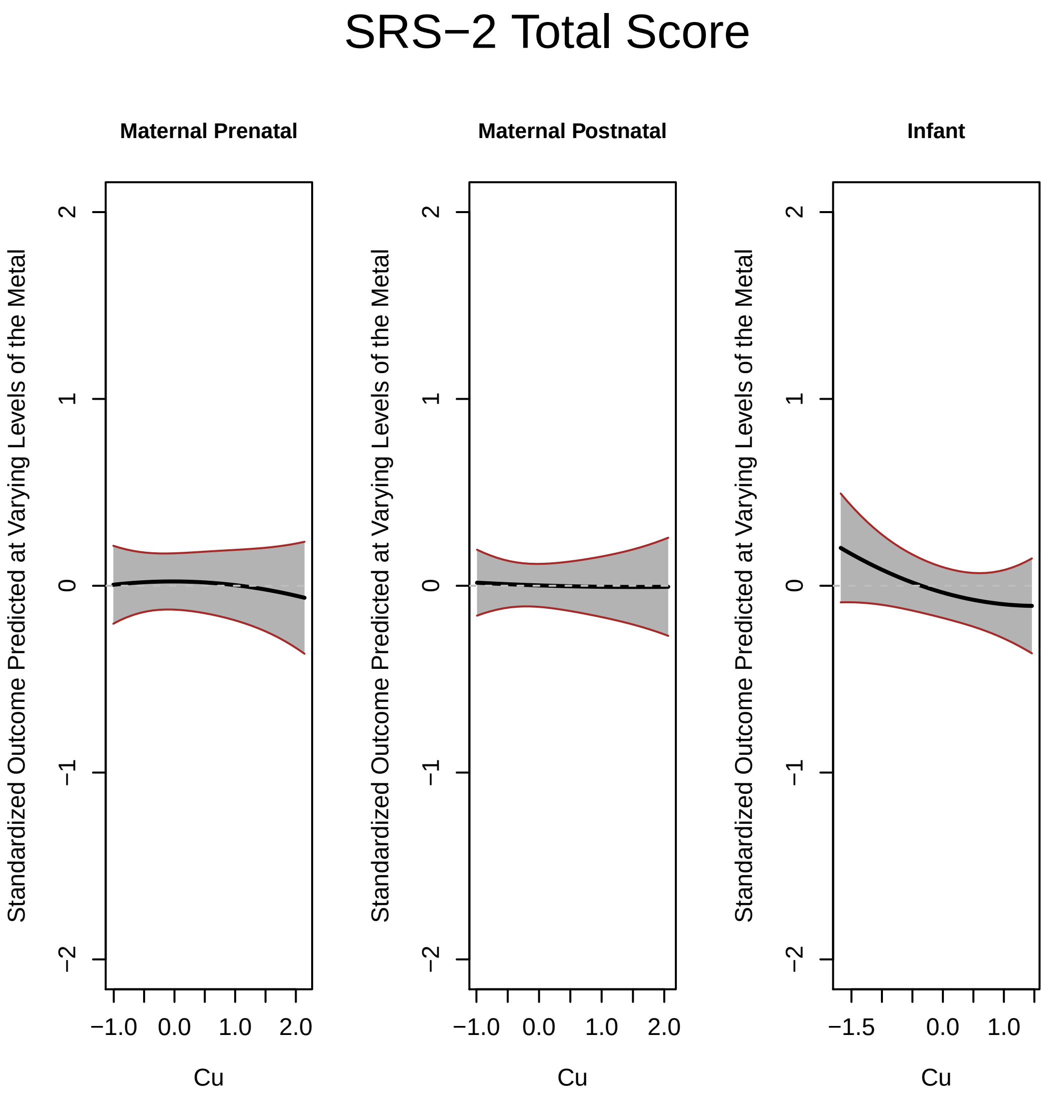

SRS−2 Total Score
Maternal Prenatal
Maternal Postnatal
Infant
Standardized Outcome Predicted at Varying Levels of the Metal
2
1
0
−1
−2
−1.0
0.0
1.0
2.0
−1.5
Cu

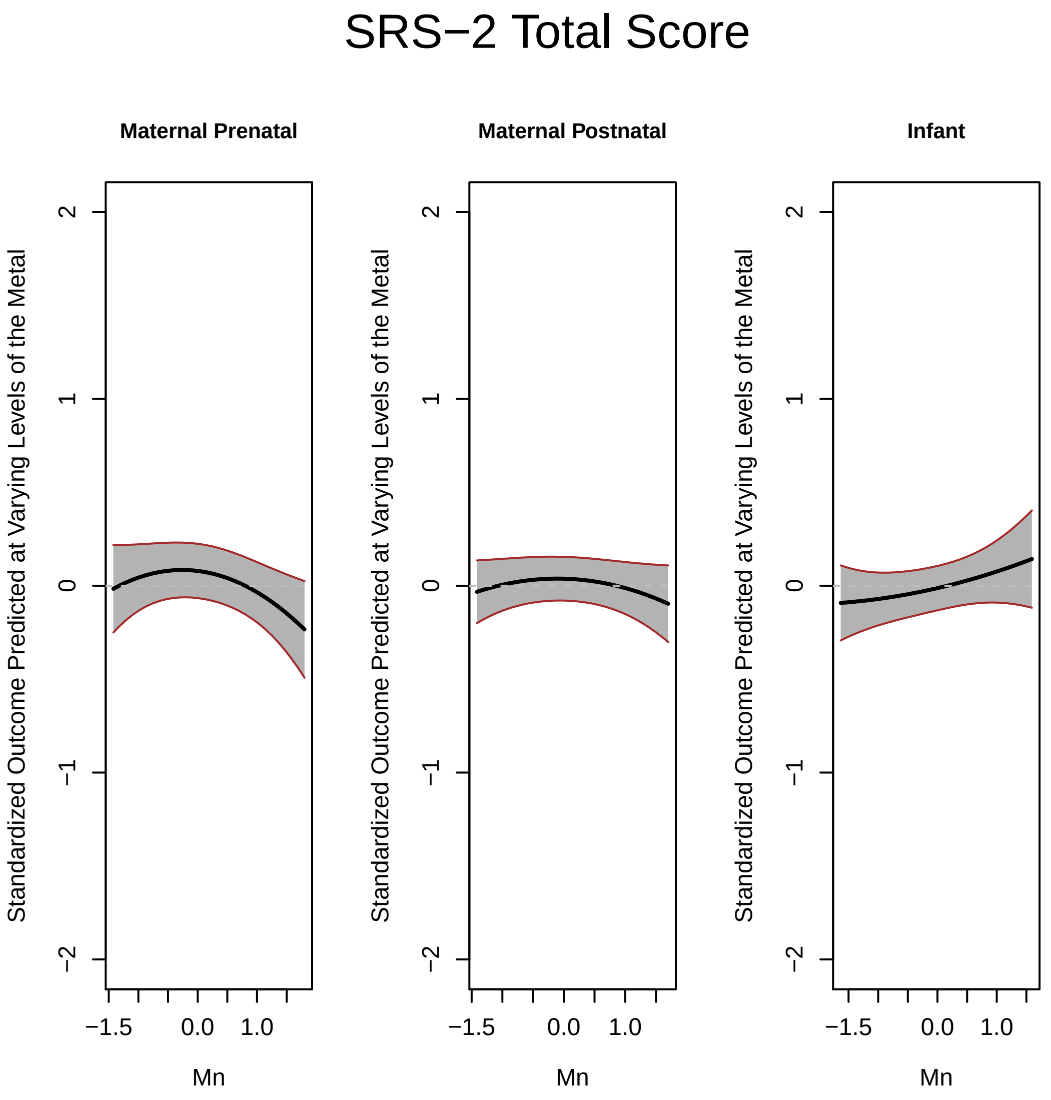
SRS−2 Total Score
Maternal Prenatal
Maternal Postnatal
Infant
Standardized Outcome Predicted at Varying Levels of the Metal
−2
−1
0
1
2
−1.5
0.0
1.0
Mn

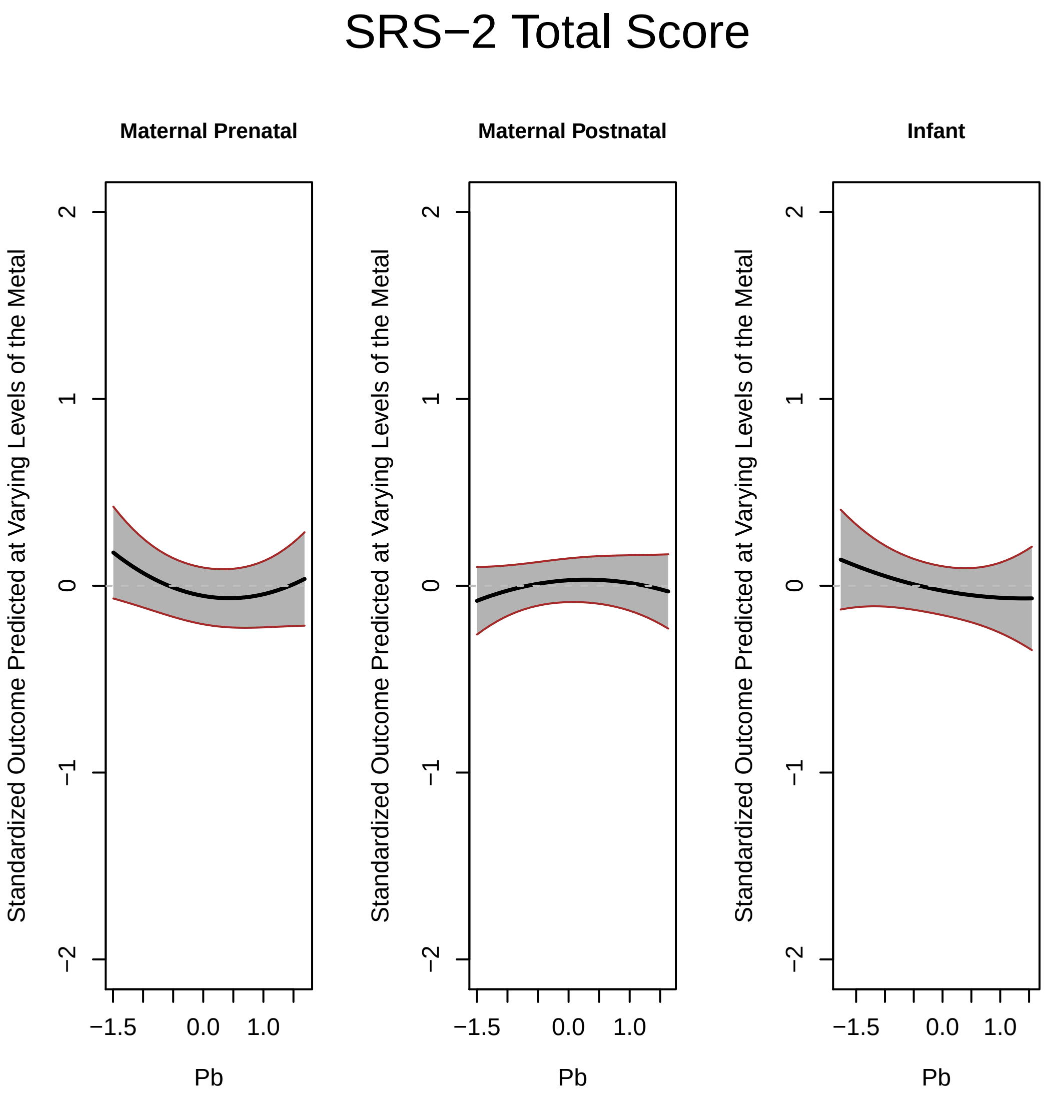
SRS−2 Total Score
Maternal Prenatal
Maternal Postnatal
Infant
Standardized Outcome Predicted at Varying Levels of the Metal
2
1
0
−1
−2
−1.5
0.0
1.0
Pb

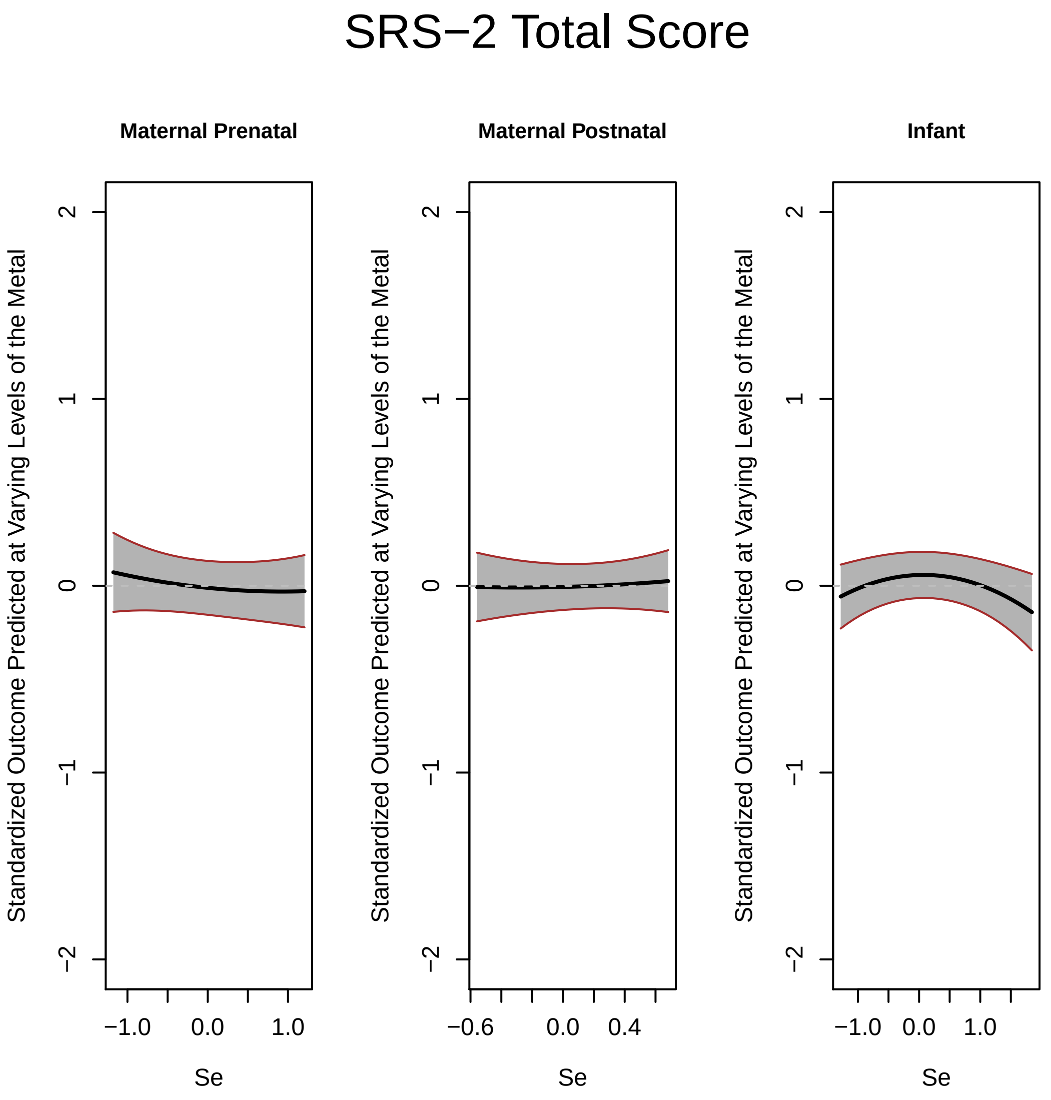
SRS−2 Total Score
Maternal Prenatal
Maternal Postnatal
Infant
Standardized Outcome Predicted at Varying Levels of the Metal
2
1
0
−1
−2
−1.0
0.0
1.0
−0.6
0.4
Se

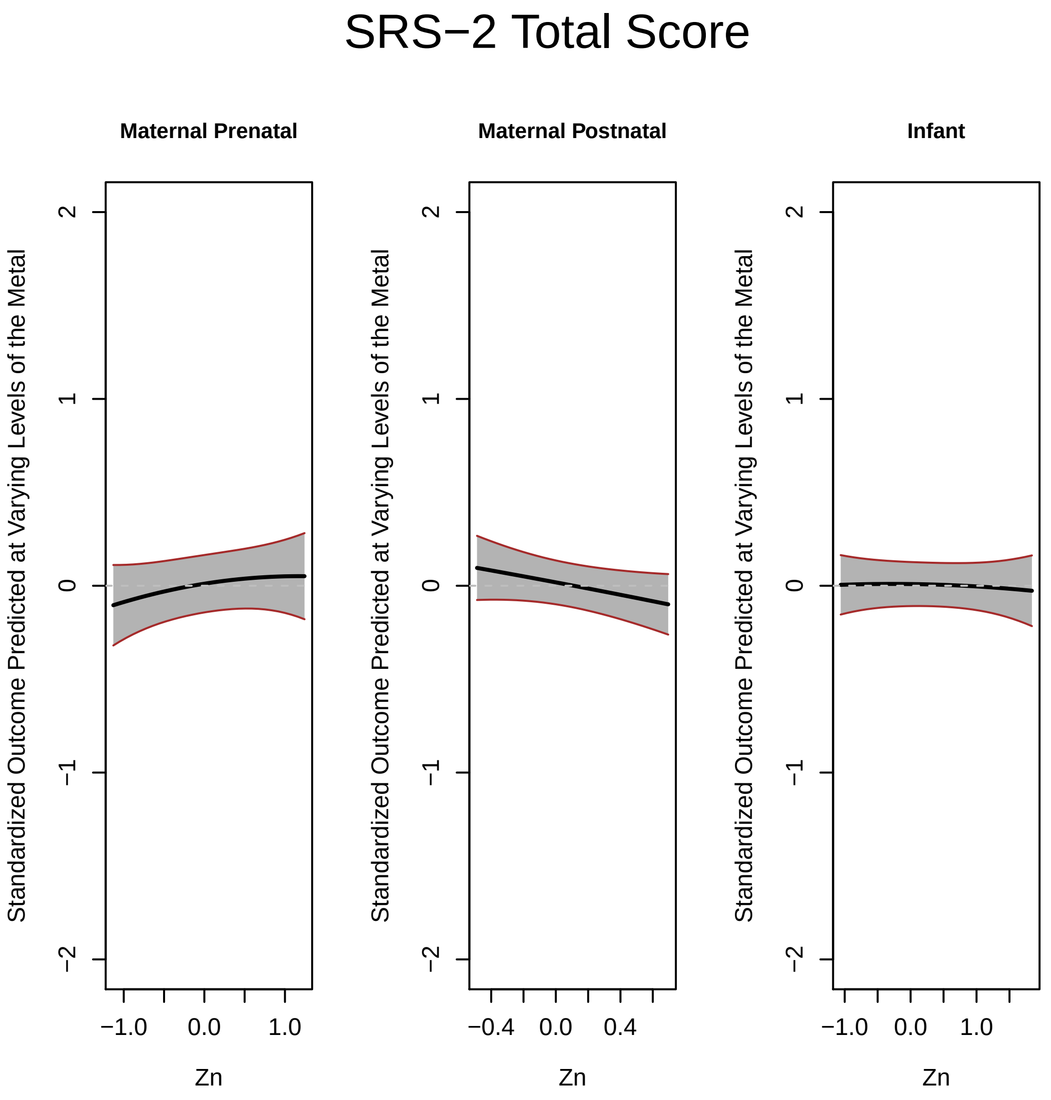

SRS−2 Total Score
Maternal Prenatal
Maternal Postnatal
Infant
Standardized Outcome Predicted at Varying Levels of the Metal
2
1
0
−1
−2
−1.0
0.0
1.0
−0.4
0.0
0.4
Zn

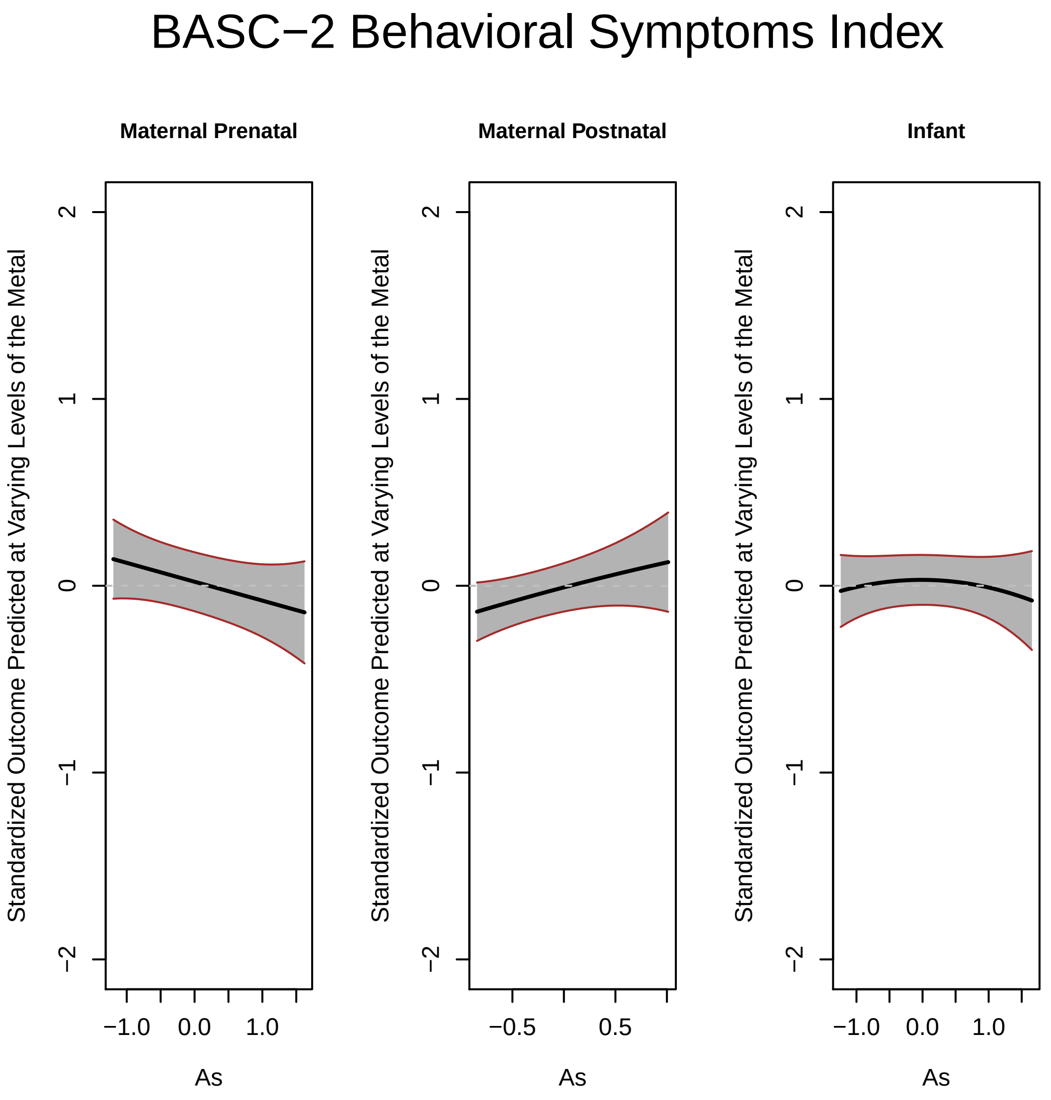

BASC−2 Behavioral Symptoms Index
Maternal Prenatal
Maternal Postnatal
Infant
Standardized Outcome Predicted at Varying Levels of the Metal
2
1
0
−1
−2
−1.0
0.0
1.0
−0.5
0.5
As

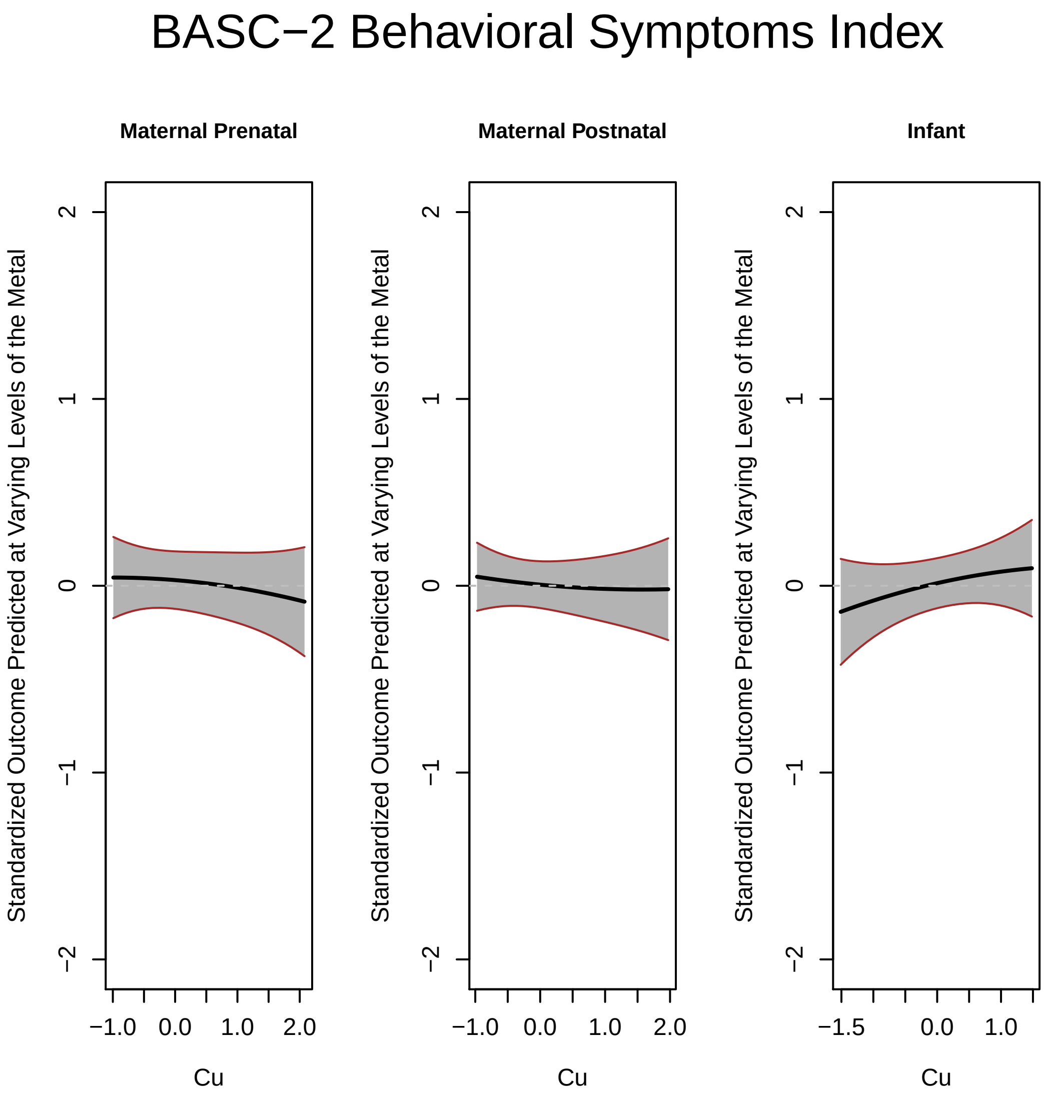

BASC−2 Behavioral Symptoms Index
Maternal Prenatal
Maternal Postnatal
Infant
Standardized Outcome Predicted at Varying Levels of the Metal
2
1
0
−1
−2
−1.0
0.0
1.0
2.0
−1.5
Cu

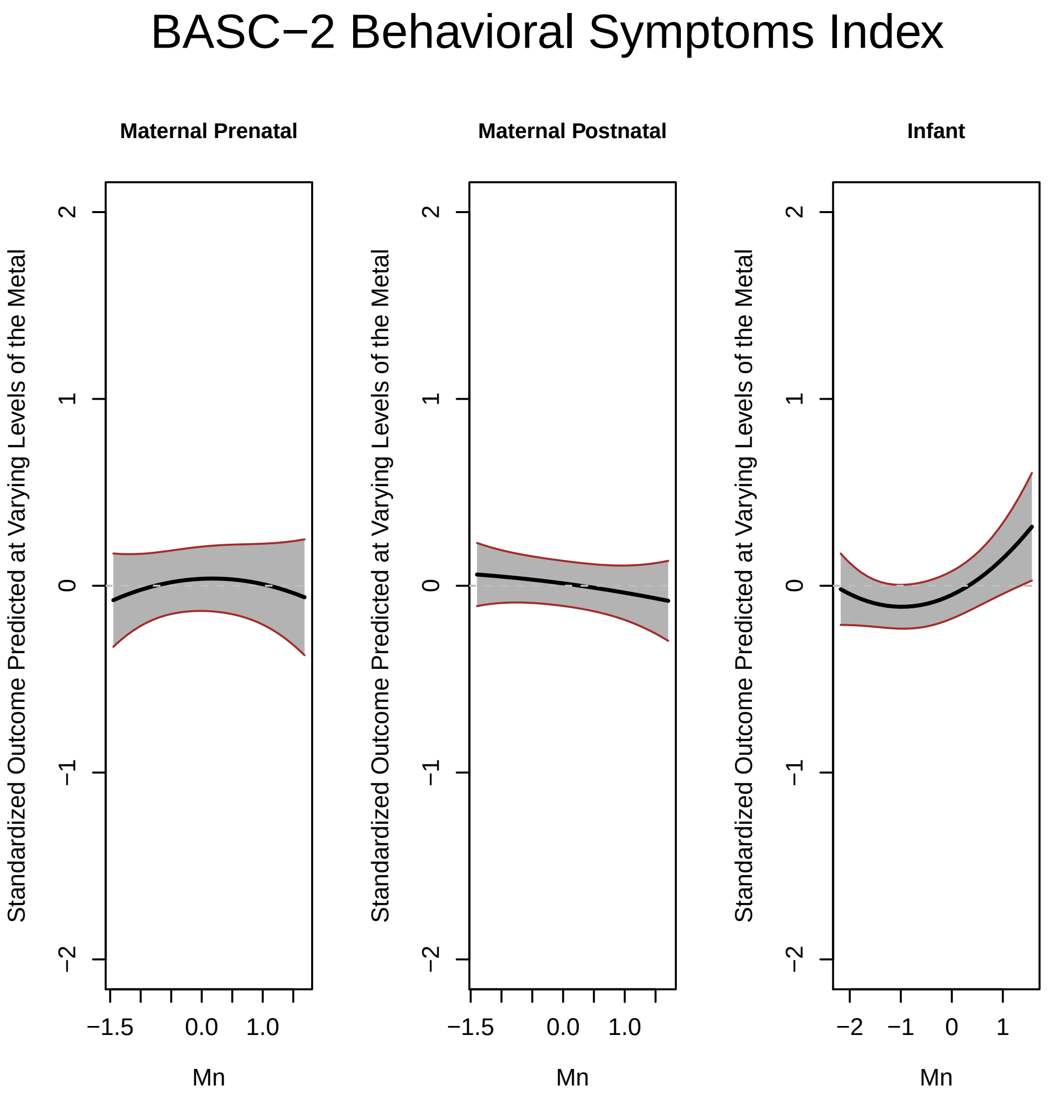

BASC−2 Behavioral Symptoms Index
Maternal Prenatal
Maternal Postnatal
Infant
Standardized Outcome Predicted at Varying Levels of the Metal
−2
−1
0
1
2
−1.5
0.0
1.0
Mn

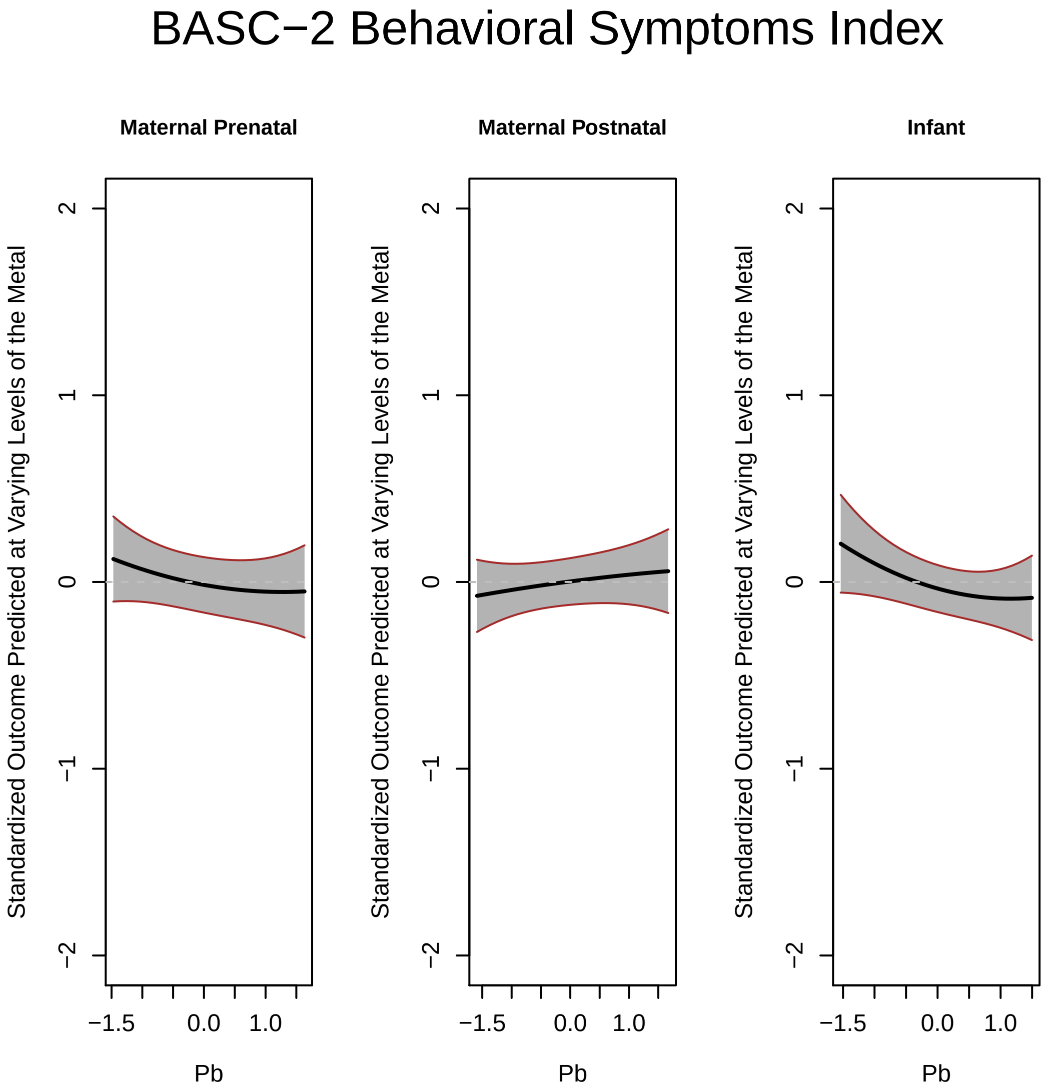
BASC−2 Behavioral Symptoms Index
Maternal Prenatal
Maternal Postnatal
Infant
Standardized Outcome Predicted at Varying Levels of the Metal
2
1
0
−1
−2
−1.5
0.0
1.0
Pb

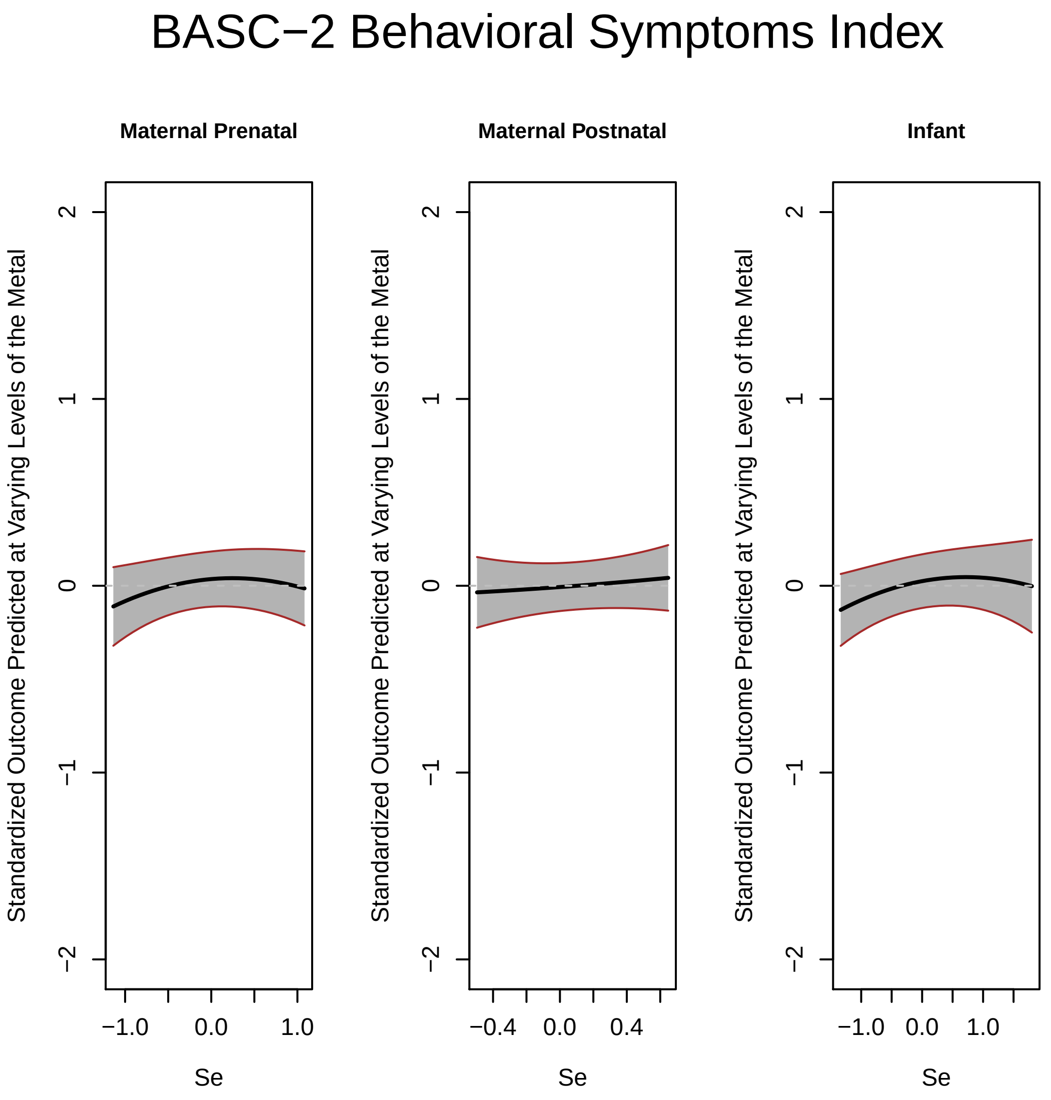
BASC−2 Behavioral Symptoms Index
Maternal Prenatal
Maternal Postnatal
Infant
Standardized Outcome Predicted at Varying Levels of the Metal
2
1
0
−1
−2
−1.0
0.0
1.0
−0.4
0.0
0.4
Se

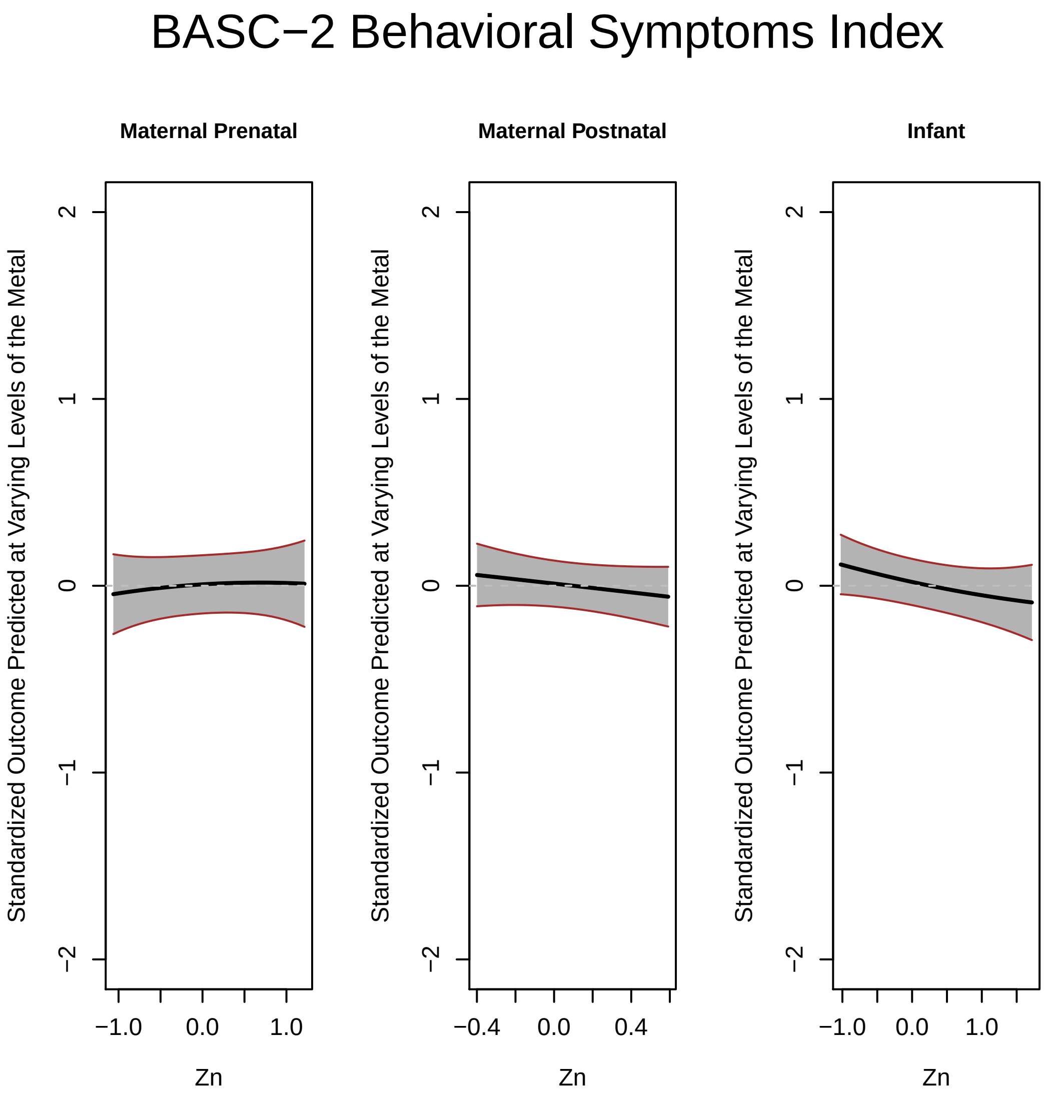
BASC−2 Behavioral Symptoms Index
Maternal Prenatal
Maternal Postnatal
Infant
Standardized Outcome Predicted at Varying Levels of the Metal
2
1
0
−1
−2
−1.0
0.0
1.0
−0.4
0.0
0.4
Zn

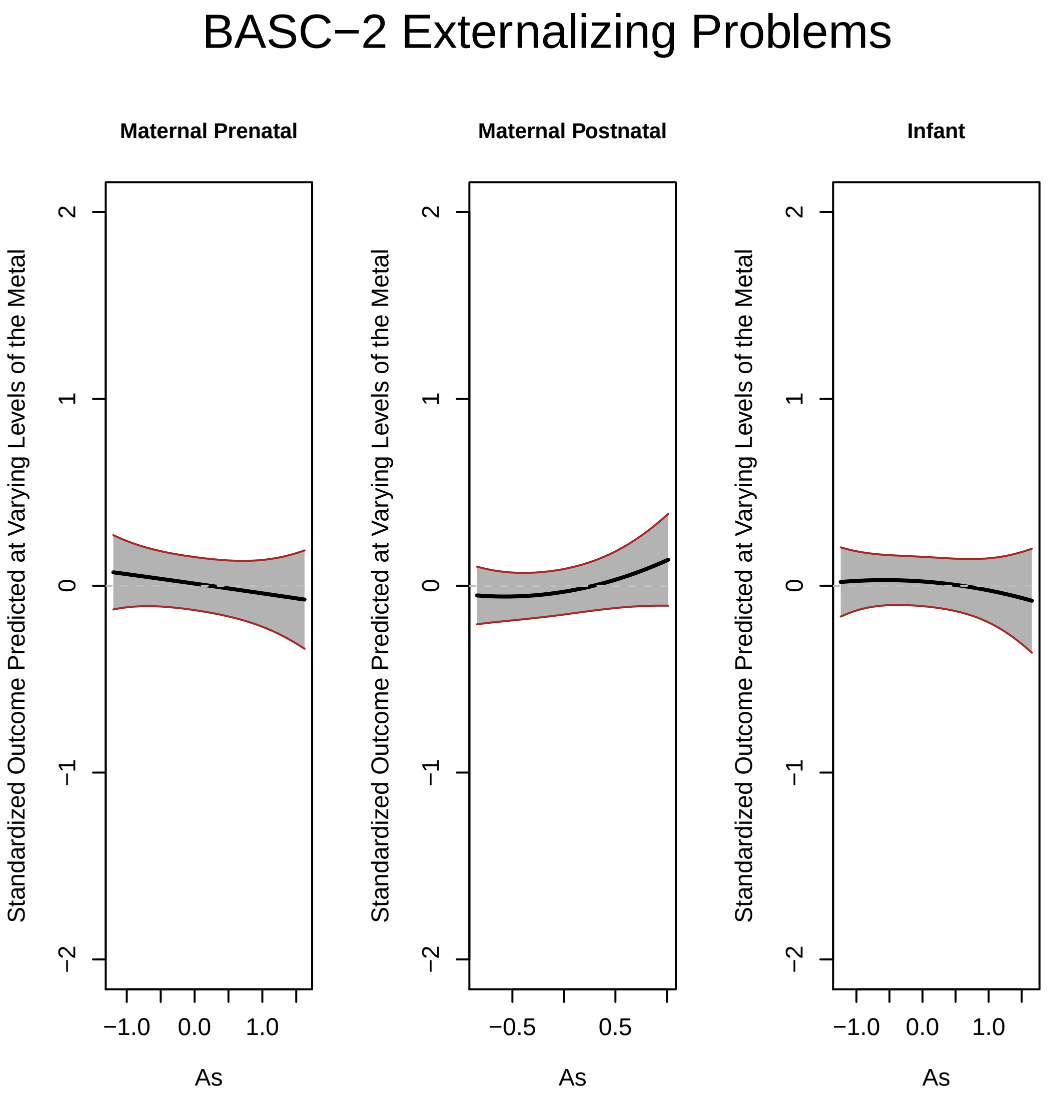

BASC−2 Externalizing Problems
Maternal Prenatal
Maternal Postnatal
Infant
Standardized Outcome Predicted at Varying Levels of the Metal
2
1
0
−1
−2
−1.0
0.0
1.0
−0.5
0.5
As

BASC−2 Externalizing Problems

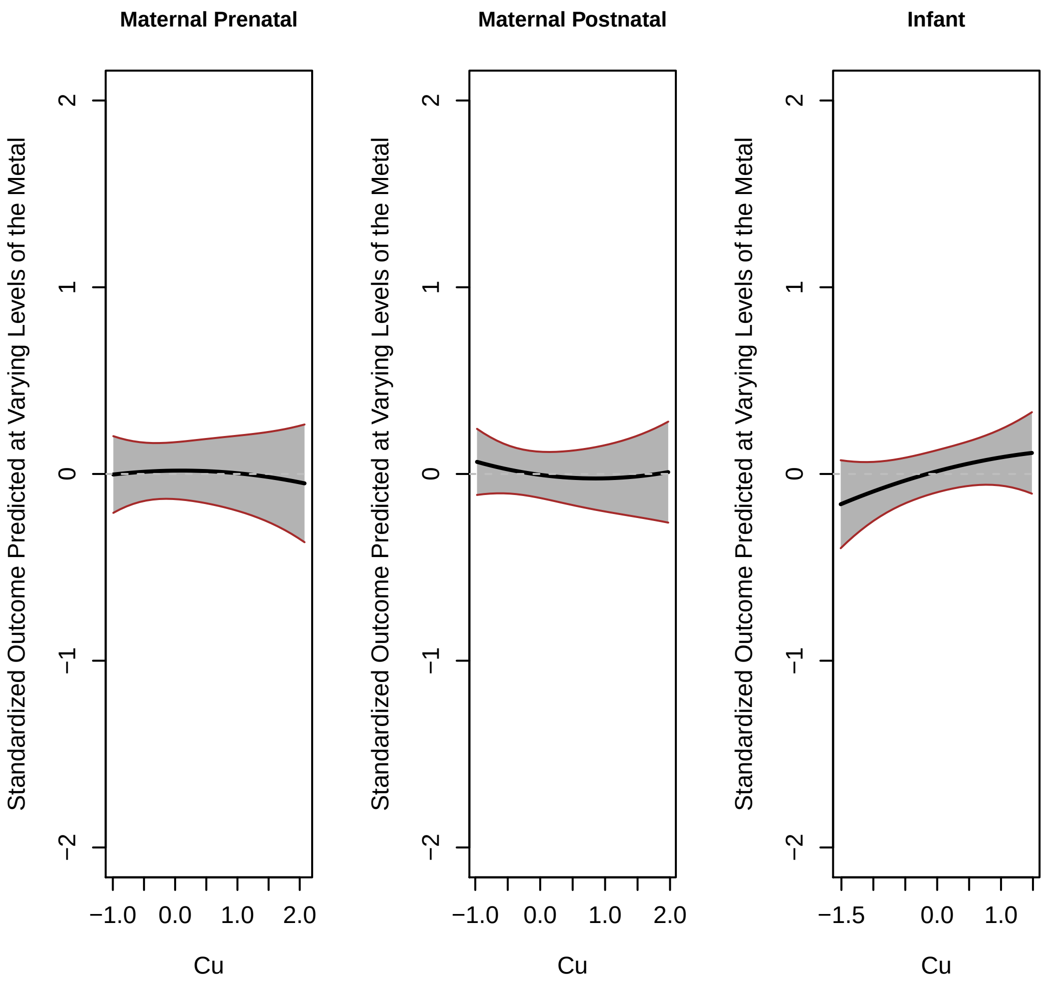

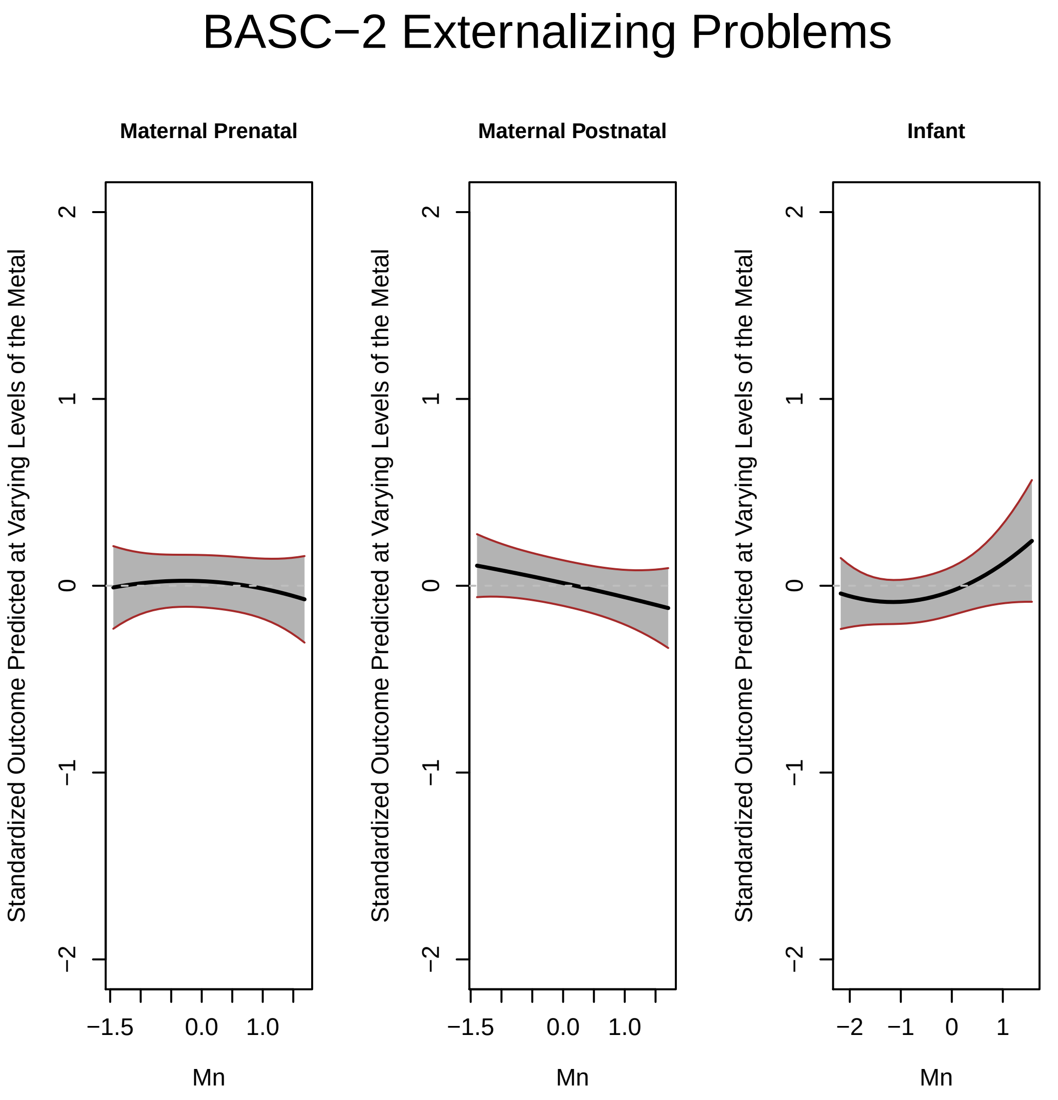
BASC−2 Externalizing Problems
Maternal Prenatal
Maternal Postnatal
Infant
Standardized Outcome Predicted at Varying Levels of the Metal
2
1
0
−1
−2
−1.5
0.0
1.0
−2
−1
0
1
Mn

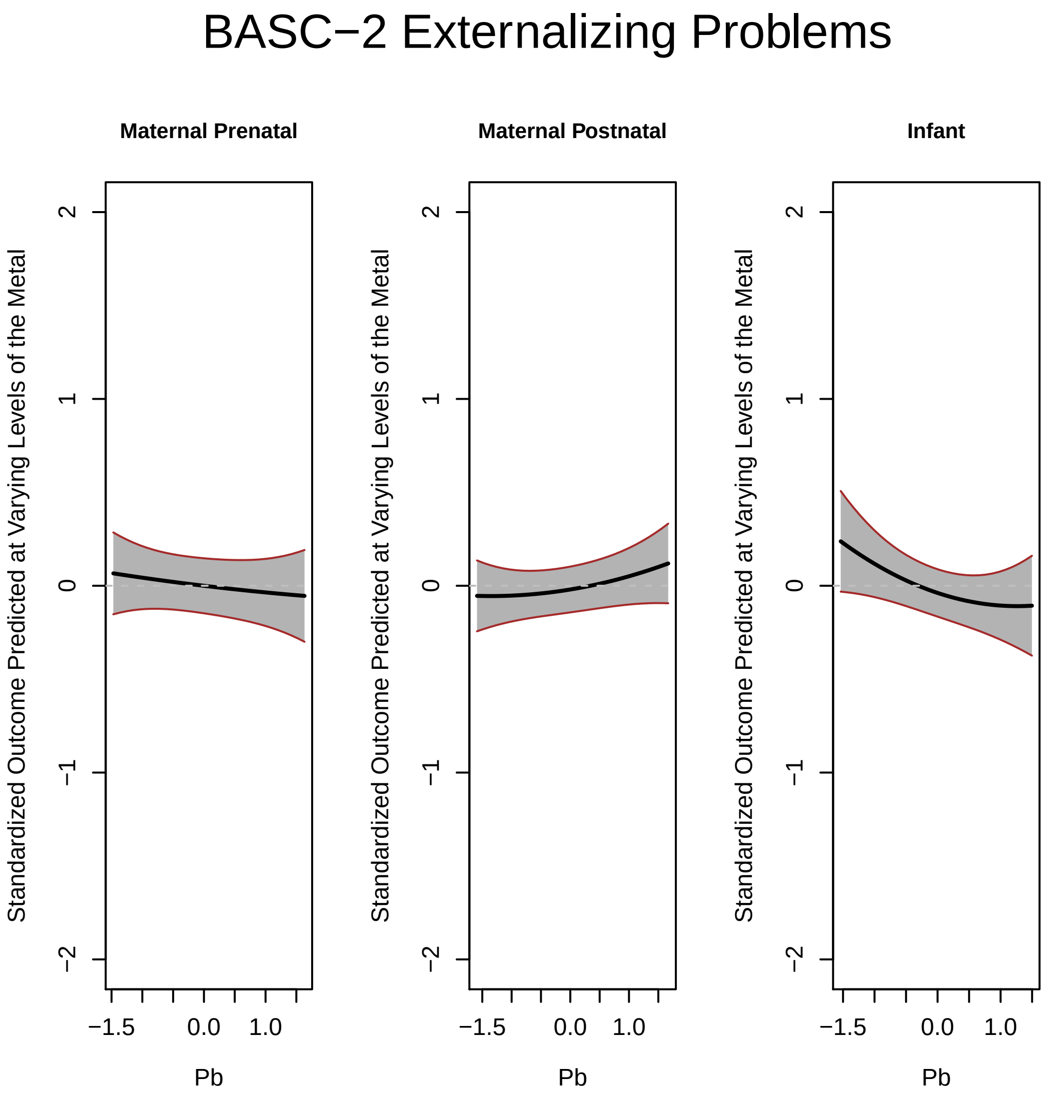
BASC−2 Externalizing Problems
Maternal Prenatal
Maternal Postnatal
Infant
Standardized Outcome Predicted at Varying Levels of the Metal
2
1
0
−1
−2
−1.5
0.0
1.0
Pb

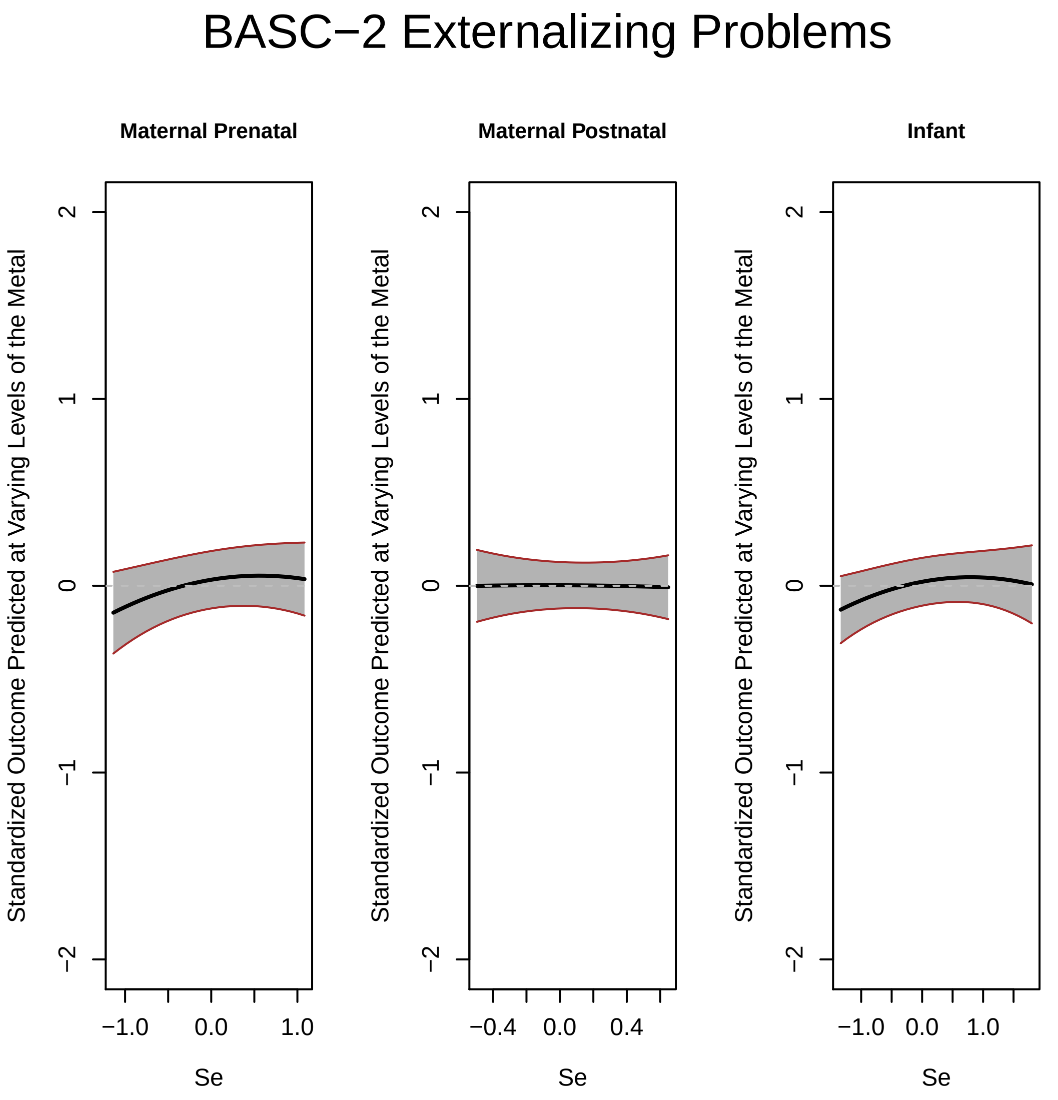

BASC−2 Externalizing Problems
Maternal Prenatal
Maternal Postnatal
Infant
Standardized Outcome Predicted at Varying Levels of the Metal
2
1
0
−1
−2
−1.0
0.0
1.0
−0.4
0.0
0.4
Se

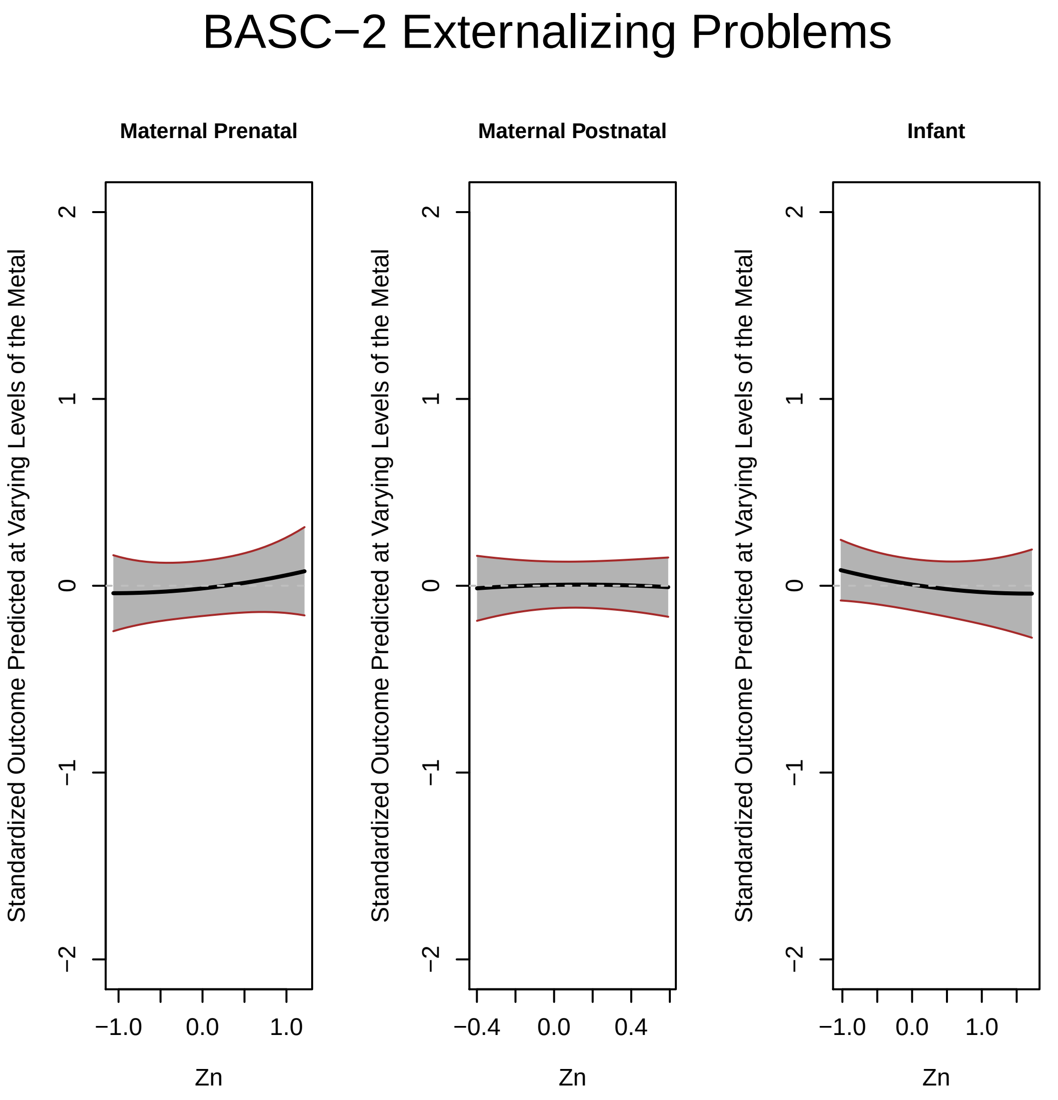
BASC−2 Externalizing Problems
Maternal Prenatal
Maternal Postnatal
Infant
Standardized Outcome Predicted at Varying Levels of the Metal
Zn
−2
−1
0
1
2
−1.0
0.0
1.0
−0.4
0.0
0.4

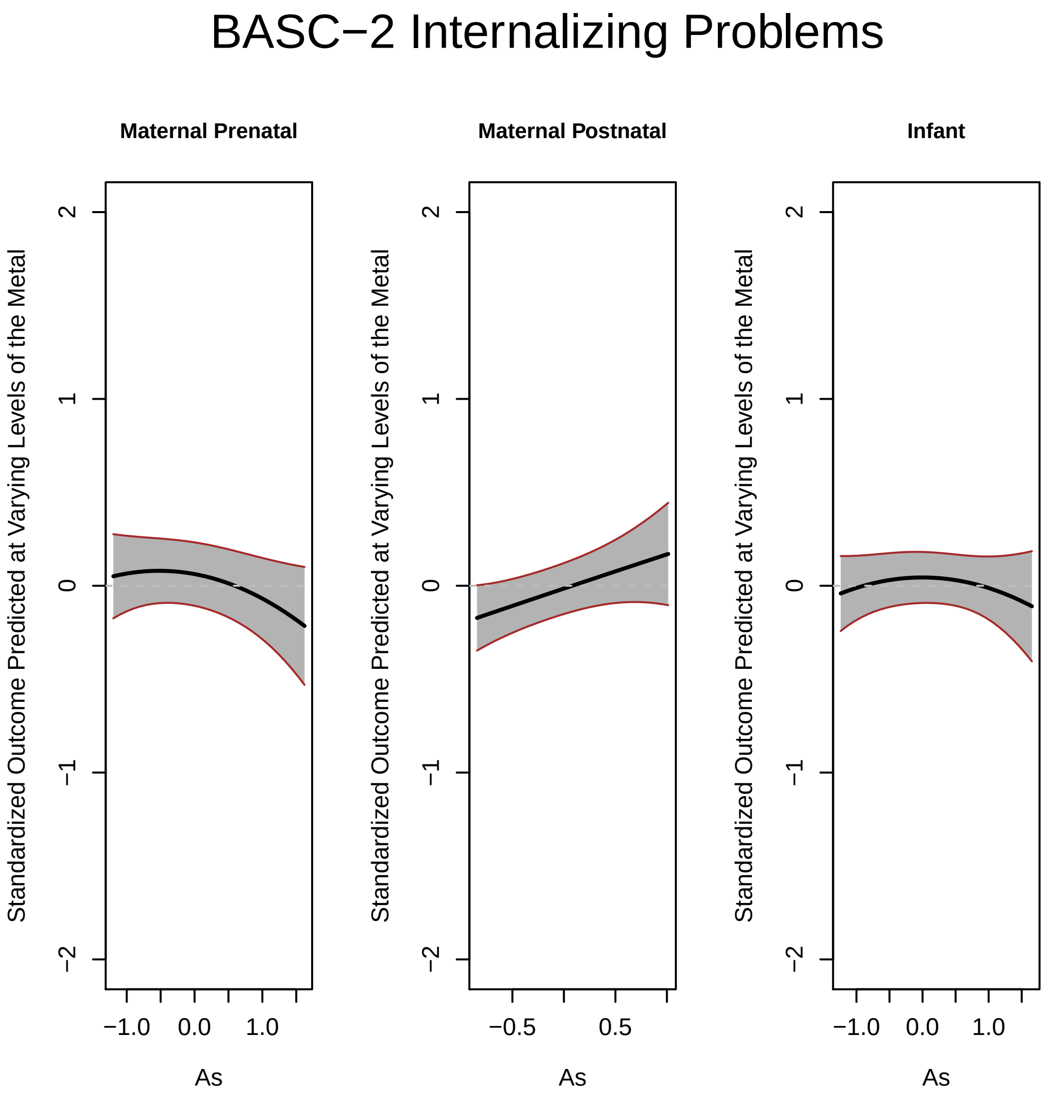

BASC−2 Internalizing Problems
Maternal Prenatal
Maternal Postnatal
Infant
Standardized Outcome Predicted at Varying Levels of the Metal
2
1
0
−1
−2
−1.0
0.0
1.0
−0.5
0.5
As

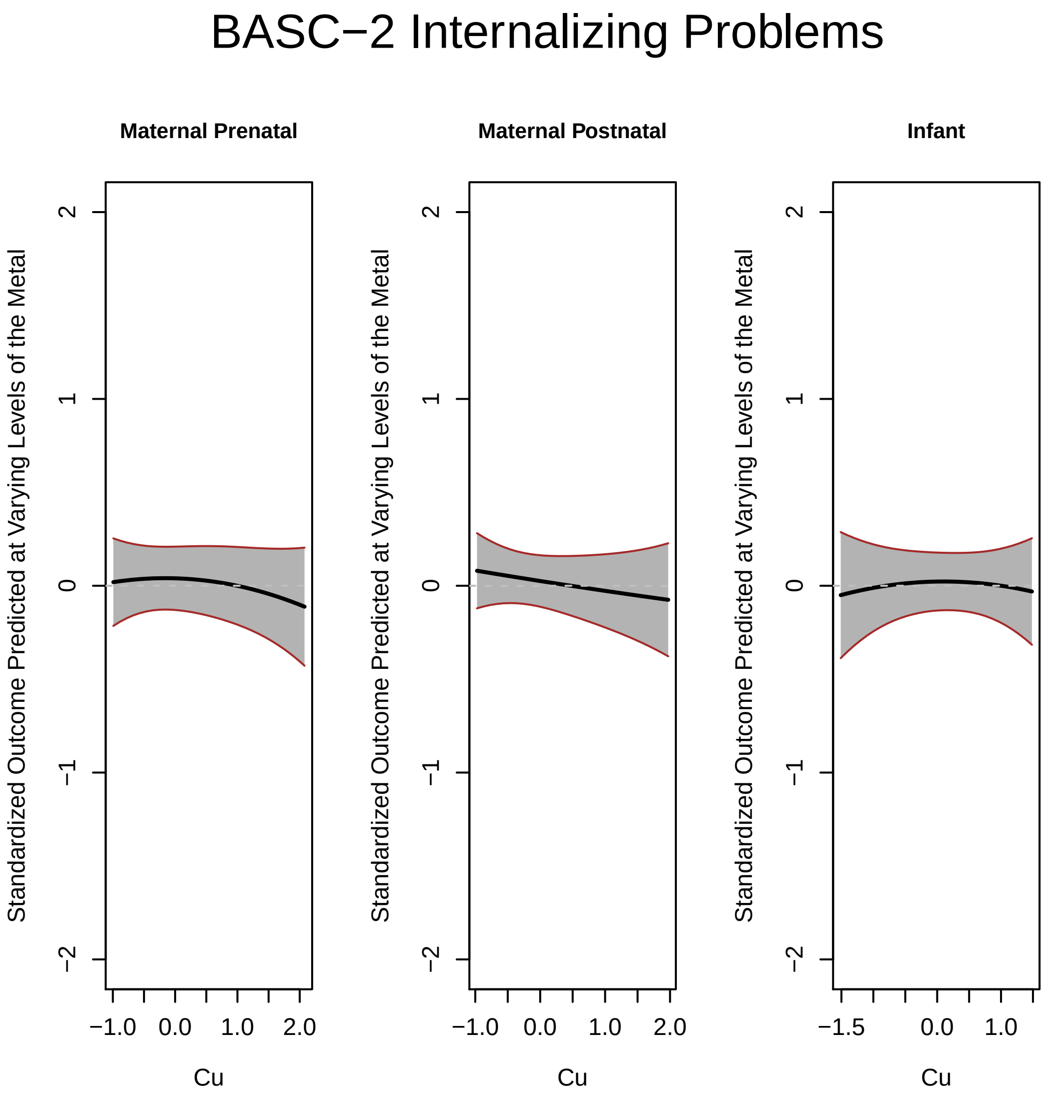

BASC−2 Internalizing Problems
Maternal Prenatal
Maternal Postnatal
Infant
Standardized Outcome Predicted at Varying Levels of the Metal
2
1
0
−1
−2
−1.0
0.0
1.0
2.0
−1.5
Cu

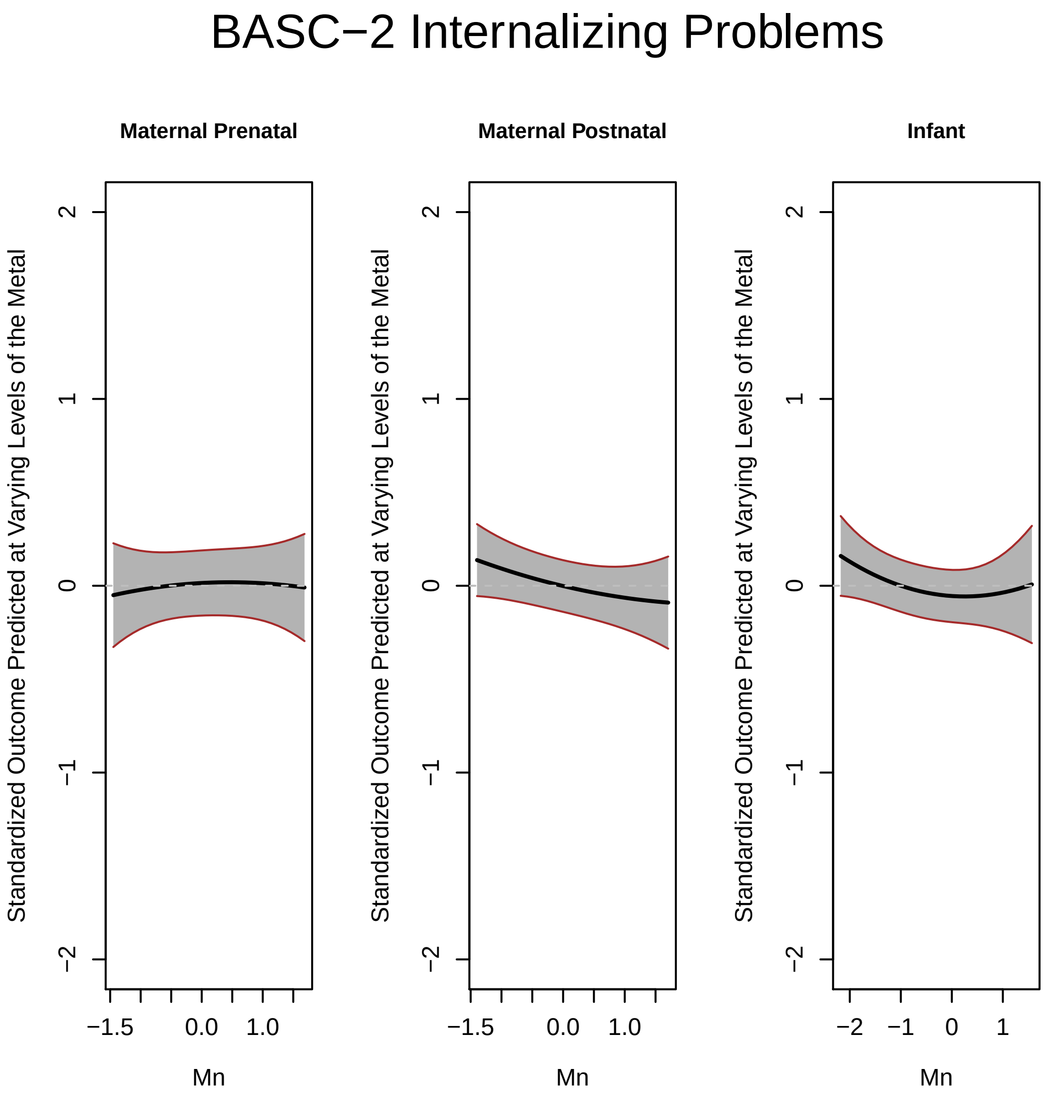

BASC−2 Internalizing Problems
Maternal Prenatal
Maternal Postnatal
Infant
Standardized Outcome Predicted at Varying Levels of the Metal
2
1
0
−1
−2
−1.5
0.0
1.0
Mn
−2
−1
0
1

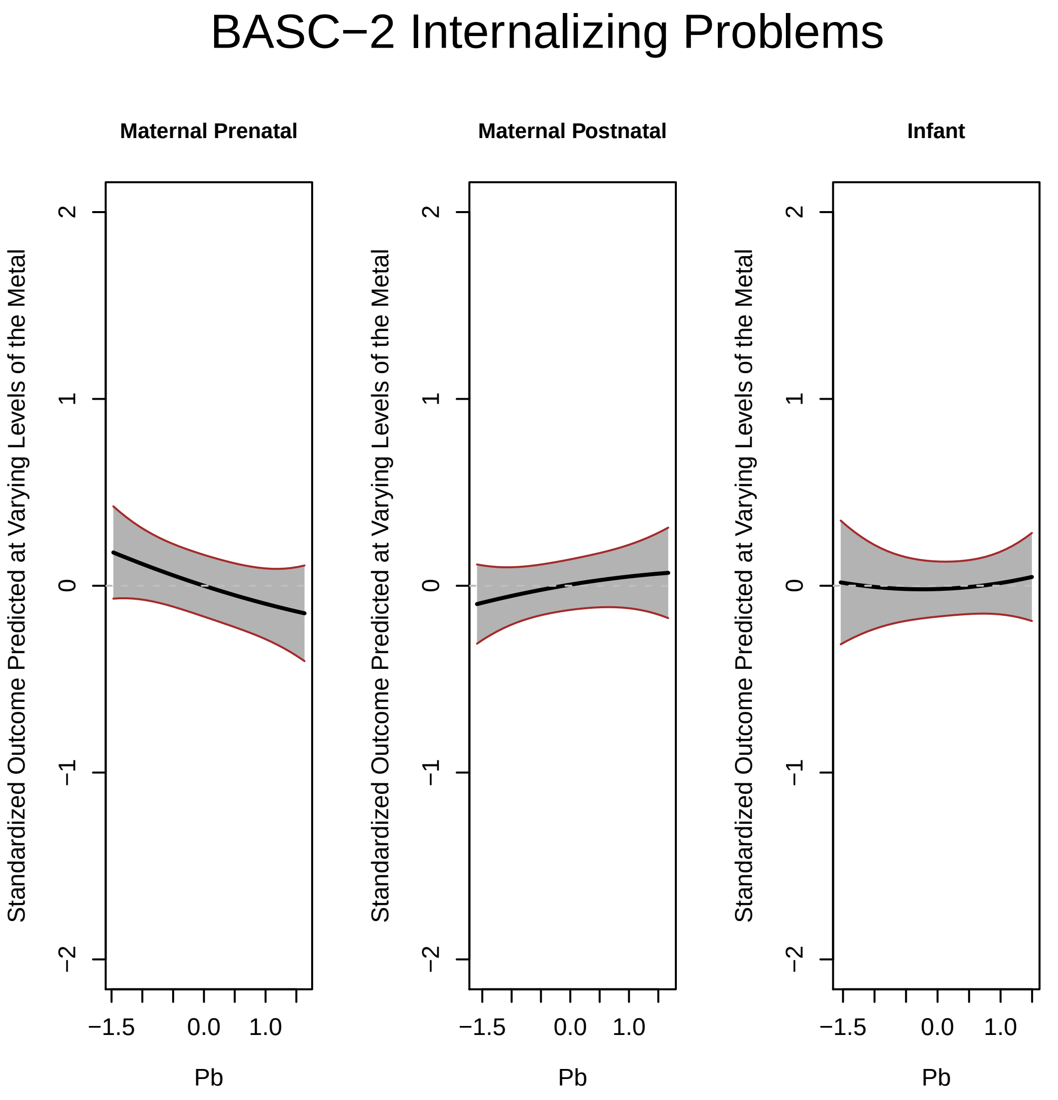
BASC−2 Internalizing Problems
Maternal Prenatal
Maternal Postnatal
Infant
Standardized Outcome Predicted at Varying Levels of the Metal
2
1
0
−1
−2
−1.5
0.0
1.0
Pb

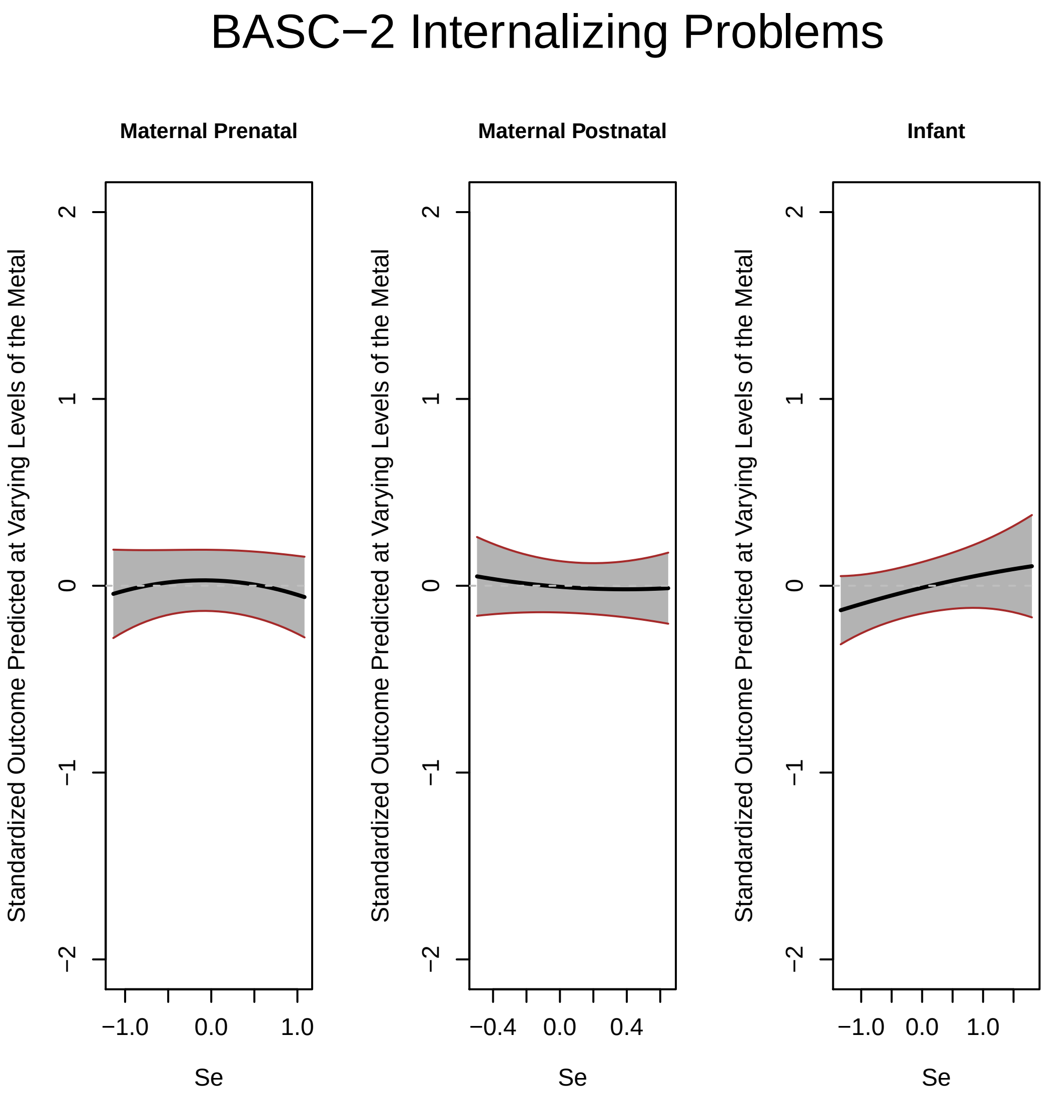
BASC−2 Internalizing Problems
Maternal Prenatal
Maternal Postnatal
Infant
Standardized Outcome Predicted at Varying Levels of the Metal
2
1
0
−1
−2
−1.0
0.0
1.0
−0.4
0.0
0.4
Se

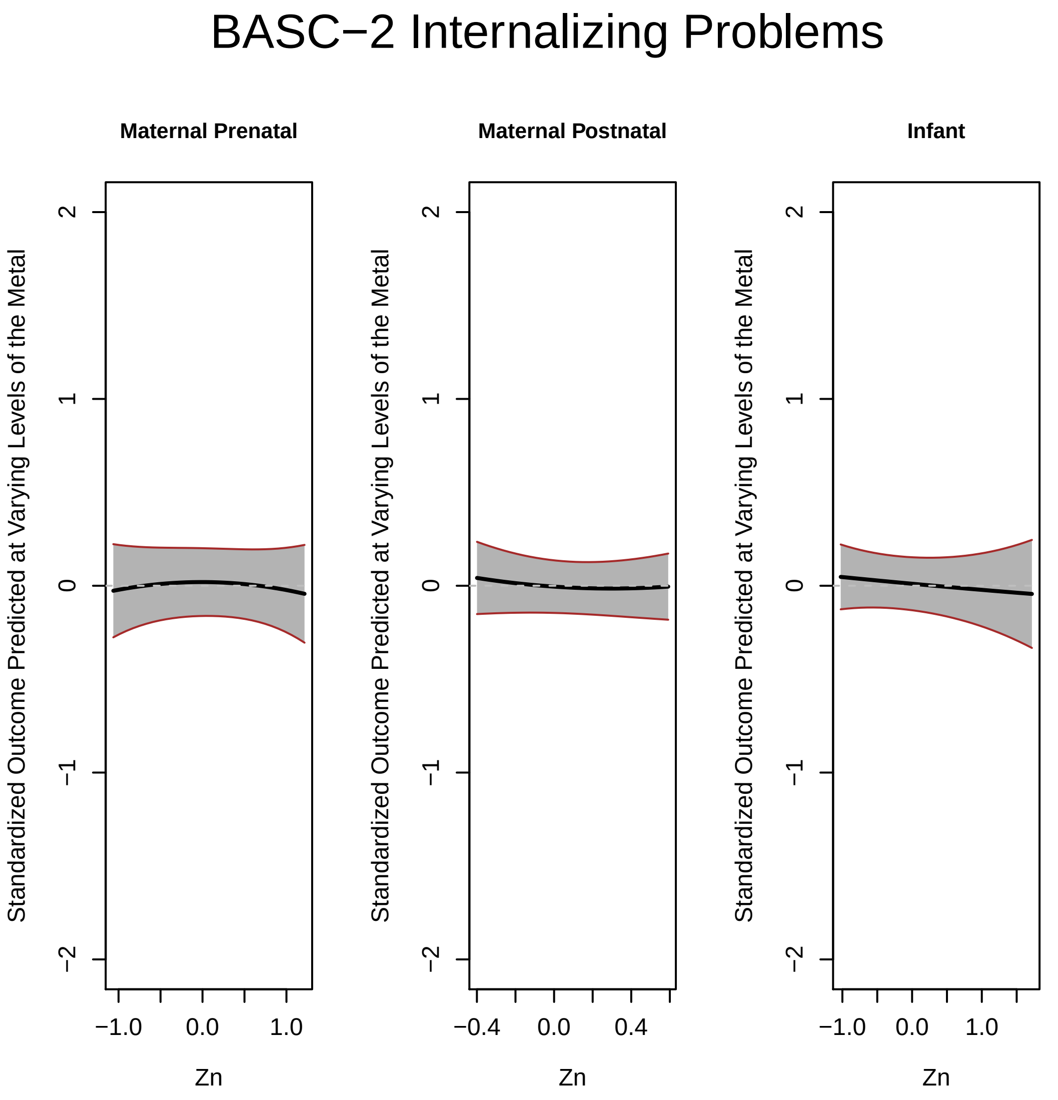
BASC−2 Internalizing Problems
Maternal Prenatal
Maternal Postnatal
Infant
Standardized Outcome Predicted at Varying Levels of the Metal
2
1
0
−1
−2
−1.0
0.0
1.0
−0.4
0.0
0.4
Zn

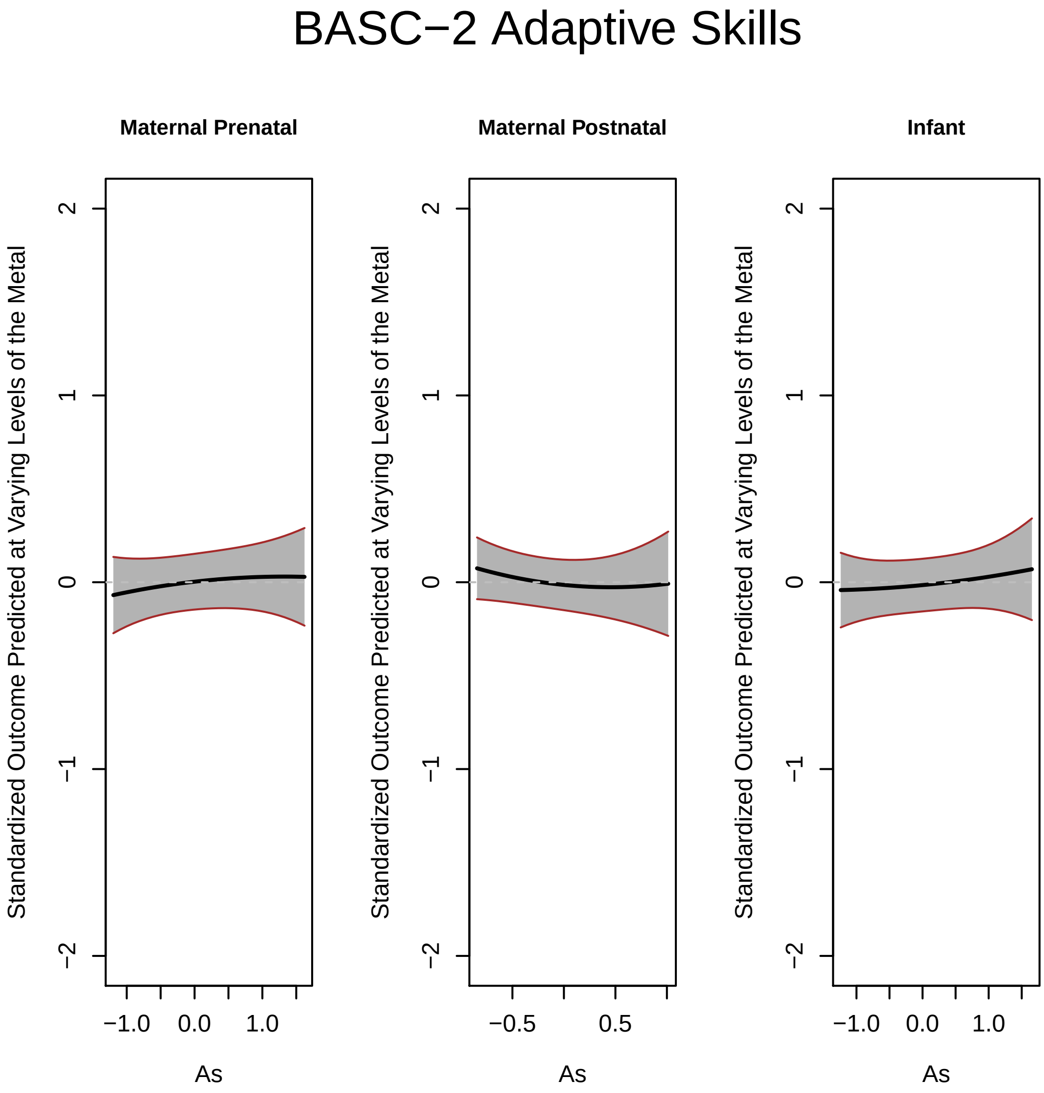
BASC−2 Adaptive Skills
Maternal Prenatal
Maternal Postnatal
Infant
Standardized Outcome Predicted at Varying Levels of the Metal
2
1
0
−1
−2
−1.0
0.0
1.0
−0.5
0.5
As

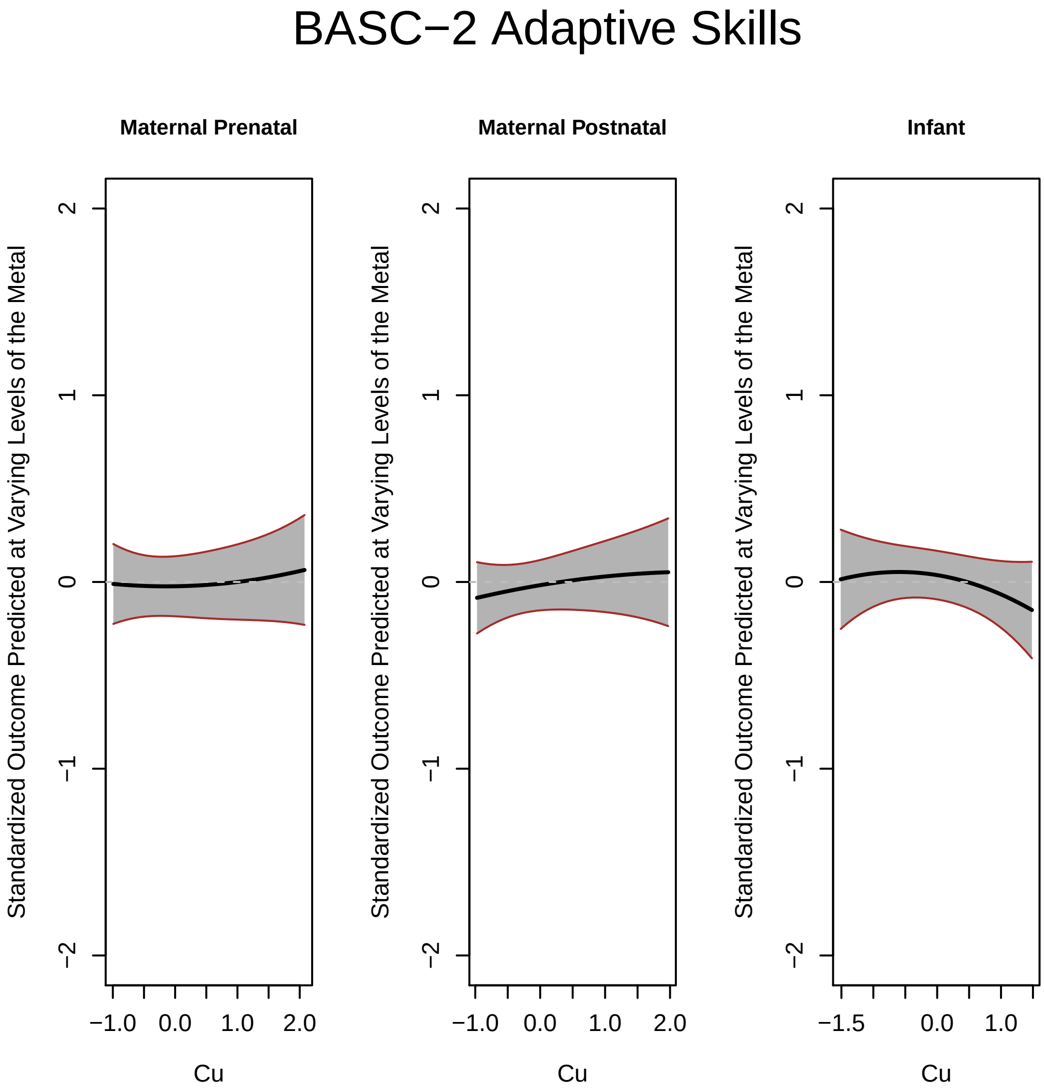

BASC−2 Adaptive Skills
Maternal Prenatal
Maternal Postnatal
Infant
Standardized Outcome Predicted at Varying Levels of the Metal
2
1
0
−1
−2
−1.0
0.0
1.0
2.0
−1.5
Cu

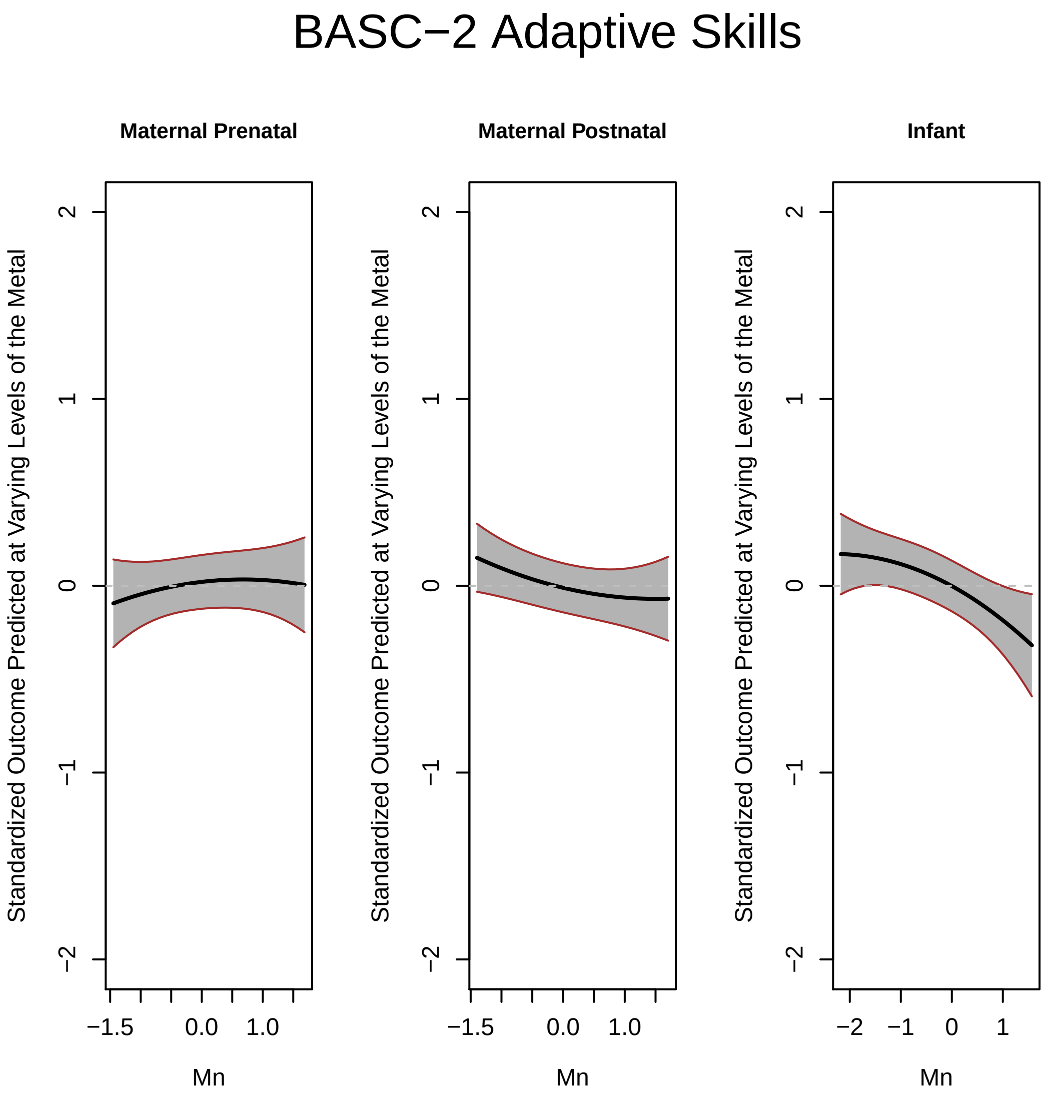
BASC−2 Adaptive Skills
Maternal Prenatal
Maternal Postnatal
Infant
Standardized Outcome Predicted at Varying Levels of the Metal
2
1
0
−1
−2
−1.5
0.0
1.0
−2
−1
0
1
Mn

BASC−2 Adaptive Skills

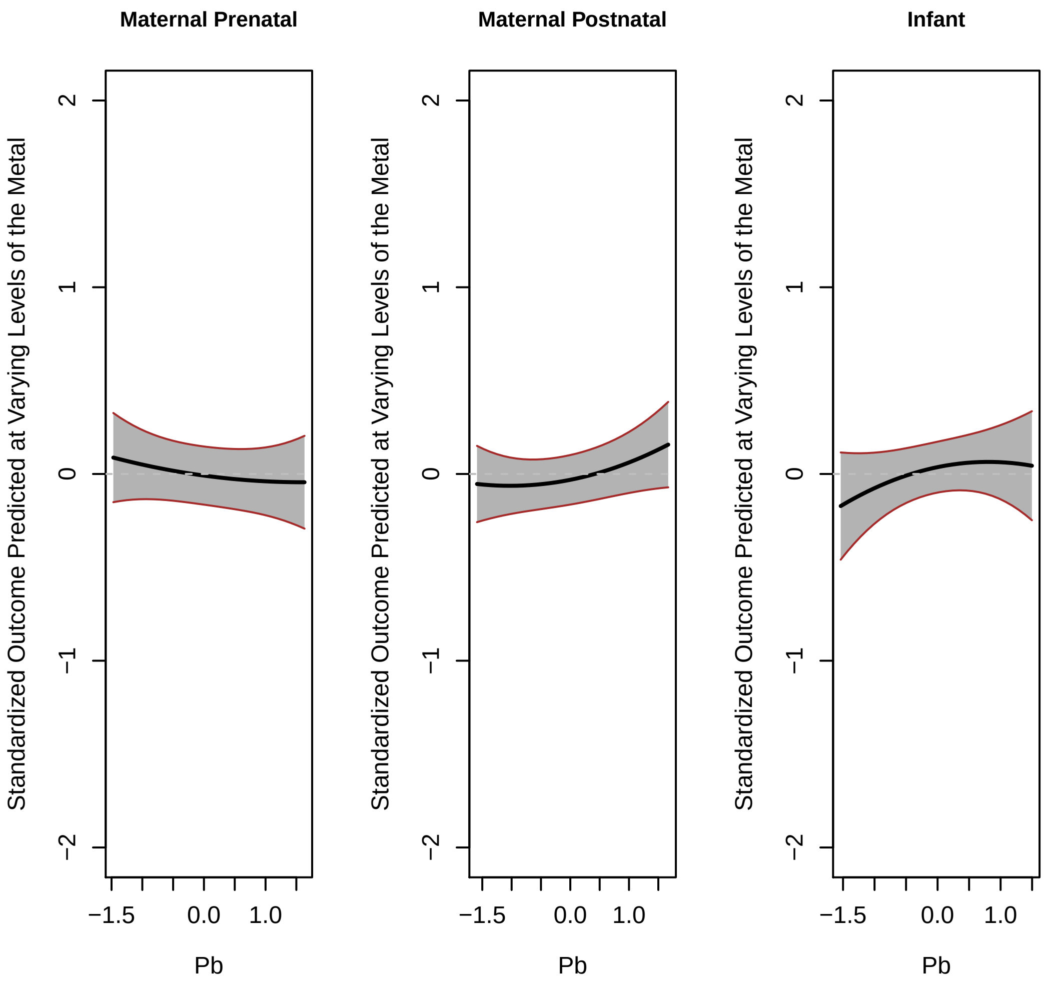

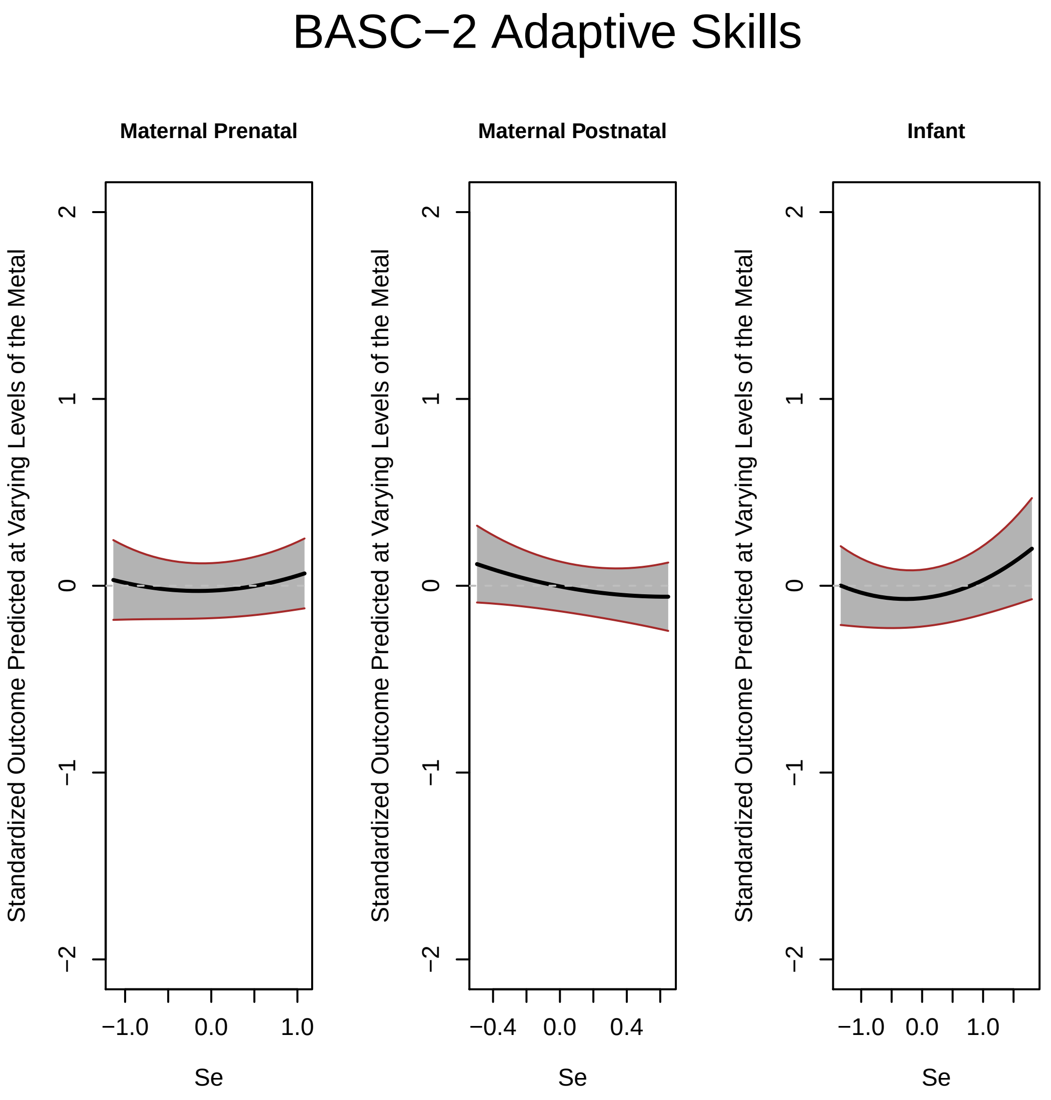
BASC−2 Adaptive Skills
Maternal Prenatal
Maternal Postnatal
Infant
Standardized Outcome Predicted at Varying Levels of the Metal
2
1
0
−1
−2
−1.0
0.0
1.0
−0.4
0.0
0.4
Se

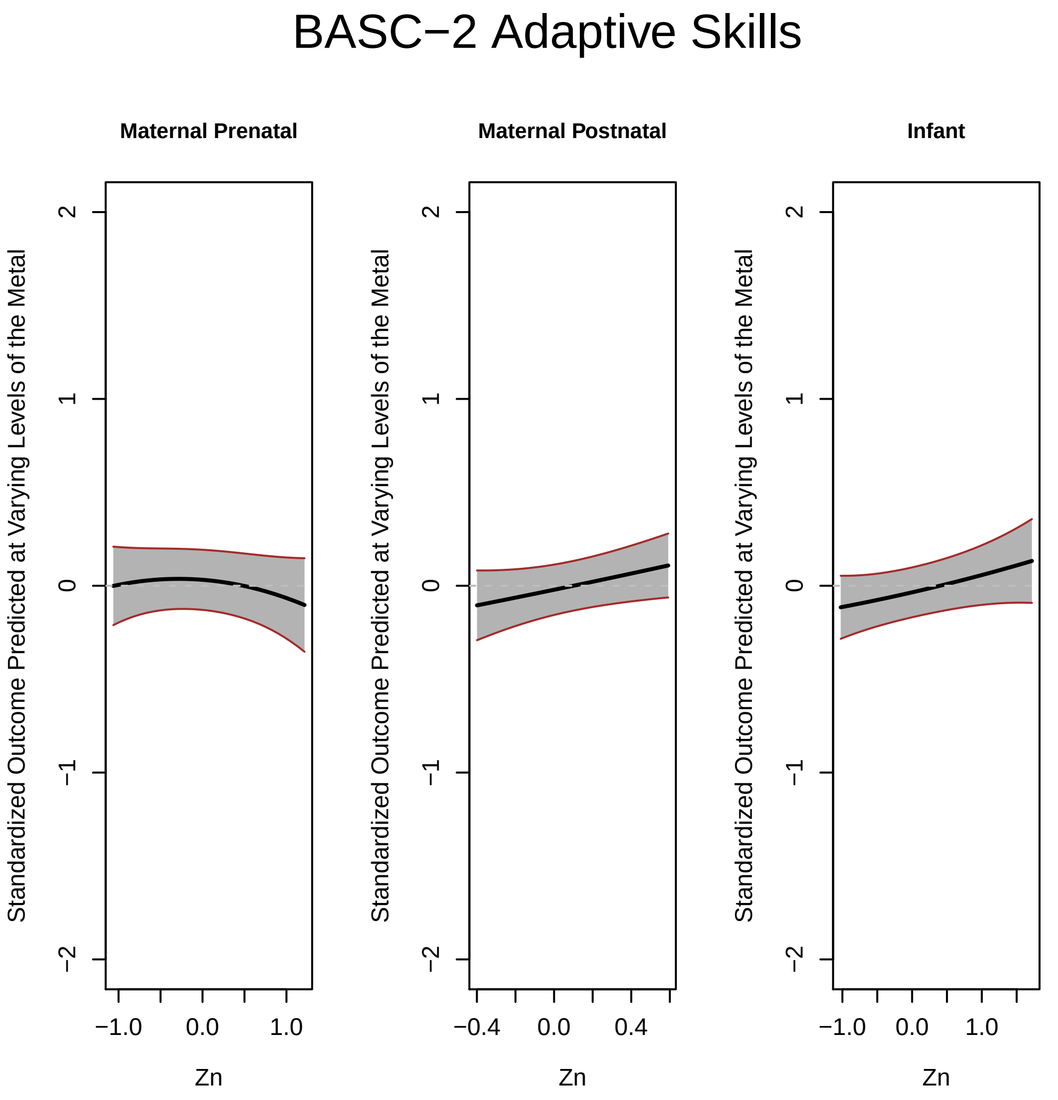

BASC−2 Adaptive Skills
Maternal Prenatal
Maternal Postnatal
Infant
Standardized Outcome Predicted at Varying Levels of the Metal
2
1
0
−1
−2
−1.0
0.0
1.0
−0.4
0.0
0.4
Zn
