# Supplementary material for: Periconceptional and prenatal exposure to metal mixtures in relation to behavioral development at 3 years of age
Source: Environ Epidemiol. 2020 Jul 6;4(4):e0106. doi: 10.1097/EE9.0000000000000106 (PMC7595192; doi:10.1097/EE9.0000000000000106)
Supplement: Supplementary file 3 [file ee9-4-e0106-s003.docx]

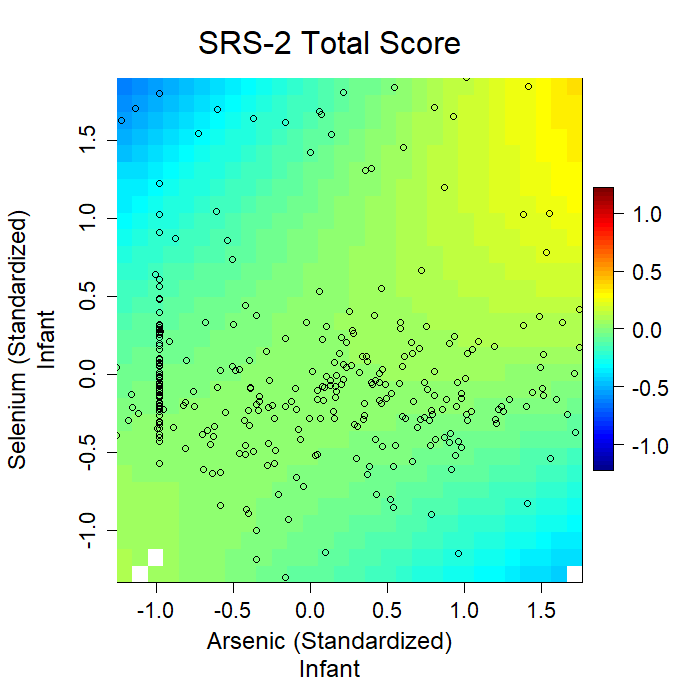


Supplemental Figure 3. Exposure-response surface of predicted SRS-2 Total Scores as a function of infant toenail concentrations of As and Se, with other metals fixed at their medians and adjusted for maternal age (quadratic), maternal BMI (quadratic), highest level of parental education (high school or less, any college, any graduate), sex (male, female), parity (0, ≥1), smoking status (no second- or first-hand, ever second-hand only, ever first-hand), age at last breastfeeding (<365 days, ≥365 days), maternal marital status (married, other), birthyear (2010-2011, 2012-2013, 2014-2015), Healthy Eating Index (linear), Parenting Relationship Questionnaire (first three principal components), and age at assessment (linear).


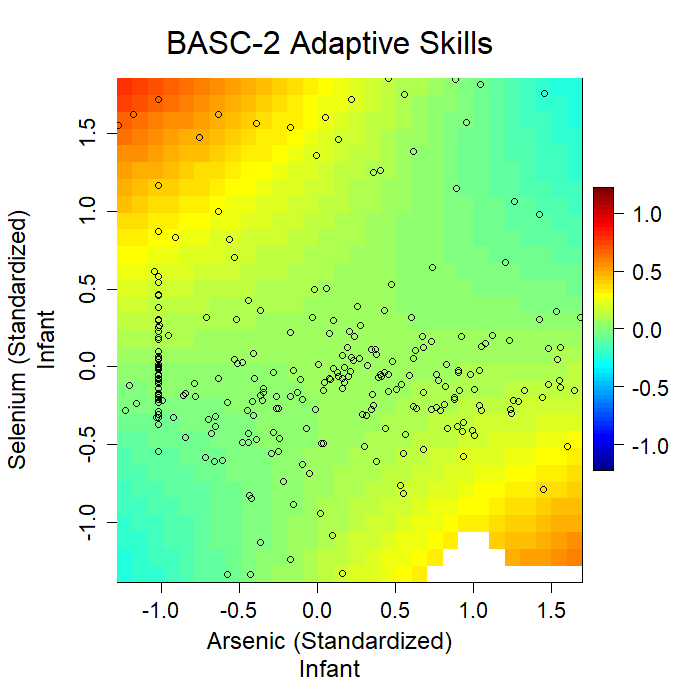


Supplemental Figure 4. Exposure-response surface of predicted BASC-2 Adaptive Skills Scores as a function of infant toenail concentrations of As and Se, with other metals fixed at their medians and adjusted for maternal age (quadratic), maternal BMI (quadratic), highest level of parental education (high school or less, any college, any graduate), sex (male, female), parity (0, ≥1), smoking status (no second- or first-hand, ever second-hand only, ever first-hand), age at last breastfeeding (<365 days, ≥365 days), maternal marital status (married, other), birthyear (2010-2011, 2012-2013, 2014-2015), Healthy Eating Index (linear), Parenting Relationship Questionnaire (first three principal components), and age at assessment (linear).


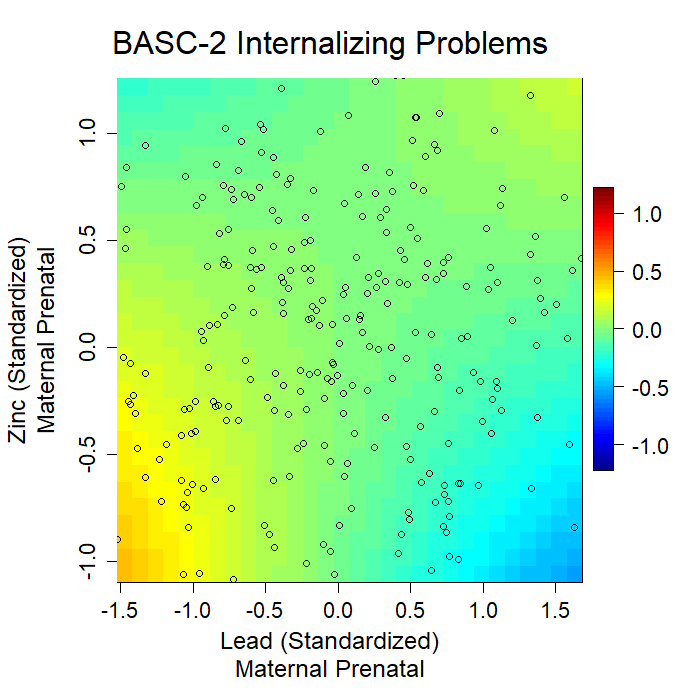


Supplemental Figure 5. Exposure-response surface of predicted BASC-2 Internalizing Problems Scores as a function of maternal prenatal toenail concentrations of As and Se, with other metals fixed at their medians and adjusted for maternal age (quadratic), maternal BMI (quadratic), highest level of parental education (high school or less, any college, any graduate), sex (male, female), parity (0, ≥1), smoking status (no second- or first-hand, ever second-hand only, ever first-hand), age at last breastfeeding (<365 days, ≥365 days), maternal marital status (married, other), birthyear (2010-2011, 2012-2013, 2014-2015), Healthy Eating Index (linear), Parenting Relationship Questionnaire (first three principal components), and age at assessment (linear).
